# Green functions and smooth distances

**DOI:** 10.1007/s00208-023-02715-6

**Published:** 2023-09-05

**Authors:** Joseph Feneuil, Linhan Li, Svitlana Mayboroda

**Affiliations:** 1 Mathematical Sciences Institute, Australian National University, Acton, ACT Australia; 2 School of Mathematics, The University of Edinburgh, Edinburgh, UK; 3 School of Mathematics, University of Minnesota, Minneapolis, MN 55455 USA

**Keywords:** 35J25, 42B37, 31B25

## Abstract

In the present paper, we show that for an optimal class of elliptic operators with non-smooth coefficients on a 1-sided Chord-Arc domain, the boundary of the domain is uniformly rectifiable if and only if the Green function *G* behaves like a distance function to the boundary, in the sense that $\left| \frac{\nabla G(X)}{G(X)}-\frac{\nabla D(X)}{D(X)}\right| ^2D(X) dX$ is the density of a Carleson measure, where *D* is a regularized distance adapted to the boundary of the domain. The main ingredient in our proof is a corona decomposition that is compatible with Tolsa’s $\alpha$-number of uniformly rectifiable sets. We believe that the method can be applied to many other problems at the intersection of PDE and geometric measure theory, and in particular, we are able to derive a generalization of the classical F. and M. Riesz theorem to the same class of elliptic operators as above.

## Introduction

### Motivation and predecessors

We consider elliptic operators *L* on a domain $\Omega \subset \mathbb {R}^n.$ In recent years a finale of an enormous body of work brought a characterization of uniform rectifiability in terms of absolute continuity of harmonic measure (see [4], a sample of earlier articles: [2, 6, 17, 34, 35, 39], see also the related article [48] which proves the David-Semmes conjecture in codimension 1 and is a key step for the converse established in [6]). It also became clear that this characterization has its restrictions, for it fails in the domains with lower dimensional boundaries and it requires, in all directions, restrictions on the coefficients—see a discussion in [30]. In these contexts, the Green function emerged as a more suitable object to define the relevant PDE properties. Already the work in [1] and [30] suggested a possibility of a Green function characterization of the regularity of sets. However, factually, [30] provided more than satisfactory “free boundary” results and only weak “direct” results (no norm control). The papers [25, 27, 28] aimed at the desired quantitative version of such “direct” results but were restricted to either Lipschitz graphs or sets with lower dimensional boundaries. The primary goal of the article is to show that if *L* is reasonably well-behaved, and $\Omega$ provides some access to its boundary, then the boundary of $\Omega$ is reasonably regular (uniformly rectifiable) if and only if the Green function behaves like a distance to the boundary.

Let us discuss some predecessors of this work, including the aforementioned ones, in more details. In [1, Theorem VI], it is shown that the affine deviation of the Green function for the Laplace operator is related to the linear deviation of the boundary of the domain. In [30], David and Mayboroda show that for a class of elliptic operators, the Green function can be well approximated by distances to planes, or by a smooth distance to $\partial \Omega ,$ if and only $\partial \Omega$ is uniformly rectifiable. The bounds on the Green function given in [30] are weak, more precisely, they carry no norm control of the sets where the Green function is close to a distance. Later, stronger and quantitative estimates on the comparison of the Green function and some distance functions are obtained in [25, 27, 28]. In [27], a quantitative comparison between the Green function and the distance function to the boundary is given for an optimal class of elliptic operators on the upper half-space. Moreover, the proximity of the Green function and the distance function is shown to be precisely controlled by the oscillation of the coefficients of the operator. Next, [28] extends the result of [27] to $\mathbb {R}^n{\setminus }\mathbb {R}^d$ with *d* strictly less than *n*. But the methods employed in [27, 28] seem to the authors difficult to be adapted to domains whose boundaries are rougher than Lipschitz graphs. In [25], a bound for the difference of the Green function and smooth distances is obtained for sets with uniformly rectifiable boundaries, but its proof, which might appear surprising, is radically dependent on the fact that the boundary is of codimension strictly larger than 1. Also, the class of operators considered in [25] is not optimal. So the instant motivation for the present work is to obtain a strong estimate on the Green function for an optimal class of operators, similar to the one considered in [27], in a “classical” setting: on domains with uniformly rectifiable boundaries of codimension 1. The method employed here is completely different from [25] or [27], and has the potential to be applicable to many other problems at the intersection of PDE and geometric measure theory.

We should also mention that in [27, 40], some Carleson measure estimates on the *second derivatives* of the Green function have been obtained, and that in [1], the second derivative of the Green function for the Laplace operator is linked to the regularity (uniform rectifiability) of the boundary of the domain. However, the result of [1] is only for the Laplace operator, the class of elliptic operators considered in [40] is more general but still not optimal, and the estimates obtained in [27] are restricted to Lipschitz graph domains. We think our estimates might shed some light on proving an estimate on second derivatives of the Green function for an optimal class of elliptic operators on chord-arc domains.

For the “free boundary” direction, since the weak type property of the Green function considered in [30] already implies uniform rectifiability, one expects the strong estimate on the Green function that we consider in this paper to automatically give uniform rectifiability. However, linking the two conditions directly seems to be more subtle than it might appear, and we actually need to obtain uniform rectifiability from scratch. We point out that our result also holds for bounded domains, and thus dispensing with the unboundedness assumption on the domain in [30].

All in all, this paper is a culmination of all of the aforementioned efforts, featuring a true equivalence (characterization) of geometry through PDEs, and an optimal class of operators.

### Statements of the main results

We take a domain $\Omega \subset \mathbb {R}^n$ whose boundary $\partial \Omega$ is $(n-1)$-Ahlfors regular (AR for shortness), which means that there exists a measure $\sigma$ supported on $\partial \Omega$ such that

1.1 $$\begin{aligned} C_{\sigma }^{-1}r^{n-1} \le \sigma (B(x,r)) \le C_{\sigma } r^{n-1} \quad \text {for } x\in \partial \Omega , \ r \in (0, {{\,\textrm{diam}\,}}\Omega ). \end{aligned}$$

The domain $\Omega$ can be bounded or unbounded. In the unbounded case, ${{\,\textrm{diam}\,}}\Omega =\infty .$ In the rest of the paper, $\sigma$ will always be an Ahlfors regular measure on $\partial \Omega .$ It is known that the Ahlfors regular measures are the ones that can be written as $d\sigma = w d{\mathcal {H}}^{n-1}|_{\partial \Omega },$ where ${\mathcal {H}}^{n-1}|_{\partial \Omega }$ is the $n-1$ dimensional Hausdorff measure on $\partial \Omega ,$ and *w* is a weight in $L^\infty (\partial \Omega , {\mathcal {H}}^{n-1}|_{\partial \Omega })$ such that $C^{-1} \le w \le C$ for some constant $C>0.$

We shall impose more assumptions on our domain. For both the “free boundary” and the “direct” results, we will assume that $\Omega$ is a 1-sided Chord Arc Domain (see Definition 2.11). For the “direct” result, we will rely on the assumption that $\partial \Omega$ is uniformly rectifiable (see [18, 19] and Sect. 3 below), and thus ultimately assuming that $\Omega$ is a (2-sided) Chord Arc Domain. The optimality of the assumptions on $\Omega$ is discussed in more detail at the end of this subsection. Since the dimension $n-1$ plays an important role in our paper, and in order to lighten the notion, we shall write *d* for $n-1.$

Without any more delay, let us introduce the regularized distance to a set $\partial \Omega .$ The Euclidean distance to the boundary is denoted by

1.2 $$\begin{aligned} \delta (X):= {{\,\textrm{dist}\,}}(X,\partial \Omega ). \end{aligned}$$

For $\beta >0,$ we define

1.3 $$\begin{aligned} D_{\beta }(X):= \left( \int _{\partial \Omega } |X-y|^{-d-\beta } d\sigma (y) \right) ^{-1/\beta } \quad \text {for } X\in \Omega . \end{aligned}$$

The fact that the set $\partial \Omega$ is *d*-Ahlfors regular is enough to have that

1.4 $$\begin{aligned} C^{-1} \delta (X) \le D_{\beta }(X) \le C\delta (X) \quad \text {for } X\in \Omega , \end{aligned}$$

where the constant depends on $C_{\sigma },$ $\beta ,$ and *n*. The proof is easy and can be found after Lemma 5.1 in [23].

The notion of Carleson measure will be central all over our paper. We say that a quantity *f* defined on $\Omega$ satisfies the Carleson measure condition—or $f\in CM_{\Omega }(M)$ for short—if there exists *M* such that for any $x\in \partial \Omega$ and $r<{{\,\textrm{diam}\,}}(\Omega ),$

1.5 $$\begin{aligned} \iint _{B(x,r) \cap \Omega } |f(X)|^2 \delta (X)^{-1} dX \le M r^{n-1}. \end{aligned}$$

Our operators are in the form $L=-\mathop {{\text {div}}}\mathcal {A}\nabla$ and defined on $\Omega .$ We shall always assume that they are uniformly elliptic and bounded, that is, there exists $C_{\mathcal {A}}>1$ such that

1.6 $$\begin{aligned} \mathcal {A}(X)\xi \cdot \xi \ge C_{\mathcal {A}}^{-1} |\xi |^2 \quad \text {for } X\in \Omega , \ \xi \in \mathbb {R}^n, \end{aligned}$$

and

1.7 $$\begin{aligned} |\mathcal {A}(X)\xi \cdot \zeta | \le C_{\mathcal {A}} |\xi ||\zeta | \quad \text {for } X\in \Omega , \ \xi ,\zeta \in \mathbb {R}^n. \end{aligned}$$

A weak solution to $Lu=0$ in $E \subset \Omega$ lies in $W^{1,2}_{loc}(E)$ and is such that

1.8 $$\begin{aligned} \int _{\Omega } \mathcal {A}\nabla u \cdot \nabla \varphi \, dX=0 \quad \text {for } \varphi \in C^\infty _0(E). \end{aligned}$$

If $\Omega$ has sufficient access to the boundary (and $\partial \Omega$ is $(n-1)$-Ahlfors regular), then for any ball *B* centered on $\partial \Omega$ and any function *u* in $W^{1,2}(B\cap \Omega ),$ we have notion of trace for *u* on $B\cap \partial \Omega .$ It is well known that if $u \in W^{1,2}(B\cap \Omega )$ is such that ${{\,\textrm{Tr}\,}}(u) = 0$ on $B\cap \partial \Omega ,$ and if *u* is a weak solution to $Lu=0$ on $B\cap \Omega$ with *L* satisfying (1.6) and (1.7), then *u* is continuous $B\cap \Omega$ and can be continuously extended by 0 on $B\cap \partial \Omega .$

In addition to (1.6) and (1.7), we assume that our operators satisfy a weaker variant of the Dahlberg–Kenig–Pipher condition. The Dahlberg–Kenig–Pipher (DKP) condition was introduced by Dahlberg and shown to be sufficient for the $L^p$ solvability of the Dirichlet problem for some $p>1$ by Kenig and Pipher ([44]). It was also shown to be essentially necessary in [10, 45]. The DKP condition says that the coefficient matrix $\mathcal {A}$ satisfies

1.9 $$\begin{aligned} \delta (\cdot )\sup _{B(\cdot ,\,\delta (\cdot )/2)}\left| \nabla \mathcal {A}\right| \in CM_\Omega (M) \quad \text {for some }M<\infty . \end{aligned}$$

Our assumption, slightly weaker than the classical DKP, is as follows.

#### Definition 1

(*Weak DKP condition*) An elliptic operator $L=-\mathop {{\text {div}}}\mathcal {A}\nabla$ is said to satisfy the weak DKP condition with constant *M* on $\Omega$ if there exists a decomposition $\mathcal {A}= \mathcal {B}+ \mathcal {C}$ such that

1.11 $$\begin{aligned} |\delta \nabla \mathcal {B}| + |\mathcal {C}| \in CM_{\Omega }(M). \end{aligned}$$

Obviously, this condition is weaker than (1.9): it allows for small Carleson perturbations and carries no supremum over the Whitney cubes. Moreover, we show in Lemma 2.1 that the weak DKP condition self improves.

We are now ready for the statement of our main result.

#### Theorem 2

Let $\beta >0,$ $\Omega \subset \mathbb R^n$ be a 1-sided Chord-Arc Domain, and $L=-\mathop {{\text {div}}}\mathcal {A}\nabla$ be a uniformly elliptic operator—i.e.,  that verifies (1.6) and (1.7)—which satisfies the weak DKP condition with constant *M* on $\Omega .$ We write $G^{X_0}$ for the Green function of *L* with pole at $X_0.$ The following are equivalent : 

(i) $\Omega$ is a Chord-Arc Domain, 
(ii) $\partial \Omega$ is uniformly rectifiable, 
(iii) there exists $C\in (0,\infty )$ such that for any ball *B* centered on the boundary,  and for any positive weak solution *u* to $Lu=0$ in $2B \cap \Omega$ for which ${{\,\textrm{Tr}\,}}u = 0$ on $2B \cap \partial \Omega ,$ we have
  1.13 $$\begin{aligned} \iint _{\Omega \cap B} \left| \frac{\nabla u}{u} - \frac{\nabla D_{\beta }}{D_{\beta }} \right| ^2 D_{\beta } \, dX \le C \sigma (B), \end{aligned}$$
(iv) there exists $C\in (0,\infty )$ such that for any $X_0 \in \Omega$ and for any ball *B* centered on the boundary satisfying $X_0 \notin 2B,$ we have
  1.14 $$\begin{aligned} \iint _{\Omega \cap B} \left| \frac{\nabla G^{X_0}}{G^{X_0}} - \frac{\nabla D_{\beta }}{D_{\beta }} \right| ^2 D_{\beta } \, dX \le C \sigma (B), \end{aligned}$$
(v) there exist $X_0\in \Omega$ and $C\in (0,\infty )$ such that for any ball *B* centered on the boundary that satisfies $X_0\notin 2B,$ we have (1.14).

Moreover,  the constants *C* in (1.13)–(1.14) can be chosen to depend only on $C_\mathcal {A},$ *M*,  the CAD constants of $\Omega ,$ $\beta ,$ and *n*.

#### Remark 1.15

The bound (1.13) is a local one, meaning for instance that the bound will hold with a constant *C* independent of *B* and the solution *u* as long as $\Omega$ is chord-arc locally in 2*B* (that is, we only need the existence of Harnack chains and of corkscrew points in $2B\cap \Omega )$ and the uniformly elliptic operator *L* satisfies the weak DKP condition in 2*B*.

The equivalence $(\text {i}) \Longleftrightarrow (\text {ii})$ is already well known, see Theorem 1.2 in [5]. Moreover, $(\text {iii}) \implies (\text {iv}) \implies (\text {v})$ is immediate. So we need only to prove $(\text {ii})\implies (\text {iii})$ and $(\text {v})\implies (\text {i})$ in Theorem 1.12.

When the domain is unbounded, we can use the Green function with pole at infinity instead of the Green function. The Green function with pole at infinity associated to *L* is the unique (up to a multiplicative constant) positive weak solution to $Lu=0$ with zero trace. See for instance Lemma 6.5 in [21] for the construction ([21] treats a particular case but the same argument works as long as we have CFMS estimates, see Lemma 2.18 below). So we have that:

#### Corollary 4

Let $\beta ,$ $\Omega$ and *L* be as in Theorem 1.12. If $\Omega$ is unbounded,  the following are equivalent : 

- $\Omega$ is a Chord-Arc Domain, 
- $\partial \Omega$ is uniformly rectifiable, 
- there exists $C\in (0,\infty )$ such that for any ball *B* centered on the boundary,  we have
  1.17 $$\begin{aligned} \iint _{\Omega \cap B} \left| \frac{\nabla G^{\infty }}{G^{\infty }} - \frac{\nabla D_{\beta }}{D_{\beta }} \right| ^2 D_{\beta } \, dX \le C \sigma (B), \end{aligned}$$

For our proof of the “direct” result, we need the fact that, for the same operators, the *L*-elliptic measure is $A_{\infty }$-absolutely continuous with respect to $\sigma .$

#### Theorem 5

Let $\Omega$ be a Chord-Arc Domain, and let $L=-\mathop {{\text {div}}}\mathcal {A}\nabla$ be a uniformly elliptic operator—i.e.,  that verifies (1.6) and (1.7)—which satisfies the weak DKP condition with constant *M* on $\Omega .$

Then the *L*-elliptic measure $\omega _L\in A_{\infty }(\sigma ),$ i.e. there exists $C,\theta >0$ such that given an arbitrary surface ball $\Delta =B\cap \partial \Omega ,$ with $B=B(x,r),$ $x\in \partial \Omega ,$ $0<r<{{\,\textrm{diam}\,}}( \partial \Omega ),$ and for every Borel set $F\subset \Delta ,$ we have that

1.19 $$\begin{aligned} \frac{\sigma (F)}{\sigma (\Delta )} \le C \left( \omega _L^{X_{\Delta }}(F)\right) ^{\theta }, \end{aligned}$$

where $X_{\Delta }$ is a corkscrew point relative to $\Delta$ (see Definition 2.8).

The constants *C* and $\theta$—that are called the intrinsic constants in $\omega _L \in A_{\infty }(\sigma )$—depend only on $C_\mathcal {A},$ *M*,  the CAD constants of $\Omega ,$ and *n*.

The above is known for operators satisfying the DKP condition (1.9) on Chord-Arc domains. In fact, it is shown in [44] that $\omega _L\in A_{\infty }(\sigma )$ for DKP operators on Lipschitz domains. But since the DKP condition is preserved in subdomains, and the Chord-Arc domains are well approximated by Lipschitz subdomains [17], the $A_{\infty }$ property can be passed from Lipschitz subdomains to Chord-Arc domains (see [42], or [38]). Moreover, combined with the stability of the $A_{\infty }$ property under Carleson perturbation of elliptic operators proved in [13], it is also known for the elliptic operators $L=-{{\,\textrm{div}\,}}\mathcal {A}\nabla$ for which $\mathcal {A}= \mathcal {B}+ \mathcal {C},$ where

1.20 $$\begin{aligned} \sup _{B(\cdot ,\,\delta (\cdot )/2)}\{\left| \delta \nabla \mathcal {B}\right| + \left| \mathcal {C}\right| \} \in CM_\Omega (M) \quad \text {for some }M<\infty . \end{aligned}$$

However, to the best of our knowledge, the $A_{\infty }$-absolute continuity of the elliptic measure was not proved explicitly for elliptic operators satisfying the slightly weaker condition (1.11).

We obtain Theorem 1.18 as a consequence of the following result—which is another contribution of the article—and Theorem 1.1 in [13].

#### Theorem 6

Let $\Omega$ be a domain in $\mathbb {R}^n$ with uniformly rectifiable (UR) boundary of dimension $n-1.$ Let *L* be a uniformly elliptic operator which satisfies the weak DKP condition with constant *M* on $\Omega .$ Suppose that *u* is a bounded solution of $Lu=0$ in $\Omega .$ Then for any ball *B* centered on the boundary,  we have

1.22 $$\begin{aligned} \iint _{\Omega \cap B}\left| \nabla u(X)\right| ^2\delta (X)dX\le C\left\| u\right\| _{L^\infty (\Omega )}^2\sigma (B\cap \partial \Omega ), \end{aligned}$$

where the constant *C* depends only on *n*,  *M*,  and the UR constants of $\partial \Omega .$

Notice that in this theorem, we completely dispense with the Harnack chain and corkscrew conditions (see Definitions 2.8 and 2.9) for the domain. Previously, an analogous result was obtained for bounded *harmonic functions* in [36] (see also [33] for the converse) and for DKP operators in [37, Theorem 7.5]. The result for elliptic operators which satisfy the weak DKP condition is again not explicitly written anywhere. However, slightly changing the proofs of a combination of papers would give the result. For instance, we can adapt Theorem 1.32 in [23] to the codimension 1 case to prove Theorem 1.18 in the flat case, then extending it to Lipschitz graph by using the change of variable in [44], and finally proving Theorem 1.18 for all complements of uniformly rectifiable domains by invoking Theorem 1.19 (iii) in [37]).

Here, we claim that we can directly demonstrate Theorem 1.21 using a strategy similar to our proof of Theorem 1.12. In Sect. 8, we explain how to modify the proof of Theorem 1.12 to obtain Theorem 1.21. By [13] Theorem 1.1, assuming that $\Omega$ is 1-sided CAD, the estimate (1.22) implies that $\omega _L\in A_{\infty }(\sigma ),$ and therefore our Theorem 1.18 follows from Theorem 1.21. Note that the bound (1.19) is a characterization of $A_{\infty },$ see for instance Theorem 1.4.13 in [43].

Let us discuss in more details our assumptions for Theorem 1.12. In order to get the bound (1.13), we strongly require that the boundary $\partial \Omega$ is uniformly rectifiable and that the operator *L* satisfies the weak DKP condition. We even conjecture that those conditions are necessary, that is, if $\partial \Omega$ is not uniformly rectifiable, then the bound (1.13) holds for none of the weak DKP operators.

The corkscrew condition and the Harnack chain condition (see Definitions 2.8 and 2.9) are only needed for the proof of Lemma 7.12—where we used the comparison principle—and for the implication $(\text {iii})\implies (\text {i})$ in Theorem 1.12. However, since most of our intermediate results can be proved without those conditions and could be of interest in other situations where the Harnack chain is not assumed (like—for instance—to prove Theorem 1.21), we avoided to use the existence of Harnack chains and of corkscrew points in all the proofs and the intermediate results except for Lemma 7.12 and in Sect. 9, even if it occasionally slightly complicated the arguments.

These observations naturally lead to the question about the optimality of our conditions on $\Omega ,$ and more precisely, whether we can obtain the estimate (1.13) assuming only uniform rectifiability. The answer is no, as we can construct a domain $\Omega$ which has uniformly rectifiable boundary but is only semi-uniform (see Definition 10.1) where (1.13) fails. More precisely, we prove in Sect. 10 that:

#### Proposition 7

There exists a semi-uniform domain $\Omega$ and a positive harmonic function *G* on $\Omega$ such that (1.13) is false.

But of course, assuming $\Omega$ is a Chord-Arc Domain is not necessary for (1.13) since (1.13) obviously holds when $\Omega = \mathbb {R}^n {\setminus } \mathbb {R}^{n-1} = \mathbb {R}^n_+ \cup \mathbb {R}^n_-,$ because we can apply Theorem 1.12 to both $\Omega _+ = \mathbb {R}^n_+$ and $\Omega _- = \mathbb {R}^n_-$ and then sum.

### Main steps of the proof of $(\text {ii}) \implies (\text {iii})$

In this section, we present the outline of the proof of $(\text {ii}) \implies (\text {iii})$ in Theorem 1.12. More exactly, this subsection aims to link the results of all other sections of the paper in order to provide the proof.

The approach developed in this article is new and it is interesting by itself, because it gives an alternative to proofs that use projection and extrapolation of measures. Aside from Theorems 1.12 and 1.21, we claim that our approach can be used to obtain a third proof of the main result from [29, 31], which establishes the $A_{\infty }$-absolute continuity of the harmonic measure when $\Omega$ is the complement of a uniformly rectifiable set of low dimension and *L* is a properly chosen degenerate operator.

Let $\Omega$ and *L* be as in the assumptions of Theorem 1.12, and let $\mathcal {B}$ and $\mathcal {C}$ denote the matrices in (1.11). Take $B := B(x_0,r)$ to be a ball centered at the boundary, that is $x_0 \in \partial \Omega ,$ and then a non-negative weak solution *u* to $Lu=0$ in $2B \cap \Omega$ such that ${{\,\textrm{Tr}\,}}(u) = 0$ on $B \cap \partial \Omega .$

**Step 1: From balls to dyadic cubes.** We construct a dyadic collection ${\mathbb {D}}_{\partial \Omega }$ of pseudo-cubes in $\partial \Omega$ in the beginning of Sect. 3, and a collection of Whitney regions $W_{\Omega }(Q),W_{\Omega }^*(Q)$ associated to $Q\in {\mathbb {D}}_{\partial \Omega }$ in the beginning Sect. 4. We claim that (1.13) is implied by the following estimate that uses dyadic cubes

1.24 $$\begin{aligned} I := \sum _{{\begin{array}{c} Q \in {\mathbb {D}}_{\partial \Omega } \\ Q \subset Q_0 \end{array}}} \iint _{W_{\Omega }(Q)} \left| \frac{\nabla u}{u} - \frac{\nabla D_{\beta }}{D_{\beta }} \right| ^2 \delta \, dX \le C \sigma (Q_0) \end{aligned}$$

for any cube $Q_0\in {\mathbb {D}}_{\partial \Omega }$ satisfying $Q_0 \subset \frac{8}{7}B \cap \partial \Omega$ and $\ell (Q_0) \le 2^{-8}r.$ It follows from the definition of $W^*_{\Omega }(Q)$ (4.5) that

1.25 $$\begin{aligned} W^*_{\Omega }(Q) \subset \frac{7}{4}B \quad \text {for } Q \subset Q_0 \end{aligned}$$

and $Q_0$ as above.

We take $\{Q_0^i\}\subset {\mathbb {D}}_{\partial \Omega }$ as the collection of dyadic cubes that intersect $B \cap \partial \Omega$ and such that $2^{-9}r < \ell (Q_0^i) \le 2^{-8}r.$ There is a uniformly bounded number of them, each of them satisfies $Q_0^i \subset \frac{3}{2}B \cap \Omega$ and $\ell (Q_0^i) \le 2^{-8}r$ and altogether, they verify

$$\begin{aligned} B \cap \Omega \subset \{X\in B, \, \delta (X) > 2^{-9}r\} \bigcup \Bigl (\bigcup _{i} \bigcup _{Q\in {\mathbb {D}}_{\partial \Omega }(Q_0^i)} W_\Omega (Q) \Bigr ). \end{aligned}$$

The estimate (1.13) follows by applying (1.24) to each $Q_0^i$—using (1.4) and (1.1) when needed—and (1.26) below to $\{X\in B, \, \delta (X) > 2^{-9}r\}.$

**Step 2: Bound on a Whitney region.** In this step, we establish that if $E\subset \frac{7}{4}B$ is such that ${{\,\textrm{diam}\,}}(E) \le K\delta (E),$ then

1.26 $$\begin{aligned} J_E:= \iint _{E} \left| \frac{\nabla u}{u} - \frac{\nabla D_{\beta }}{D_{\beta }} \right| ^2 \delta \, dX \le C_K \delta (E)^{n-1}. \end{aligned}$$

We could use Lemma 7.9 to prove this, but it would be like using a road roller to crack a nutshell, because it is actually easy. We first separate

$$\begin{aligned} J_E \le \iint _{E} \left| \frac{\nabla u}{u}\right| ^2 \delta \, dX + \iint _{E} \left| \frac{\nabla D_{\beta }}{D_{\beta }} \right| ^2 \delta \, dX := J^1_E + J^2_E. \end{aligned}$$

We start with $J_E^2.$ Observe that $|\nabla [D_{\beta }^{-\beta }]| \le (d+\beta ) D_{\beta +1}^{-\beta -1},$ so $|\frac{\nabla D_{\beta }}{D_{\beta }}| \lesssim \delta ^{-1}$ by (1.4). We deduce that $J_E^2 \lesssim |E| \delta (E)^{-1} \lesssim \delta (E)^{n-1}$ as desired. As for $J_E^1,$ we construct

$$\begin{aligned} E^*:= \{X\in \Omega , {{\,\textrm{dist}\,}}(X,E) \le \delta (E)/100\}\subset \frac{15}{8}B, \end{aligned}$$

and then the Harnack inequality (Lemma 2.15) and the Caccioppoli inequality (Lemma 2.14) yield that

$$\begin{aligned} J_E^1\lesssim & {} \delta (E) (\sup _{E^*} u)^{-2} \iint _{E} |\nabla u|^2 dX \lesssim \delta (E)^{-1} (\sup _{E^*} u)^{-2} \iint _{E^*} u^2 dX \\\le & {} \delta (E)^{-1}|E^*| \lesssim \delta (E)^{n-1}. \end{aligned}$$

The bound (1.26) follows.

**Step 3: Corona decomposition.** Let $Q_0$ as in Step 1. One can see that we cannot apply (1.26) to each $E=W_{\Omega }(Q)$ and still hope to get the bound (1.24) for *I*. We have to use (1.26) with parsimony. We first use a corona decomposition of $D_{\partial \Omega }(Q_0),$ and we let the stopping time region stop whenever $\alpha (Q)$ or the angle between the approximating planes are too big. We choose $0 < \epsilon _1 \ll \epsilon _0 \ll 1$ and Lemma 3.16 provides a first partition of ${\mathbb {D}}_{\partial \Omega }$ into bad cubes $\mathcal {B}$ and good cubes ${\mathcal {G}}$ and then a partition of ${\mathcal {G}}$ into a collection of coherent regimes $\{\mathcal {S}\}_{\mathcal {S}\in {\mathfrak {S}}}.$

Let $\mathcal {B}(Q_0):= \mathcal {B}\cap {\mathbb {D}}_{\partial \Omega }(Q_0)$ and then ${\mathfrak {S}}(Q_0) = \{\mathcal {S}\cap {\mathbb {D}}_{\partial \Omega }(Q_0)\}.$ Observe that ${\mathfrak {S}}(Q_0)$ contains the collection of $\mathcal {S}\in {\mathfrak {S}}$ such that $Q(\mathcal {S}) \subset Q_0$ and maybe **one** extra element, in the case where $Q_0 \notin \mathcal {B}\cup \bigcup _{\mathcal {S}\in {\mathfrak {S}}} Q(\mathcal {S}),$ which is the intersection with ${\mathbb {D}}_{\partial \Omega }(Q_0)$ of the coherent regime $\mathcal {S}\in {\mathfrak {S}}$ that contains $Q_0.$ In any case ${\mathfrak {S}}(Q_0)$ is a collection of (stopping time) coherent regimes. In addition, Lemma 3.16 shows that ${\mathfrak {S}}(Q_0)$ and $\mathcal {B}(Q_0)$ verifies the Carleson packing condition

1.27 $$\begin{aligned} \sum _{Q\in \mathcal {B}(Q_0)} \sigma (Q) + \sum _{\mathcal {S}\in {\mathfrak {S}}(Q_0)} \sigma (Q(\mathcal {S})) \le C \sigma (Q_0). \end{aligned}$$

We use this corona decomposition to decompose the sum *I* from (1.24) as

1.28 $$\begin{aligned} I= & {} \sum _{Q \in \mathcal {B}(Q_0)} \iint _{W_{\Omega }(Q)} \left| \frac{\nabla u}{u} - \frac{\nabla D_{\beta }}{D_{\beta }} \right| ^2 \delta \, dX \nonumber \\{} & {} + \sum _{\mathcal {S}\in {\mathfrak {S}}(Q_0)} \iint _{W_{\Omega }(\mathcal {S})} \left| \frac{\nabla u}{u} - \frac{\nabla D_{\beta }}{D_{\beta }} \right| ^2 \delta \, dX := I_1 + \sum _{\mathcal {S}\in {\mathfrak {S}}(Q_0)} I_{\mathcal {S}}, \end{aligned}$$

where $W_{\Omega }(\mathcal {S}) = \bigcup _{Q\in \mathcal {S}} W_{\Omega } (Q).$ For each cube $Q\in \mathcal {B}(Q_0),$ the regions $W_{\Omega }(Q)$ are included in $\frac{7}{4}B$ and verify ${{\,\textrm{diam}\,}}(W_{\Omega }(Q)) \le 8 \delta (W_{\Omega }(Q)) \le 8\ell (Q),$ so we can use (1.26) and we obtain

1.29 $$\begin{aligned} I_1 \lesssim \sum _{Q\in \mathcal {B}(Q_0)} \ell (Q)^{n-1} \lesssim \sigma (Q_0). \end{aligned}$$

by (1.1) and (1.27).

**Step 4: How to turn the estimation of** $I_{\mathcal {S}}$ **into a problem on** $\mathbb {R}^n_+.$ Now, we take $\mathcal {S}$ in ${\mathfrak {S}}(Q_0),$ which is nice because $\partial \Omega$ is well approximated by a small Lipschitz graph $\Gamma _{\mathcal {S}}$ around any dyadic cube of $\mathcal {S}$ (see Sect. 3.4 for the construction of $\Gamma _{\mathcal {S}})$. For instance, fattened versions of our Whitney regions $W^*_{\Omega }(Q),$ $Q\in \mathcal {S},$ are Whitney regions in $\mathbb {R}^n {\setminus } \Gamma _{\mathcal {S}}$ (see Lemma 4.13). More importantly, at any scale $Q\in \mathcal {S},$ the local Wasserstein distance between $\sigma$ and the Hausdorff measure of $\Gamma _{\mathcal {S}}$ is bounded by the local Wasserstein distance between $\sigma$ and the best approximating plane, which means that $\Gamma _{\mathcal {S}}$ approximate $\partial \Omega$ better (or at least not much worse) than the best plane around any $Q\in \mathcal {S}.$ Up to our knowledge, it is the first time that such a property on $\Gamma _{\mathcal {S}}$ is established. It morally means that $D_{\beta }(X)$ will be well approximated by

$$\begin{aligned} D_{\beta ,\mathcal {S}}(X):= \left( \int _{\Gamma _{\mathcal {S}}} |X-y|^{-d-\beta } d{\mathcal {H}}^{n-1}(y) \right) ^{-\frac{1}{\beta }} \end{aligned}$$

whenever $X\in X_{\Omega }(\mathcal {S}),$ and that the error can be bounded in terms of the Tolsa’s $\alpha$-numbers.

Nevertheless, what we truly want is the fact $\partial \Omega$ is well approximated by a plane—instead of a Lipschitz graph—from the standpoint of any $X \in W_{\Omega }(\mathcal {S}),$ because in this case we can use Lemma 7.9. Yet, despite this slight disagreement, $\Gamma _{\mathcal {S}}$ is much better than a random uniformly rectifiable set, because $\Gamma _{\mathcal {S}}$ is the image of a plane *P* by a bi-Lipschitz map. So we construct a bi-Lipschitz map $\rho _{\mathcal {S}} : \, \mathbb {R}^n \rightarrow \mathbb {R}^n$ that of course maps a plane to $\Gamma _{\mathcal {S}},$ but which also provides an explicit map from any point *X* in $W_{\Omega }(\mathcal {S})$ to a plane $\Lambda (\rho _{\mathcal {S}}^{-1}(X))$ that well approximates $\Gamma _{\mathcal {S}}$—hence $\partial \Omega$—from the viewpoint of *X*. So morally, we constructed $\rho _{\mathcal {S}}$ such that we have a function

$$\begin{aligned} X \mapsto {{\,\textrm{dist}\,}}(X,\Lambda (\rho ^{-1}_{\mathcal {S}}(X))) \end{aligned}$$

which, when $X\in W_{\Omega }(\mathcal {S}),$ is a good approximation of

$$\begin{aligned} D_{\beta ,\mathcal {S}}(X):= \left( \int _{\Gamma _{\mathcal {S}}} |X-y|^{-d-\beta } d{\mathcal {H}}^{n-1}(y) \right) ^{-\frac{1}{\beta }} \end{aligned}$$

in terms of the Tolsa’s $\alpha$-numbers.

We combine the two approximations to prove (see Lemma 6.30, which is a consequence of Corollary 5.52 and our construction of $\rho _{\mathcal {S}})$ that for $Q\in \mathcal {S}$

$$\begin{aligned} \iint _{W_{\Omega }(Q)} \left| \frac{\nabla D_{\beta }(X)}{D_{\beta }(X)} - \frac{N_{\rho ^{-1}_{\mathcal {S}}(X)}(X)}{{{\,\textrm{dist}\,}}(X,\Lambda (\rho ^{-1}_{\mathcal {S}}(X))} \right| ^2\, \delta (X) \, dX \le C |\alpha _{\sigma ,\beta }(Q)|^2 \sigma (Q) , \end{aligned}$$

where $Y \rightarrow N_{\rho _{\mathcal {S}}^{-1}(X)}(Y)$ is the gradient of the distance to $\Lambda (\rho _{\mathcal {S}}^{-1}(X)).$ And since the $\alpha _{\sigma ,\beta }(Q)$ satisfies the Carleson packing condition, see Lemma 5.31, we deduce that

1.30 $$\begin{aligned} I_{\mathcal {S}} \le 2I'_{\mathcal {S}} + C \sum _{Q\in \mathcal {S}} |\alpha _{\sigma ,\beta }(Q)|^2 \sigma (Q) \le 2 I'_{\mathcal {S}} + C \sigma (Q(\mathcal {S})) \end{aligned}$$

where

$$\begin{aligned} I'_{\mathcal {S}}:= \iint _{W_{\Omega }(\mathcal {S})} \left| \frac{\nabla u}{u} - \frac{N_{\rho ^{-1}_{\mathcal {S}}(X)}(X)}{{{\,\textrm{dist}\,}}(X,\Lambda (\rho ^{-1}_{\mathcal {S}}(X))} \right| ^2 \delta \, dX. \end{aligned}$$

We are left with $I'_{\mathcal {S}}.$ We make the change of variable $(p,t) = \rho ^{-1}_{\mathcal {S}}(X)$ in the integral defining $I'_{\mathcal {S}}$ and we obtain that

$$\begin{aligned} \begin{aligned} I'_{\mathcal {S}}&= \iint _{\rho _{\mathcal {S}}^{-1}(W_{\Omega }(\mathcal {S}))} \left| \frac{(\nabla u) \circ \rho _{\mathcal {S}} (p,t)}{u \circ \rho (p,t) } - \frac{N_{p,t}(\rho _{\mathcal {S}}(p,t))}{{{\,\textrm{dist}\,}}(\rho _{\mathcal {S}}(p,t),\Lambda (p,t))} \right| ^2 \delta \circ \rho _{\mathcal {S}}(p,t)\\&\quad \times \det \left( {{\,\textrm{Jac}\,}}(p,t)\right) \, dt\, dp \\&\le 2 \iint _{\rho _{\mathcal {S}}^{-1}(W_{\Omega }(\mathcal {S}))} \left| \frac{\nabla \left( u \circ \rho _{\mathcal {S}} (p,t)\right) }{u \circ \rho (p,t)} - \frac{{{\,\textrm{Jac}\,}}(p,t) N_{p,t}(\rho _{\mathcal {S}}(p,t))}{{{\,\textrm{dist}\,}}(\rho _{\mathcal {S}}(p,t),\Lambda (p,t))} \right| ^2 \, |t| \, dt\, dp, \end{aligned} \end{aligned}$$

where ${{\,\textrm{Jac}\,}}(p,t)$ is the Jacobian matrix of $\rho _{\mathcal {S}},$ which is close to the identity by Lemma 6.8. We have also used that $\delta \circ \rho _{\mathcal {S}}(p,t) \approx t$ since $\delta (X) \approx {{\,\textrm{dist}\,}}(X,\Gamma _{\mathcal {S}})$ on $W_{\Omega }(\mathcal {S})$ and the bi-Lipschitz map $\rho _{\mathcal {S}}^{-1}$ preserves this equivalence. Even if the term

$$\begin{aligned} \frac{{{\,\textrm{Jac}\,}}(p,t) N_{p,t}(\rho _{\mathcal {S}}(p,t))}{{{\,\textrm{dist}\,}}(\rho _{\mathcal {S}}(p,t),\Lambda (p,t))} \end{aligned}$$

looks bad, all the quantities inside are constructed by hand, and of course, we made them so that they are close to the quotient $\frac{\nabla d_P}{d_P},$ where $d_P$ is the distance to a plane that depends only on $\mathcal {S}.$ With our change of variable, we even made it so that $P = \mathbb {R}^{n-1} \times \{0\},$ that is $\frac{\nabla d_P}{d_P} = \frac{\nabla t}{t}.$ Long story short, Lemma 6.32 gives that

1.31 $$\begin{aligned} I'_{\mathcal {S}} \le 4I''_{\mathcal {S}} + \sigma (Q(\mathcal {S})) \end{aligned}$$

where

$$\begin{aligned} I''_{\mathcal {S}}:= \iint _{\rho ^{-1}(W_{\Omega }(\mathcal {S}))} \left| \frac{\nabla v}{v} - \frac{\nabla t}{t}\right| ^2 |t| \, dt\, dp, \quad v=u\circ \rho _{\mathcal {S}}. \end{aligned}$$

**Step 5: Conclusion on** $I_{\mathcal {S}}$ **using the flat case.** It is easy to see from the definition that Chord-Arc Domains are preserved by bi-Lipschitz change of variable, and the new CAD constants depends only on the old ones and the Lipschitz constants of the bi-Lipschitz map. Since the bi-Lipschitz constants of $\rho _{\mathcal {S}}$ is less than 2 (and so uniform in $\mathcal {S})$, we deduce that $\rho ^{-1}_{\mathcal {S}}(\Omega )$ is a Chord-Arc Domain with CAD constants that depends only on the CAD constants of $\Omega .$

Then, in Sect. 4, we constructed a cut-off function $\Psi _{\mathcal {S}}$ adapted to $W_{\Omega }(\mathcal {S}).$ We have shown in Lemma 4.20 that $\Psi _{\mathcal {S}}$ is 1 on $W_{\Omega }(\mathcal {S})$ and supported in $W^*_{\Omega }(\mathcal {S}),$ on which we still have $\delta (X) \approx {{\,\textrm{dist}\,}}(X,\Gamma _{\mathcal {S}}).$ In Lemma 4.28, we proved that the support of $\nabla \Psi _{\mathcal {S}}$ is small, in the sense that implies ${\mathbb {1}}_{{{\,\textrm{supp}\,}}\nabla \Psi _{\mathcal {S}}} \in CM_{\Omega }.$ What is important is that those two properties are preserved by bi-Lipschitz change of variable, and thus $\Psi _{\mathcal {S}} \circ \rho _{\mathcal {S}}$ is as in Definition 7.1.

We want the support of $\Psi _{\mathcal {S}} \circ \rho _{\mathcal {S}}$ to be included in a ball $B_{\mathcal {S}}$ such that $2B_{\mathcal {S}}$ is a subset of our initial ball *B*,  and such that the radius of $B_{\mathcal {S}}$ is smaller than $C\ell (Q(\mathcal {S})).$ But those facts are an easy consequence of (1.25) and the definition of $W^*_{\Omega }(\mathcal {S})$ (and the fact that $\rho _{\mathcal {S}}$ is bi-Lipschitz with the Lipschitz constant close to 1).

We also want $u\circ \rho _{\mathcal {S}}$ to be a solution of $L_{\mathcal {S}}(u\circ \rho _{\mathcal {S}}) = 0$ for a weak-DKP operator $L_{\mathcal {S}}.$ The operator $L_{\mathcal {S}}$ is not exactly weak-DKP everywhere in $\rho ^{-1}_{\mathcal {S}}(\Omega ),$ but it is the case on the support of $\Psi _{\mathcal {S}}$ (see Lemma 6.20), which is one condition that we need for applying Lemma 7.9.

To apply Lemma 7.9—or more precisely for Lemma 7.12, where one term from the integration by parts argument is treated—we need to show that $\omega _{L^*} \in A_{\infty }(\sigma ).$ This is a consequence of Theorem 1.18. Indeed, since the adjoint operator $L^*$ is also a weak DKP operator on $\Omega ,$ Theorem 1.18 asserts that $\omega _{L^*}\in A_{\infty }(\sigma ),$ where $\sigma$ is an Ahlfors regular measure on $\partial \Omega .$ A direct computation shows that for any set $E\subset \partial \Omega$ and any $X_0\in \Omega ,$

$$\begin{aligned} \omega _{L^*_{\mathcal {S}}}^{\rho _{\mathcal {S}}^{-1}(X_0)}\left( \rho _{\mathcal {S}}^{-1}(E)\right) =\omega _{L^*}^{X_0}(E), \end{aligned}$$

and since the mapping $\rho _{\mathcal {S}}$ is bi-Lipschitz, $\omega _{L^*}\in A_{\infty }(\sigma )$ implies that the $L^*_{\mathcal {S}}$-elliptic measure $\omega _{L^*_{\mathcal {S}}}\in A_{\infty }(\widetilde{\sigma }),$ where $\widetilde{\sigma }$ is an Ahlfors regular measure on the boundary of $\rho _{\mathcal {S}}^{-1}(\Omega ).$ Moreover, the intrinsic constants in $\omega _{L^*_{\mathcal {S}}}\in A_{\infty }(\widetilde{\sigma })$ depend only on the intrinsic constants in $\omega _{L^*}\in A_{\infty }(\sigma )$ because the bi-Lipschitz constants of $\rho _{\mathcal {S}}$ are bounded uniformly in $\mathcal {S}.$

All those verification made sure that we can apply Lemma 7.9, which entails that

1.32 $$\begin{aligned} I''_{\mathcal {S}}\le & {} \iint _{\rho ^{-1}(W_{\Omega }(\mathcal {S}))} |t| \left| \frac{\nabla (u\circ \rho _{\mathcal {S}})}{u\circ \rho _{\mathcal {S}}} - \frac{\nabla t}{t}\right| ^2 (\Psi _{\mathcal {S}} \circ \rho _{\mathcal {S}})^2 \, dt\, dp \nonumber \\\lesssim & {} \ell (Q(\mathcal {S}))^{n-1} \lesssim \sigma (Q(\mathcal {S})). \end{aligned}$$

Let us mention that the proof of Lemma 7.9 relies on an argument that was previously used—up to the authors’ knowledge—only in [25], which treats the cases where $\Omega$ is the complement of a low dimensional set. But even so, we also had to develop the technique presented in [32] to be able to treat the full class the weak-DKP operators.

**Step 6: Gathering of the estimates.** We let the reader check that (1.27)–(1.32) implies (1.24), and enjoy the end of the sketch of the proof!

### Organisation of the paper

In Sect. 2, we present the exact statement on our assumptions on $\Omega ,$ and we give the elliptic theory that will be needed in Sect. 7.

Sections 3–7 proved the implication $(\text {ii}) \implies (\text {iii})$ in Theorem 1.12. Section 3 introduces the reader to the uniform rectifiability and present the corona decomposition that will be needed. The corona decomposition gives a collection of (stopping time) coherent regimes $\{\mathcal {S}\}_{{\mathfrak {S}}}.$ From Sects. 3.4 to 6, $\mathcal {S}\in {\mathfrak {S}}$ is fixed. We construct in Sect. 3.4 a set $\Gamma _{\mathcal {S}}$ which is the graph of a Lipschitz function with small Lipschitz constant.

Section 4 associate a “Whitney” region $W_{\Omega }(\mathcal {S})$ to the coherent regime $\mathcal {S}$ so that from the stand point of each point of $W_{\Omega }(\mathcal {S}),$ $\Gamma _{\mathcal {S}}$ and $\partial \Omega$ are well approximated by the same planes.

In Sect. 5, we are applying the result from Sect. 4 to compare $D_{\beta }$ with the distance to a plane that approximate $\Gamma _{\mathcal {S}}.$

Section 6 constructs a bi-Lipschitz change of variable $\rho _{\mathcal {S}}$ that flattens $\Gamma _{\mathcal {S}},$ and we use the results from Sects. 4 and 5 in order to estimate the difference

$$\begin{aligned} \frac{\nabla [D_{\beta } \circ \rho _{\mathcal {S}}]}{D_{\beta } \circ \rho _{\mathcal {S}}} - \frac{\nabla t}{t} \end{aligned}$$

in terms of Carleson measure. Sections 3 to 6 are our arguments for the geometric side of the problem, in particular, the solutions to $Lu=0$ are barely mentioned (just to explain the effect of $\rho _{\mathcal {S}}$ on *L*).

Section 7 can be read independently and will contain our argument for the PDE side of the problem. Morally speaking, it proves Theorem 1.12$(\text {ii})\implies (\text {iii})$ when $\Omega = \mathbb {R}^n_+.$

Section 8 presents a sketch of proof of Theorem 1.21. The strategy is similar to our proof of Theorem 1.12, and in particular, many of the constructions and notations from Sects. 3 to 7 are adopted in Sect. 8. But since we do not need to deal with the regularized distance $D_{\beta },$ the proof is much shorter.

Section 9 tackles the converse implication, proving $(\text {v}) \implies (\text {i})$ in Theorem 1.12. The proof adapts an argument of [30], which states that if *G* is sufficiently close to $D_{\beta },$ then $\partial \Omega$ is uniformly rectifiable. As mentioned earlier, we unfortunately did not succeed to link our strong estimate (1.14) directly to the weak ones assumed in [30], which explains why we needed to rewrite the argument.

We finish with Sect. 10, where we construct a semi-uniform domain and a positive harmonic solution on it for which our estimate (1.13) is false.

## Miscellaneous

### Self improvement of the Carleson condition on $\mathcal {A}$

#### Lemma 8

Let $\mathcal {A}$ be a uniformly elliptic matrix on a domain $\Omega ,$ i.e. a matrix function that satisfies (1.6) and (1.7) with constant $C_\mathcal {A}.$ Assume that $\mathcal {A}$ can be decomposed as $\mathcal {A}= \mathcal {B}+ \mathcal {C}$ where

2.2 $$\begin{aligned} |\delta \nabla \mathcal {B}| + |\mathcal {C}| \in CM_{\Omega }(M). \end{aligned}$$

Then there exists another decomposition $\mathcal {A}= \widetilde{\mathcal {B}} + \widetilde{\mathcal {C}}$ such that

2.3 $$\begin{aligned} |\delta \nabla \widetilde{\mathcal {B}}| + |\widetilde{\mathcal {C}}| \in CM_{\Omega }(CM) \end{aligned}$$

with a constant $C>0$ that depends only on *n*,  and $\widetilde{\mathcal {B}}$ satisfies (1.6) and (1.7) with the same constant $C_{\mathcal {A}}$ as $\mathcal {A}.$ In addition, 

2.4 $$\begin{aligned} |\delta \nabla \widetilde{\mathcal {B}}| \le CC_{\mathcal {A}}. \end{aligned}$$

#### Proof

Let $\mathcal {A}= \mathcal {B}+ \mathcal {C}$ as in the assumption of the lemma. Let $\theta \in C^\infty _0(\mathbb {R}^n)$ be a nonnegative function such that ${{\,\textrm{supp}\,}}\theta \subset B(0,\frac{1}{10}),$ $\iint _{\mathbb {R}^n} \theta (X) dX = 1.$ Construct $\theta _X(Y) := \delta (X)^{-n} \theta \big (\frac{Y-X}{\delta (X)}\big )$ and then

2.5 $$\begin{aligned} \widetilde{\mathcal {B}}(X) := \iint _{\mathbb {R}^n} \mathcal {A}(Y) \, \theta _X(Y) \, dY \quad \text {and} \quad \widetilde{\mathcal {C}} := \mathcal {A}- \widetilde{\mathcal {B}}. \end{aligned}$$

We see that $\widetilde{\mathcal {B}}$ is an average of $\mathcal {A},$ so $\widetilde{B}$ verifies (1.6) and (1.7) with the same constant as $\mathcal {A}.$ So it remains to prove (2.3) and (2.4). Observe that

$$\begin{aligned} \nabla _X \theta _X(Y)= & {} - n \delta (X)^{-n-1} \nabla \delta (X) \theta \Bigg (\frac{Y-X}{\delta (X)}\Bigg ) - \delta (X)^{-n-1} (\nabla \theta )\Bigg (\frac{Y-X}{\delta (X)}\Bigg ) \\{} & {} - \delta (X)^{-n-2} \nabla \delta (X) (Y-X) \cdot (\nabla \theta )\Bigg (\frac{Y-X}{\delta (X)}\Bigg ). \end{aligned}$$

Let $\Theta (Z)$ denote $Z\theta (Z),$ then $\mathop {{\text {div}}}\Theta (Z)=n\theta (Z)+Z\cdot \nabla \theta (Z).$ So

$$\begin{aligned} \delta (X) \nabla _X \theta _X(Y) = - \delta (X)^{-n} (\nabla \theta )\Bigg (\frac{Y-X}{\delta (X)}\Bigg ) - \delta (X)^{-n} \nabla \delta (X) (\mathop {{\text {div}}}\Theta )\Bigg (\frac{Y-X}{\delta (X)}\Bigg ). \end{aligned}$$

From here, one easily sees that $\left| \delta (X) \nabla _X \theta _X(Y)\right|$ is bounded by $C\delta (X)^{-n}$ uniformly in *X* and *Y*,  and thuswhich proves (2.4). Set $\Theta _X(Y) = \delta (X)^{-n} \Theta \Big (\frac{Y-X}{\delta (X)}\Big ) .$ Then

$$\begin{aligned} \delta (X) \nabla _X \theta _X(Y) = - \delta (X) \nabla _Y \theta _X(Y) - \delta (X) \nabla \delta (X){{\,\textrm{div}\,}}_Y\Theta _X(Y). \end{aligned}$$

As a consequence,

$$\begin{aligned} \delta (X) \nabla \widetilde{\mathcal {B}}(X)= & {} \iint _{\mathbb {R}^n} (\mathcal {B}+ \mathcal {C})(Y) \, \delta (X) \nabla _X \theta _X(Y) \, dY \\= & {} \delta (X) \iint _{\mathbb {R}^n} \nabla \mathcal {B}(Y) \, \theta _X(Y) \, dY + \delta (X) \nabla \delta (X) \iint _{\mathbb {R}^n} \nabla \mathcal {B}(Y) \cdot \Theta _X(Y) \, dY \\{} & {} + \iint _{\mathbb {R}^n} \mathcal {C}(Y) \, [\delta (X) \nabla _X \theta _X(Y)] \, dY. \end{aligned}$$

We deduce thatand so the fact that $|\delta \nabla \mathcal {B}| + |\mathcal {C}| \in CM_{\Omega }(M)$ is transmitted to $\delta \nabla \widetilde{\mathcal {B}},$ i.e. $\delta \nabla \widetilde{\mathcal {B}} \in CM_{\Omega }(CM).$

As for $\widetilde{\mathcal {C}},$ since $\iint \theta _X(Y) dY = 1,$ we have2.6By Fubini’s theorem, to show that $\left| \widetilde{\mathcal {C}}\right| \in CM_{\Omega }(CM),$ it suffices to show that for any ball *B* centered on the boundary,From this one sees that the terms on the right-hand side of (2.6) that involves $\mathcal {C}$ can be easily controlled using $\left| \mathcal {C}\right| \in CM_\Omega (M).$ So by the Cauchy–Schwarz inequality, it suffices to control2.7Notice that for all $X\in B(Z,\delta (Z)/4),$ $B(X,\delta (X)/10)\subset B(Z,\delta (Z)/2),$ and thusTherefore,by the Poincaré inequality, Fubini’s theorem, and $\left| \delta \nabla \mathcal {B}\right| \in CM_\Omega (M).$ So again, the Carleson bound on $|\delta \nabla \mathcal {B}| + |\mathcal {C}|$ is given to $\widetilde{\mathcal {C}}$ as well. The lemma follows. $\square$

### Definition of chord-arc domains

#### Definition 9

(*Corkscrew condition*, [42]) We say that a domain $\Omega \subset \mathbb R^n$ satisfies the * corkscrew condition* with constant $c\in (0,1)$ if for every surface ball $\Delta :=\Delta (x,r),$ with $x\in \partial \Omega$ and $0<r<{{\,\textrm{diam}\,}}(\Omega ),$ there is a ball $B(X_\Delta ,cr)\subset B(x,r)\cap \Omega .$ The point $X_\Delta \subset \Omega$ is called a **corkscrew point** relative to $\Delta$ (or, for *x* at scale *r*).

#### Definition 10

(*Harnack Chain condition*, [42]) We say that $\Omega$ satisfies the *Harnack Chain condition* with constants *M*,  $C>1$ if for every $\rho >0,\, \Lambda \ge 1,$ and every pair of points $X,X' \in \Omega$ with $\delta (X),\,\delta (X') \ge \rho$ and $|X-X'|<\Lambda \,\rho ,$ there is a chain of open balls $B_1,\dots ,B_N \subset \Omega ,$ $N\le M(1+\log \Lambda ),$ with $X\in B_1,\, X'\in B_N,$ $B_k\cap B_{k+1}\ne \emptyset$ and $C^{-1}{{\,\textrm{diam}\,}}(B_k) \le {{\,\textrm{dist}\,}}(B_k,\partial \Omega )\le C{{\,\textrm{diam}\,}}(B_k).$ The chain of balls is called a *Harnack Chain*.

#### Definition 11

(1-*Sided NTA and NTA*) We say that a domain $\Omega$ is a *1-sided NTA domain* with constants *c*, *C*, *M* if it satisfies the corkscrew condition with constant *c* and Harnack Chain condition with constant *M*, *C*. Furthermore, we say that $\Omega$ is an *NTA domain* if it is a 1-sided NTA domain and if, in addition, $\Omega _{\text {ext}}:= \mathbb R^n{\setminus } {\overline{\Omega }}$ also satisfies the corkscrew condition.

#### Definition 12

(1-*Sided CAD and CAD*) A *1-sided chord-arc domain* (1-sided CAD) is a 1-sided NTA domain with AR boundary. The 1-sided NTA constants and the AR constant are called the 1-sided CAD constants. A *chord-arc domain* (CAD, or 2-sided CAD) is an NTA domain with AR boundary. The 1-sided NTA constants, the corkscrew constant for $\Omega _{\text {ext}},$ and the AR constant are called the CAD constants.

Uniform rectifiability (UR) is a quantitative version of rectifiability.

#### Definition 13

(*UR*) We say that *E* is uniformly rectifiable if *E* has big pieces of Lipschitz images, that is, if *E* is $(n-1)$-Ahlfors regular (1.1), and there exist $\theta , M>0$ such that, for each $x\in E$ and $r>0,$ there is a Lipschitz mapping $\rho$ from the ball $B(0,r) \subset \mathbb {R}^d$ into $\mathbb {R}^n$ such that $\rho$ has Lipschitz norm $\le M$ and

$$\begin{aligned} \sigma (E \cap B(x,r) \cap \rho (B_{\mathbb {R}^d}(0,r))) \ge \theta r^d. \end{aligned}$$

However, we shall not use the above definition. What we do require is the characterization of UR by Tolsa’s $\alpha$-numbers [50], as well as a modification of the corona decomposition of uniformly rectifiable sets constructed in [18]. See Sect. 3 for details. We shall also need the following result.

#### Lemma 14

Suppose that $\Omega \subset \mathbb R^n$ is 1-sided chord-arc domain. Then the following are equivalent : 

1. $\partial \Omega$ is uniformly rectifiable.
2. $\Omega _{\text {ext}}$ satisfies the corkscrew condition,  and hence,  $\Omega$ is a chord-arc domain.

That (1) implies (2) was proved in [5]. That (2) implies (1) can be proved via the $A_{\infty }$ of harmonic measure (see [5, Theorem 1.2]), or directly as in [17].

### Preliminary PDE estimates

#### Lemma 15

(The Caccioppoli inequality) Let $L=-{{\,\textrm{div}\,}}A\nabla$ be a uniformly elliptic operator and $u\in W^{1,2}(2B)$ be a solution of $Lu=0$ in 2*B*,  where *B* is a ball with radius *r*. Then there exists *C* depending only on *n* and the ellipticity constant of *L* such that

#### Lemma 16

(The Harnack inequality) Let *L* be as in Lemma 2.14 and let *u* be a nonnegative solution of $Lu=0$ in $2B\subset \Omega .$ Then there exists constant $C\ge 1$ depending only on *n* and the ellipticity constant of *L* such that

$$\begin{aligned} \sup _B u \le C\inf _B u. \end{aligned}$$

Write $L^*$ for the transpose of *L* defined by $L^*=-{{\,\textrm{div}\,}}A^\top \nabla ,$ where $A^\top$ denotes the transpose matrix of *A*. Associated with *L* and $L^*$ one can respectively construct the elliptic measures $\{\omega _L^X\}_{X\in \Omega }$ and $\{\omega _{L^*}^X\}_{X\in \Omega },$ and the Green functions $G_L$ and $G_{L^*}$ on domains with Ahlfors regular boundaries (cf. [40, 43]).

#### Lemma 17

(The Green function) Suppose that $\Omega \subset \mathbb {R}^{n}$ is an open set such that $\partial \Omega$ is Ahlfors regular. Given an elliptic operator *L*,  there exists a unique Green function $G_L(X,Y): \Omega \times \Omega {\setminus } {{\,\textrm{diag}\,}}(\Omega ) \rightarrow \mathbb {R}$ with the following properties :  $G_L(\cdot ,Y)\in W^{1,2}_{\text {loc}}(\Omega {\setminus } \{Y\})\cap C({\overline{\Omega }}{\setminus }\{Y\}),$ $G_L(\cdot ,Y)\big |_{\partial \Omega }\equiv 0$ for any $Y\in \Omega ,$ and $L G_L(\cdot ,Y)=\delta _Y$ in the weak sense in $\Omega ,$ that is, 

$$\begin{aligned} \iint _{\Omega } A(X)\,\nabla _X G_{L}(X,Y) \cdot \nabla \varphi (X)\, dX=\varphi (Y), \quad \text {for any }\varphi \in C_c^\infty (\Omega ). \end{aligned}$$

In particular,  $G_L(\cdot ,Y)$ is a weak solution to $L G_L(\cdot ,Y)=0$ in $\Omega {\setminus }\{Y\}.$ Moreover, 

2.17 $$\begin{aligned}{} & {} G_L(X,Y) \le C\,|X-Y|^{2-n}\quad \text {for }X,Y\in \Omega ,\nonumber \\{} & {} c_\theta \,|X-Y|^{2-n}\le G_L(X,Y),\quad \text {if } |X-Y|\le \theta \, {{\,\textrm{dist}\,}}(X,\partial \Omega ),\ \theta \in (0,1),\nonumber \\{} & {} G_L(X,Y)\ge 0,\quad G_L(X,Y)=G_{L^*}(Y,X), \qquad \text {for all } X,Y\in \Omega ,\, X\ne Y. \end{aligned}$$

The following lemma will be referred to as the CFMS estimates (cf. [11, 43] for NTA domains, and [41] or [24] for 1-sided CAD).

#### Lemma 18

(The CFMS estimates) Let $\Omega$ be a 1-sided CAD domain. Let *L* be an elliptic operator satisfying (1.6) and (1.7). There exist *C* depending only on *n*,  $C_\mathcal {A},$ and the 1-sided CAD constants,  such that for any $B:=B(x,r),$ with $x\in \partial \Omega ,$ $0<r<{{\,\textrm{diam}\,}}(\partial \Omega )$ and $\Delta :=\Delta (x,r),$ we have the following properties.

1. The elliptic measure is non-degenerate,  that is
  $$\begin{aligned} C^{-1} \le \omega _L^{X_\Delta }(\Delta ) \le C. \end{aligned}$$
2. For $X\in \Omega {\setminus } 2\,B$ we have
  2.19 $$\begin{aligned} \frac{1}{C}\omega _L^X(\Delta ) \le r^{n-2}G_L(X,X_\Delta ) \le C \omega _L^X(\Delta ). \end{aligned}$$
3. If $0\le u, v\in W^{1,2}_{\text {loc}}(4\,B\cap \Omega )\cap C(\overline{4\,B\cap \Omega })$ are two nontrivial weak solutions of $Lu=Lv=0$ in $4\,B\cap \Omega$ such that $u=v=0$ in $4\,\Delta ,$ then
  $$\begin{aligned} C^{-1}\frac{u(X_\Delta )}{v(X_\Delta )}\le \frac{u(X)}{v(X)}\le C\frac{u(X_\Delta )}{v(X_\Delta )},\quad \text {for all }X\in B\cap \Omega . \end{aligned}$$

## Characterization of the uniform rectifiability

In all this section, we assume that $\partial \Omega$ is uniformly rectifiable, and we plan to prove a corona decomposition of the uniformly rectifiable set which is “Tolsa’s $\alpha$-number compatible”.

Instead of a long explanation of the section, which will not be helpful anyway to any reader who is not already fully familiar with the corona decomposition (C3) in [18] and the Tolsa $\alpha$-number (see [50]), we shall only state below the results proved in the section (the definition of all the notions and notation will be ultimately given in the section below).

### Lemma 19

Let $\partial \Omega$ be a uniformly rectifiable set. Given any positive constants $0<\epsilon _1< \epsilon _0 < 1,$ there exists a disjoint decomposition ${\mathbb {D}}_{\partial \Omega } = {\mathcal {G}}\cup \mathcal {B}$ such that

(i) The “good” cubes $Q\in {\mathcal {G}}$ are such that $\alpha _{\sigma }(Q) \le \epsilon _1$ and
  3.2 $$\begin{aligned} \sup _{y \in 999\Delta _Q} {{\,\textrm{dist}\,}}(y,P_Q) + \sup _{p\in P_Q \cap 999B_Q} {{\,\textrm{dist}\,}}(p,\partial \Omega ) \le \epsilon _1 \ell (Q). \end{aligned}$$
(ii) The collection ${\mathcal {G}}$ of “good” cubes can be further subdivided into a disjoint family ${\mathcal {G}}= \displaystyle \bigcup \nolimits _{\mathcal {S}\in {\mathfrak {S}}} \mathcal {S}$ of **coherent** regimes such that for any $\mathcal {S}\in {\mathfrak {S}},$ there exists a hyperplane $P:=P_{\mathcal {S}}$ and a $2\epsilon _0$-Lipschitz function ${\mathfrak {b}}_{\mathcal {S}}:= {\mathfrak {b}}$ on *P* such that
  3.3 $$\begin{aligned} \int _{P\cap \Pi (2B_Q)} {{\,\textrm{dist}\,}}({\mathfrak {b}}(p),P_Q) \, dp \le C \ell (Q) \sigma (Q) \alpha _{\sigma }(Q) \quad \text {for } Q\in \mathcal {S}, \end{aligned}$$
  where *C* depends only on *n*.
(iii) The cubes in $\mathcal {B}$ (the “bad” cubes) and the maximal cubes $Q(\mathcal {S})$ satisfies the Carleson packing condition
  3.4 $$\begin{aligned} \sum _{{\begin{array}{c} Q\in \mathcal {B}\\ Q \subset Q_0 \end{array}}} \sigma (Q) + \sum _{{\begin{array}{c} \mathcal {S}\in {\mathfrak {S}} \\ Q(\mathcal {S}) \subset Q_0 \end{array}}} \sigma (Q(\mathcal {S})) \le C_{\epsilon _0,\epsilon _1} \sigma (Q_0) \quad \text {for all } Q_0\in {\mathbb {D}}_{\partial \Omega }. \end{aligned}$$

In the above lemma, $\sigma$ is the Ahlfors regular measure for $\partial \Omega$ given in (1.1), ${\mathbb {D}}_{\partial \Omega }$ is a dyadic decomposition of $\partial \Omega ,$ $\Pi :=\Pi _{\mathcal {S}}$ is the orthogonal projection on $P_{\mathcal {S}},$ $P_Q$ is the best approximating plane of $\partial \Omega$ around *Q*,  and $\alpha _{\sigma }$ is the Tolsa $\alpha$-number for $\sigma .$ The novelty, which is not similar to any of the corona decompositions that the authors are aware of, is (3.3), which quantify the difference between $\partial \Omega$ and the approximating graph $\Gamma _{\mathcal {S}}$ in terms of $\alpha$-number. In a more “classical” corona decomposition, one would have $\epsilon _1$ instead of $\alpha _{\sigma }(Q)$ in (3.3).

Corona decompositions are a useful and popular tool in the recent literature pertaining to uniformly rectifiable sets, see for instance [3, 4, 8, 9, 12, 18, 33, 36, 46, 47] to cite only a few.

### Dyadic decomposition

We construct a dyadic system of pseudo-cubes on $\partial \Omega .$ In the presence of the Ahlfors regularity property, such construction appeared for instance in [15, 16, 18] or [19]. We shall use the very nice construction of Christ [14], that allow to bypass the need of a measure on $\partial \Omega .$ More exactly, one can check that the construction of the dyadic sets by Christ to not require a measure, and as such are independent on the measure on $\partial \Omega .$

There exist a universal constant $0<a_0<1$ and a collection ${\mathbb {D}}_{\partial \Omega } = \cup _{k \in {{\mathbb {Z}}}} {\mathbb {D}}_{k}$ of Borel subsets of $\partial \Omega ,$ with the following properties. We write

$$\begin{aligned} {\mathbb {D}}_{k}:=\{Q_{j}^k\subset {\mathbb {D}}_{\partial \Omega }: j\in {\mathfrak {I}}_k\}, \end{aligned}$$

where ${\mathfrak {I}}_k$ denotes some index set depending on *k*,  but sometimes, to lighten the notation, we shall forget about the indices and just write $Q \in {\mathbb {D}}_k$ and refer to *Q* as a cube (or pseudo-cube) of generation *k*. Such cubes enjoy the following properties:

(i) $$\partial \Omega =\cup _{j} Q_{j}^k \,\,$$ for any $$k \in {{\mathbb {Z}}}.$$
(ii) If $m > k$ then either $Q_{i}^{m}\subseteq Q_{j}^{k}$ or $Q_{i}^{m}\cap Q_{j}^{k}=\emptyset .$
(iii) $$Q_i^m \cap Q_j^m=\emptyset $$ if $$i\ne j.$$
(iv) Each pseudo-cube $Q\in {\mathbb {D}}_k$ has a “center” $x_Q\in \partial \Omega$ such that
  3.5 $$\begin{aligned} \Delta (x_Q,a_02^{-k})\subset Q \subset \Delta (x_Q,2^{-k}). \end{aligned}$$

Let us make a few comments about these cubes. We decided to use a dyadic scaling (by opposition to a scaling where the ratio of the sizes between a pseudo-cube and its parent is, in average, $\epsilon < \frac{1}{2})$ because it is convenient. The price to pay for forcing a dyadic scaling is that if $Q \in {\mathbb {D}}_{k+\ell }$ and *R* is the cube of ${\mathbb {D}}_k$ that contains *Q* (it is unique by (*ii*), and it is called an ancestor of *Q*) is *not* necessarily strictly larger (as a set) than *Q*. We also considered that the $\partial \Omega$ was unbounded, to avoid separating cases. If the boundary $\partial \Omega$ is bounded, then ${\mathbb {D}}_{\partial \Omega } := \bigcup _{k\le k_0} {\mathbb {D}}_k$ where $k_0$ is such that $2^{k_0-1} \le {{\,\textrm{diam}\,}}(\Omega ) \le 2^{k_0-1},$ and we let the reader check that this variation doesn’t change a single argument in the sequel.

If $\mu$ is any doubling measure on $\partial \Omega$—that is if $\mu (2\Delta ) \le C_\mu \mu (\Delta )$ for any boundary ball $\Delta \subset \partial \Omega$—then we have the following extra property:

(v) $$\mu (\partial Q_i^k) = 0$$ for all *i*, *k*.

In our construction, (i) and (iii) forces the $Q_i^k$ to be neither open nor closed. But this last property (v) means that taking the relative interior or the closure of $Q_i^k$ instead of $Q_i^k$ would not matter, since the boundary amounts to nothing.

Let us introduce some extra notation. When $E \subset \partial \Omega$ is a set, ${\mathbb {D}}_{\partial \Omega }(E)$ is the sub-collection of dyadic cubes that are contained in *E*. When $Q\in {\mathbb {D}}_{\partial \Omega },$ we write *k*(*Q*) for the generation of *Q* and $\ell (Q)$ for $2^{-k(Q)},$ which is roughly the diameter of *Q* by (3.5). We also use $B_Q \subset \mathbb {R}^n$ for the ball $B(x_Q,\ell (Q))$ and $\Delta _Q$ for the boundary ball $\Delta (x_Q,\ell (Q))$ that appears in (3.5). For $\kappa \ge 1,$ the dilatation $\kappa Q$ is

3.6 $$\begin{aligned} \kappa Q = \{x\in \partial \Omega , \, {{\,\textrm{dist}\,}}(x,Q) \le (\kappa - 1) \ell (Q)\}, \end{aligned}$$

which means that $\kappa Q \subset \kappa \Delta _Q \subset (\kappa +1) Q.$

The dyadic decomposition of $\partial \Omega$ will be the one which is the most used. However, we also use dyadic cubes for other sets, for instance to construct Whitney regions, and we use the same construction and notation as the one for $\partial \Omega .$ In particular, we will use dyadic cubes in $\mathbb {R}^n$ and in a hyperplane *P* that still satisfy (3.5) for the universal constant $a_0$—i.e. the dyadic cubes are not real cubes—and the definition (3.6) holds even in those contexts.

### Tolsa’s $\alpha$ numbers

Tolsa’s $\alpha$ numbers estimate how far a measure is from a flat measure, using Wasserstein distances. We denote by $\Xi$ the set of affine $n-1$ planes in $\mathbb {R}^n,$ and for each plane $P\in \Xi ,$ we write $\mu _P$ for the restriction to *P* of the $(n-1)$-dimensional Hausdorff measure, that is $\mu _P$ is the Lebesgue measure on *P*. A flat measure is a measure $\mu$ that can be written $\mu = c\mu _P$ where $c>0$ and $P \in \Xi ,$ the set of flat measure is then denoted by ${\mathcal {F}}.$

#### Definition 20

(*Local Wasserstein distance*) If $\mu$ and $\sigma$ are two $(n-1)$-Ahlfors regular measures on $\mathbb {R}^n,$ and if $y \in \mathbb {R}^n$ and $s>0,$ we define

$$\begin{aligned} {{\,\textrm{dist}\,}}_{y,s}(\mu ,\sigma ) := s^{-n} \sup _{f\in Lip(y,s)} \left| \int f\, d\mu - \int f \, d\sigma \right| \end{aligned}$$

where *Lip*(*y*, *s*) is the set of 1-Lipschitz functions that are supported in *B*(*y*, *s*).

If $Q \in {\mathbb {D}}_{\partial \Omega },$ then we set ${{\,\textrm{dist}\,}}_{Q}(\mu ,\sigma ) := {{\,\textrm{dist}\,}}_{x_Q,10^3\ell (Q)}(\mu ,\sigma )$ and $Lip(Q) := Lip(x_Q,10^3\ell (Q)),$ where $x_Q$ is as in (3.5). Moreover, if $\sigma$ is an Ahlfors regular measure on $\partial \Omega ,$ then we set

$$\begin{aligned} \alpha _{\sigma }(Q) := \inf _{\mu \in {\mathcal {F}}} {{\,\textrm{dist}\,}}_Q(\mu ,\sigma ). \end{aligned}$$

Note that

3.8 $$\begin{aligned} 0\le \alpha _{\sigma }(Q)\le C \quad \text {for all }Q\in {\mathbb {D}}_{\partial \Omega } \end{aligned}$$

where $C<\infty$ depends only on the Ahlfors constants of $\mu$ and $\sigma .$

The uniform rectifiability of $\partial \Omega$ is characterized by the fact that, for any $(n-1)$-Ahlfors regular measure $\sigma$ supported on $\partial \Omega ,$ and any $Q_0\in {\mathbb {D}}_{\partial \Omega },$ we have

3.9 $$\begin{aligned} \sum _{Q\in {\mathbb {D}}_{\partial \Omega }(Q_0)} \alpha _{\sigma }(Q)^2 \sigma (Q) \le C \sigma (Q_0) \approx \ell (Q_0)^{n-1} \end{aligned}$$

and, for any $\epsilon >0,$

3.10 $$\begin{aligned} \sum _{{\begin{array}{c} Q\in {\mathbb {D}}_{\partial \Omega }(Q_0)\\ \alpha _{\sigma }(Q) > \epsilon \end{array}}} \sigma (Q) \le C_\epsilon \sigma (Q_0) \approx \ell (Q_0)^{n-1}. \end{aligned}$$

For a proof of these results, see Theorem 1.2 in [50].

It will be convenient to introduce the following notation. Given $Q\in {\mathbb {D}}_{\partial \Omega },$ the quantities $c_Q,$ $P_Q,$ and $\mu _Q$ are such that

3.11 $$\begin{aligned} \mu _Q = c_Q \mu _{P_Q} \quad \text {and} \quad {{\,\textrm{dist}\,}}_Q(\sigma ,\mu _Q) \le 2 \alpha _{\sigma }(Q), \end{aligned}$$

that is $\mu _Q$ is a flat measure which well approximates $\sigma$ (as long as $\alpha _{\sigma }(Q)$ is small). So it means that

3.12 $$\begin{aligned} \left| \int f\, d\sigma - \int f \, d\mu _Q \right| \le 2(10^3\ell (Q))^{n} \alpha _{\sigma }(Q) \quad \text {for } f\in Lip(Q). \end{aligned}$$

Let us finish the subsection with the following simple result.

#### Lemma 21

There exists $C>1$ depending only on $C_{\sigma }$ and *n* such that if $Q \in {\mathbb {D}}_{\partial \Omega }$ and $\epsilon \in (0,C^{-n})$ verify $\alpha _{\sigma }(Q) \le \epsilon ,$ then

3.14 $$\begin{aligned} \sup _{y \in 999\Delta _Q} {{\,\textrm{dist}\,}}(y,P_Q) + \sup _{p\in P_Q \cap 999B_Q} {{\,\textrm{dist}\,}}(p,\partial \Omega ) \le C \epsilon ^{1/n} \ell (Q). \end{aligned}$$

#### Proof

Assume that $\alpha _{\sigma }(Q) \le \epsilon = 8000^{-n} C_{\sigma }^{-1} \eta ^{n}$ with $\eta \in (0,1).$ For a given point $y\in 999\Delta _Q,$ we set the function $f_1(z):= \max \{0,\eta \ell (Q) - |y-z|\} \in Lip(Q).$ Observe that

$$\begin{aligned} \int f_1 d\sigma \ge \frac{\eta \ell (Q)}{2} \sigma \left( \frac{\eta }{2} \Delta (y,\ell (Q))\right) \ge C_{\sigma }^{-1} \Big (\frac{\eta \ell (Q)}{2}\Big )^n. \end{aligned}$$

and thanks to (3.12)

$$\begin{aligned} 8000^{-n} C_{\sigma }^{-1} \eta ^{n} > \alpha _{\sigma }(Q) \ge (2000\ell (Q))^{-n} \left| \int f_1 \, d\sigma - \int f_1 \, d\mu _Q \right| . \end{aligned}$$

By combining the two inequalities above, we have

$$\begin{aligned} \left| \int f_1 \, d\sigma - \int f_1 \, d\mu _Q \right| \le C_{\sigma }^{-1} \Big (\frac{\eta \ell (Q)}{4}\Big )^n \le \frac{1}{2} \int f_1 d\sigma . \end{aligned}$$

So necessarily, the support of $f_1$ intersects the support of $\mu _Q,$ that is, we have that ${{\,\textrm{dist}\,}}(y,P_Q) \le \eta \ell (Q)$ and thus the first part of (3.14) is proved. But notice also that the same computations force the constant $c_Q$ in the flat measure $\mu _Q = c_Q \mu _{P_Q}$ to be larger than $(2c_{n-1}C_{\sigma })^{-1},$ where $c_n$ is the volume of the *n*-dimensional unit ball. We take now a point $p\in P_Q \cap 999B_Q$ and construct $f_2:= \max \{0,\eta \ell (Q) - |p-z|\} \in Lip(Q).$ We have

$$\begin{aligned}\begin{aligned} \left| \int f_2 \, d\sigma - \int f_2 \, d\mu _Q \right| \le 2(1000\ell (Q))^n \alpha _{\sigma }(Q)< C_{\sigma }^{-1} \Big (\frac{\eta \ell (Q)}{4}\Big )^n < \int f_2 \, d\mu _Q. \end{aligned}\end{aligned}$$

So necessarily, the support of $f_1$ intersects the support of $\sigma ,$ that is ${{\,\textrm{dist}\,}}(p,\partial \Omega ) \le \eta \ell (Q).$ The lemma follows. $\square$

### Corona decomposition

We first introduce the notion of coherent subsets of ${\mathbb {D}}_{\partial \Omega }.$

#### Definition 22

Let ${\mathcal {S}} \subset {\mathbb {D}}_{\partial \Omega }.$ We say that ${\mathcal {S}}$ is **coherent** if

- $\mathcal {S}$ contains a unique maximal element $Q({\mathcal {S}}),$ that is $Q({\mathcal {S}})$ contains all the other elements of $\mathcal {S}$ as subsets.
- If $Q\in \mathcal {S}$ and $Q \subset R \subset Q(\mathcal {S}),$ then $R\in \mathcal {S}.$
- Given a cube $Q \in \mathcal {S},$ either all its children belong to $\mathcal {S}$ or none of them do.

The aim of the section is to prove the following corona decomposition for a uniformly rectifiable boundary $\partial \Omega .$

#### Lemma 23

Let $\partial \Omega$ be a uniformly rectifiable set. Given any positive constants $\epsilon _1 < \epsilon _0 \in (0,1),$ there exists a disjoint decomposition ${\mathbb {D}}_{\partial \Omega } = {\mathcal {G}}\cup \mathcal {B}$ such that

(i) The “good” cubes $Q\in {\mathcal {G}}$ are such that $\alpha _{\sigma }(Q) \le \epsilon _1$ and
  3.17 $$\begin{aligned} \sup _{y \in 999\Delta _Q} {{\,\textrm{dist}\,}}(y,P_Q) + \sup _{p\in P_Q \cap 999B_Q} {{\,\textrm{dist}\,}}(p,\partial \Omega ) \le \epsilon _1 \ell (Q). \end{aligned}$$
(ii) The collection ${\mathcal {G}}$ of “good” cubes can be further subdivided into a disjoint family ${\mathcal {G}}= \displaystyle \bigcup \nolimits _{\mathcal {S}\in {\mathfrak {S}}} \mathcal {S}$ of **coherent** regimes that satisfy
  3.18 $$\begin{aligned} {{\,\textrm{Angle}\,}}(P_Q,P_{Q'}) \le \epsilon _0 \quad \text {for all } \mathcal {S}\in {\mathfrak {S}} \text { and } Q,Q'\in \mathcal {S}. \end{aligned}$$
(iii) The cubes in $\mathcal {B}$ (the “bad” cubes) and the maximal cubes $Q(\mathcal {S})$ satisfy the Carleson packing condition
  3.19 $$\begin{aligned} \sum _{{\begin{array}{c} Q\in \mathcal {B}\\ Q \subset Q_0 \end{array}}} \sigma (Q) + \sum _{{\begin{array}{c} \mathcal {S}\in {\mathfrak {S}} \\ Q(\mathcal {S}) \subset Q_0 \end{array}}} \sigma (Q(\mathcal {S})) \le C_{\epsilon _0,\epsilon _1} \sigma (Q_0) \quad \text {for all } Q_0\in {\mathbb {D}}_{\partial \Omega }. \nonumber \\ \end{aligned}$$

#### Remark 3.20

What we secretly expect is, in addition to (3.18), to also have a control on the constants $c_Q$—defined in (3.11)—that belongs to the same $\mathcal {S}.$ For instance, we would like to have

$$\begin{aligned} |c_Q-c_{Q(\mathcal {S})}| \le \epsilon _0. \end{aligned}$$

Imposing this extra condition while keeping the number of $\mathcal {S}$ low should be doable, but we do not need it, so we avoided this complication.

The difficult part in the above lemma is to prove that (3.18) holds while keeping the number of coherent regimes $\mathcal {S}$ small enough so that (3.19) stays true. To avoid a long and painful proof, we shall prove Lemma 3.16 with the following result as a startpoint.

#### Lemma 25

[18] Let $\partial \Omega$ be a uniformly rectifiable set. Given any positive constants $\epsilon _3 < \epsilon _2 \in (0,1),$ there exists a disjoint decomposition ${\mathbb {D}}_{\partial \Omega } = {\mathcal {G}}' \cup \mathcal {B}'$ such that

(i) The “good” cubes $Q\in {\mathcal {G}}'$ are such that there exists an affine plane $P'_Q \in \Xi$ such that
  3.22 $$\begin{aligned} {{\,\textrm{dist}\,}}(x,P'_Q) \le \epsilon _3 \ell (Q) \quad \text {for } x\in 999\Delta _Q. \end{aligned}$$
(ii) The collection ${\mathcal {G}}'$ of “good” cubes can further subdivided into a disjoint family ${\mathcal {G}}' = \displaystyle \bigcup \nolimits _{\mathcal {S}' \in {\mathfrak {S}}'} \mathcal {S}'$ of **coherent** stopping time regimes that satisfy
  3.23 $$\begin{aligned} {{\,\textrm{Angle}\,}}(P'_Q,P'_{Q(\mathcal {S}')}) \le \epsilon _2 \quad \text {for all } \mathcal {S}'\in {\mathfrak {S}}' \text { and } Q\in \mathcal {S}'. \end{aligned}$$
(iii) The cubes in $\mathcal {B}'$ and the maximal cubes $Q(\mathcal {S}')$ satisfy the Carleson packing condition
  3.24 $$\begin{aligned} \sum _{{\begin{array}{c} Q\in \mathcal {B}' \\ Q \subset Q_0 \end{array}}} \sigma (Q) + \sum _{{\begin{array}{c}\mathcal {S}' \in {\mathfrak {S}}' \\ Q(\mathcal {S}') \subset Q_0 \end{array}}} \sigma (Q(\mathcal {S}')) \le C_{\epsilon _2,\epsilon _3} \sigma (Q_0) \quad \text {for all } Q_0\in {\mathbb {D}}_{\partial \Omega }. \end{aligned}$$

The proof of Lemma 3.21 is contained in Sections 6 to 11 of [18], and the statement that we gave is the combination of Lemma 7.1 and Lemma 7.4 in [18]. Lemma 3.16 might already be stated and proved in another article, and we apologize if it were the case. Moreover, the proof of Lemma 3.16 is probably obvious to anyone that is a bit familiar with this tool. However, every corona decomposition has its own small differences, and we decided to write our own using only the results of David and Semmes as a prerequisite.

#### Proof of Lemma 3.16 from Lemma 3.21

We pick then $\epsilon _1$ and $\epsilon _0$ small such that $\epsilon _1\ll \epsilon _0 \ll 1.$ We apply Lemma 3.21 with the choices of $\epsilon _2:= \epsilon _0/2$ and $\epsilon _3= \epsilon _1.$ Note that we can choose

3.25 $$\begin{aligned} P'_Q = P_Q \quad \text {when } Q \in {\mathcal {G}}' \text { and } \alpha _{\sigma }(Q) \le C^{-n} \epsilon _1^{n} \end{aligned}$$

if $C>0$ is the constant from Lemma 3.13.

Since we applied Lemma 3.21, we have a first disjoint decomposition ${\mathbb {D}}_{\partial \Omega } = {\mathcal {G}}' \cup \mathcal {B}'$ and a second decomposition ${\mathcal {G}}' = \bigcup \mathcal {S}'$ into coherent regimes which satisfy (3.22), (3.23), and (3.24).

We define ${\mathcal {G}}$ as

$$\begin{aligned}{\mathcal {G}}:= {\mathcal {G}}' \cap \{Q\in {\mathbb {D}}, \, \alpha _{\sigma }(Q) \le C^{-n}\epsilon _1^{n}\}\end{aligned}$$

where *C* is the constant in Lemma 3.13. Of course, it means that $\mathcal {B}:= \mathcal {B}' \cup ({\mathcal {G}}'{\setminus }{\mathcal {G}}).$ The coherent regimes $\mathcal {S}'$ may not be contained in ${\mathcal {G}},$ that is $\mathcal {S}' \cap {\mathcal {G}}$ may not be a coherent regime anymore. So we split further $\mathcal {S}' \cap {\mathcal {G}}$ into a disjoint union of (stopping time) coherent regimes $\{\mathcal {S}_{i}\}_{i\in I_{\mathcal {S}'}}$ that are maximal in the sense that the minimal cubes of $\mathcal {S}_{i}$ are those for which at least one children belongs to ${\mathbb {D}}_{\partial \Omega } {\setminus } (\mathcal {S}' \cap {\mathcal {G}}).$ The collection $\{\mathcal {S}\}_{\mathcal {S}\in {\mathfrak {S}}}$ is then the collection of all the $\mathcal {S}_{i}$ for $i\in I_{\mathcal {S}'}$ and $\mathcal {S}' \in {\mathfrak {S}}'.$

It remains to check that the ${\mathcal {G}},$ $\mathcal {B}$ and $\{\mathcal {S}\}_{\mathcal {S}\in {\mathfrak {S}}}$ that we just built satisfy (3.18) and (3.19). For the former, we use the fact that a regime $\mathcal {S}$ is necessarily included in a $\mathcal {S}',$ so for any $Q\in \mathcal {S},$ we have

3.26 $$\begin{aligned} {{\,\textrm{Angle}\,}}(P_Q,P_{Q(\mathcal {S})})\le & {} {{\,\textrm{Angle}\,}}(P_Q,P_{Q(\mathcal {S}')}) + {{\,\textrm{Angle}\,}}(P_{Q(\mathcal {S}')},P_{Q(\mathcal {S})}) \nonumber \\= & {} {{\,\textrm{Angle}\,}}(P'_Q,P'_{Q(\mathcal {S}')}) + {{\,\textrm{Angle}\,}}(P'_{Q(\mathcal {S})},P'_{Q(\mathcal {S}')}) \le 2 \epsilon _2 = \epsilon _0\nonumber \\ \end{aligned}$$

by (3.25), (3.23), and our choice of $\epsilon _2.$ The fact that $\mathcal {B}$ satisfies the Carleson packing condition

3.27 $$\begin{aligned} \sum _{{\begin{array}{c} Q\in \mathcal {B}\\ Q \subset Q_0 \end{array}}} \sigma (Q) \le C_{\epsilon _0,\epsilon _1} \sigma (Q_0) \quad \text {for all } Q_0\in {\mathbb {D}}_{\partial \Omega } \end{aligned}$$

is an immediate consequence of the definition of $\mathcal {B},$ (3.10), and (3.24). Finally, by the maximality of the coherent regimes $\mathcal {S},$ then either $Q(\mathcal {S})$ is the maximal cube of a coherent regime from the collection $\{\mathcal {S}'\}_{\mathcal {S}'\in {\mathfrak {S}}'},$ or (at least) the parent or one sibling of $Q(\mathcal {S}_i)$ belongs to $\mathcal {B}.$ Therefore, if $Q^*$ denotes the parent of a dyadic cube *Q*,  then for any $Q_0\in {\mathbb {D}}_{\partial \Omega },$

$$\begin{aligned} \sum _{{\begin{array}{c} \mathcal {S}\in {\mathfrak {S}} \\ Q(\mathcal {S}) \subset Q_0 \end{array}}} \sigma (Q(\mathcal {S})) \le \sum _{{\begin{array}{c} \mathcal {S}'\in {\mathfrak {S}}' \\ Q(\mathcal {S}') \subset Q_0 \end{array}}} \sigma (Q(\mathcal {S}')) + \sum _{{\begin{array}{c} Q\in \mathcal {B}\\ Q \subset Q_0 \end{array}}} \sigma (Q^*) \lesssim \sigma (Q_0) \end{aligned}$$

because of the Carleson packing conditions (3.24) and (3.27), and because $\sigma (Q^*) \approx \ell (Q)^{n-1} \approx \sigma (Q).$ The lemma follows. $\square$

### The approximating Lipschitz graph

In this subsection, we show that each coherent regime given by the corona decomposition is well approximated by a Lipschitz graph. We follow the outline of Section 8 in [18] except that we are a bit more careful about our construction in order to obtain Lemma 3.47 below. That is, instead of just wanting the Lipschitz graph $\Gamma _{\mathcal {S}}$ to be close to $\partial \Omega ,$ we aim to prove that the Lipschitz graph is an approximation of $\partial \Omega$ at least as good as the best plane.

Pick $0 < \epsilon _1 \ll \epsilon _0 \ll 1,$ and then construct the collection of coherent regimes ${\mathfrak {S}}$ given by Lemma 3.16. Take $\mathcal {S}$ to be either in ${\mathfrak {S}},$ or a coherent regime included in an element of ${\mathfrak {S}},$ and let it be fixed. Set $P := P_{Q(\mathcal {S})}$ and define $\Pi$ as the orthogonal projection on *P*. Similarly, we write $P^\bot$ for the line orthogonal to *P* and $\Pi ^\bot$ for the projection onto $P^\bot .$ We shall also need the function *d* on *P*: for $p\in P,$ define

3.28 $$\begin{aligned} d(p) := \inf _{Q\in \mathcal {S}} \{ {{\,\textrm{dist}\,}}(p,\Pi (2B_Q)) + \ell (Q)\}. \end{aligned}$$

We want to construct a Lipschitz function $b: \, P \mapsto P^\bot .$ First, we prove a small result. We claim that for $x,y\in \partial \Omega \cap 999B_{Q(\mathcal {S})},$ we have

3.29 $$\begin{aligned} |\Pi ^\bot (x) - \Pi ^\bot (y)| \le 2\epsilon _0 |\Pi (y) - \Pi (x)| \quad \text {whenever } |x-y| > 10^{-3} d(\Pi (x)). \nonumber \\ \end{aligned}$$

Indeed, with such choices of *x* and *y*,  we can find $Q\in \mathcal {S}$ such that

$$\begin{aligned} 0 < |x-y| \approx {{\,\textrm{dist}\,}}(\Pi (x),\Pi (Q)) + \ell (Q) \end{aligned}$$

and by taking an appropriate ancestor of *Q*,  we find $Q^*$ such that $|x-y| \approx \ell (Q^*).$ Since $x,y\in 999B_{Q(\mathcal {S})},$ we can always take $Q^* \subset Q(\mathcal {S})$—that is $Q^* \in \mathcal {S}$ thanks to the coherence of $\mathcal {S}$—and $x,y\in 999B_{Q^*}.$ Due to (3.17), we deduce that

$$\begin{aligned} {{\,\textrm{dist}\,}}(x,P_{Q^*}) + {{\,\textrm{dist}\,}}(y,P_{Q^*}) \le 2\epsilon _1 \ell (Q^*) \ll \epsilon _0 |x-y| \end{aligned}$$

if $\epsilon _1/\epsilon _0$ is sufficiently small. Since ${{\,\textrm{Angle}\,}}(P_{Q^*},P) \le \epsilon _0$ by (3.18), we conclude

$$\begin{aligned}{} & {} |\Pi ^\bot (x) - \Pi ^\bot (y)| \le {{\,\textrm{dist}\,}}(x,P_{Q^*}) + {{\,\textrm{dist}\,}}(y,P_{Q^*}) + \frac{1}{2} \epsilon _0 |x-y| \le \frac{3}{4} \epsilon _0 |x-y|\\{} & {} \quad \le \epsilon _0 |\Pi (x) - \Pi (y)| \end{aligned}$$

if $\epsilon _0$ is small enough. The claim (3.29) follows.

Define the closed set

3.30 $$\begin{aligned} Z = \{p\in P, \, d(p) = 0\}. \end{aligned}$$

The Lipschitz function *b* will be defined by two cases.

**Case** $d(p) = 0.$ That is, $p\in Z.$ In this case, since $\partial \Omega$ is closed, there necessarily exists $x\in \partial \Omega$ such that $\Pi (x) = p.$ Moreover, (3.29) shows that such *x* is unique, that is $\Pi$ is a one to one map on *Z*,  and we define

3.31 $$\begin{aligned} b(p) := \Pi ^\bot (\Pi ^{-1}(p)) \quad \text {for } p\in Z. \end{aligned}$$

**Case** $d(p)>0.$ We partition $P {\setminus } Z$ with a union of dyadic cubes, in the spirit of a Whitney decomposition, as follows. Construct the collection ${\mathcal {W}}_P$ as the subset of the dyadic cubes of *P* that are maximal for the property

3.32 $$\begin{aligned} 0 < 21 \ell (R) \le \inf _{q\in 3R} d(q). \end{aligned}$$

By construction, $d(p) \approx d(q)$ whenever $p,q\in 3R\in {\mathcal {W}}_P.$ Moreover, let us check that

3.33 $$\begin{aligned} \ell (R_1)/\ell (R_2) \in \{1/2,1,2\} \quad \text { if } R_1,R_2 \in {\mathcal {W}}_P \text { are s.t. } 3R_1 \cap 3R_2 \ne \emptyset . \end{aligned}$$

Indeed, if $R \in {\mathcal {W}}_P$ and *S* is such that $\ell (S) = \ell (R)$ and $3S \cap 3R \ne \emptyset ,$ then $3S \subset 9R$ and hence

$$\begin{aligned} 20\ell (S) = 20\ell (R) \le \inf _{p\in 3R} d(p) \le \inf _{p\in 9R} d(p) + 6\ell (R) \le \inf _{p\in 3S} d(p) + 6\ell (S). \end{aligned}$$

So every children of *S* has to satisfy (3.32), which proves (3.33).

By construction of ${\mathcal {W}}_P,$ for each $R\in {\mathcal {W}}_P,$ we can find $Q_R\in \mathcal {S}$ such that

3.34 $$\begin{aligned}{} & {} {{\,\textrm{dist}\,}}(R,\Pi (Q_R)) \le (2^6-2) \ell (R), \quad \ell (Q_R) \le 2^5 \ell (R),\nonumber \\{} & {} \quad \text { and either } Q_R =Q(\mathcal {S}) \text { or } \ell (Q_R) = 2^5\ell (R) \approx \inf _{q\in 2R} d(q) \approx \sup _{q\in 2R} d(q). \nonumber \\ \end{aligned}$$

We want to associate each *R* with an affine function $b_R: \, P \mapsto P^\bot$ such that the image of the function ${\mathfrak {b}}_R$ defined as ${\mathfrak {b}}_R(p) = (p,b_R(p))$ approximates $\partial \Omega$ well. First, we set

3.35 $$\begin{aligned} b_R \equiv 0 \quad \text {when } Q_R = Q({\mathcal {S}}). \end{aligned}$$

When $Q_R \ne Q({\mathcal {S}}),$ we take $b_R$ such that ${\mathfrak {b}}_R$ verifies

3.36 $$\begin{aligned}{} & {} \int _{999\Delta _{Q(\mathcal {S})}} |y - {\mathfrak {b}}_R(\Pi (y))| {\mathbb {1}}_{\Pi (y) \in 2R} \, d\sigma (y) \nonumber \\{} & {} \quad := \min _{a} \int _{999\Delta _{Q(\mathcal {S})}} |y - {\mathfrak {a}}_R(\Pi (y))| {\mathbb {1}}_{\Pi (y) \in 2R} \, d\sigma (y), \end{aligned}$$

where the minimum is taken over the affine functions $a:\, P \mapsto P^\bot$ and ${\mathfrak {a}}_R(p) := (p,a(p)).$ The uniqueness of the minimum is not guaranteed, but it does not matter for us. The existence is guaranteed, because $R\subset \Pi (3B_{Q_R}) \subset P \cap 999B_{Q_R}$ by (3.34), and hence (3.17) entails that the graph of the *a* that almost realize the infimum are very close to the plane $P_Q$ which makes a small angle with *P*. The same argument shows that

3.37 $$\begin{aligned} \sup _{y \in 999\Delta _{Q_R}} |y - {\mathfrak {b}}_R(\Pi (y))| + \sup _{p\in \Pi (999B_{Q_R})} {{\,\textrm{dist}\,}}({\mathfrak {b}}_R(p),\partial \Omega ) \le C \epsilon _1 \ell (Q_R).\nonumber \\ \end{aligned}$$

for a constant $C>0$ that depends only on *n* and

3.38 $$\begin{aligned} b_R~\text {is}~1.1\epsilon _0\text {-Lipschitz} \end{aligned}$$

if $0<\epsilon _1 \ll \epsilon _0 \ll 1.$ We associate to the collection ${\mathcal {W}}_P$ a partition of unity $\{\varphi _R\}_{R\in {\mathcal {W}}_P}$ such that $\varphi _R \in C^\infty _0(2R_i),$ $|\nabla \varphi _R| \lesssim \ell (R)^{-1},$ and $\sum _R \varphi _R \equiv 1$ on $P {\setminus }Z.$ We then define

3.39 $$\begin{aligned} b(p) := \sum _{R\in {\mathcal {W}}_P} \varphi _R(p) b_{R}(p) \quad \text {for } p\in P {\setminus }Z. \end{aligned}$$

Due to (3.33), the sum in (3.39) is finite and thus the quantity *b*(*p*) is actually well defined.

For $p\in P,$ we define ${\mathfrak {b}}(p) := (p,b(p))$ to be the graph of *b*.

#### Lemma 26

The function *b* defined by (3.31) and (3.39) is $2\epsilon _0$-Lipschitz and supported in $P\cap 4B_{Q(\mathcal {S})}.$

#### Proof

Recall that the property (3.34) implies that $2R \subset P \cap \Pi (3B_{Q_R})$ as long as $Q_R \ne Q(\mathcal {S}).$ So if $p\notin P\cap \Pi (3B_{Q(\mathcal {S})})$ and $R\in {\mathcal {W}}_P$ is such that $p\in 2R,$ we necessarily have $Q_R = Q(\mathcal {S})$ and then $b_R(p) = 0$ by (3.35). We conclude that $b(p) = 0$ and thus that *b* is supported in $P\cap \Pi (3B_{Q(\mathcal {S})}) \subset P \cap 4B_{Q(\mathcal {S})}.$

Now, we want to show that *b* is Lipschitz. The fact that *b* is Lipschitz on *Z* is an immediate consequence from the definition (3.31) and (3.29). Let us prove now that *b* is Lipschitz on the interior of $2R_0$ for every $R_0\in {\mathcal {W}}_P.$ Take $R_0 \in {\mathcal {W}}_P$ and $p\in 2R_0 {\setminus } \partial (2R_0).$ Then, since $\sum \nabla \varphi _R(p) = 0,$ we have

3.41 $$\begin{aligned} \begin{aligned} |\nabla b(p)|&= \left| \sum _{{\begin{array}{c} R\in {\mathcal {W}}_P \\ 2R \cap 2R_0 \ne \emptyset \end{array}}} \varphi _R(p) \nabla b_{Q_R}(p) + \sum _{{\begin{array}{c} R\in {\mathcal {W}}_P \\ 2R \cap 2R_0 \ne \emptyset \end{array}}} b_{Q_R}(p) \nabla \varphi _R(p) \right| \\&\le \sup _{{\begin{array}{c} R\in {\mathcal {W}}_P \\ 2R \cap 2R_0 \ne \emptyset \end{array}}} |\nabla b_{Q_R}(p)| + \sum _{{\begin{array}{c} R\in {\mathcal {W}}_P \\ 2R \cap 2R_0 \ne \emptyset \end{array}}} |\nabla \varphi _R(p)| |b_{Q_R}(p) - b_{Q_{R_0}}(p)| \\&\le 1.1\epsilon _0 + C \ell (R_0)^{-1} \sup _{{\begin{array}{c} R\in {\mathcal {W}}_P \\ 2R \cap 2R_0 \ne \emptyset \end{array}}} |b_{Q_R}(p) - b_{Q_{R_0}}(p)| \end{aligned}\nonumber \\ \end{aligned}$$

by (3.38) and (3.33). We can assume that $p\in 2R_0 \subset P\cap 4B_{Q(\mathcal {S})},$ because we have already shown that $b(p) = 0$ otherwise. So due to (3.34) and (3.33), both $Q_R$ and $Q_{R_0}$ are close to $2R_0,$ in the sense that

$$\begin{aligned} 3R_0 \subset P \cap 999\Pi (B_{Q_R}), \end{aligned}$$

so we can invoke (3.37) to say that ${{\,\textrm{dist}\,}}({\mathfrak {b}}_{Q_R}(p),P_{Q_{R_0}}) \lesssim \epsilon _1 \ell (Q_{R_0})$ and then

3.42 $$\begin{aligned} |b_{Q_R}(p) - b_{Q_{R_0}}(p)| \lesssim \epsilon _1 \ell (Q_{R_0}). \end{aligned}$$

So if $\epsilon _1 \ll \epsilon _0$ is small enough, (3.41) becomes $|\nabla b(p)| \le 2\epsilon _0.$

We proved that *b* is Lipschitz on *Z* and $P{\setminus }Z,$ so it remains to check that *b* is continuous at every point in $\partial Z.$ Take $z\in \partial Z$ and set $x:= {\mathfrak {b}}(z) \in \partial \Omega .$ Take also $p\in P{\setminus }Z$ such that $|p-z| \ll 1.$ Due to (3.37) and (3.42), we have the existence of $y\in \partial \Omega$ such that, for any $R\in {\mathcal {W}}_P$ satisfying $p\in 2R,$ we have

3.43 $$\begin{aligned} |y - {\mathfrak {b}}_{Q_R}(p)| \lesssim \epsilon _1 \ell (R) \lesssim \epsilon _1 d(p) \le \epsilon _1 |p-z| \end{aligned}$$

by (3.32) and the fact that $q\rightarrow d(q)$ is 1-Lipschitz. The latter bound shows in particular that

3.44 $$\begin{aligned} |y - {\mathfrak {b}}(p)| \le \epsilon _0 |p-z| \end{aligned}$$

if $\epsilon _1/\epsilon _0$ is small enough. The bound (3.44) also implies that $\Pi (x) \ne \Pi (y)$ and then $x \ne y,$ and so (3.29) entails that

3.45 $$\begin{aligned} |b(z) - \Pi ^\bot (y)| = |\Pi ^\bot (x) - \Pi ^\bot (y)| \le 2\epsilon _0|z - \Pi (y)|. \end{aligned}$$

The combination of (3.44) and (3.45) proves that the restriction of *b* to $P{\setminus }Z$ has the limit *b*(*z*) at the point $z\in \partial \Omega .$ Since it is true for all $z\in \partial Z,$ and since *b* is already continuous (even Lipschitz) on *Z* and $P{\setminus }Z,$ we conclude that *b* is continuous on *P*. The lemma follows. $\square$

We prove that the graph of *b* is well approximated by the same plane as the ones that approximate $\partial \Omega ,$ as shown below.

#### Lemma 27

For $Q\in \mathcal {S},$ we have

$$\begin{aligned} \sup _{p\in P \cap \Pi (2^8B_Q)} \Big [{{\,\textrm{dist}\,}}({\mathfrak {b}}(p),\partial \Omega ) + {{\,\textrm{dist}\,}}({\mathfrak {b}}(p), P_{Q}) \Big ] \lesssim \epsilon _1 \ell (Q). \end{aligned}$$

#### Proof

Take $p\in \Pi (2^8B_Q).$ If $p\in Z,$ then ${\mathfrak {b}}(p) \in \partial \Omega ,$ but since we also have (3.29), we deduce ${\mathfrak {b}}(p)\in 2^9\Delta _Q.$ The bound ${{\,\textrm{dist}\,}}({\mathfrak {b}}(p),P_Q) \le C\epsilon _1\ell (Q)$ is then just a consequence of (3.17).

Assume now that $p\in P{\setminus } Z.$ We have $d(p) \le 2^8\ell (Q)$ so any *R* that verifies $p\in 2R$ is such that $21\ell (R) \le d(p) \le 2^8\ell (Q)$ by (3.32), that implies $\ell (Q_R) \le 2^9\ell (Q)$ by (3.34). Since ${\mathfrak {b}}(p)$ is a weighted average of the ${\mathfrak {b}}_R(p),$ the estimate (3.37) on ${\mathfrak {b}}_R(p)$ gives that

$$\begin{aligned} {{\,\textrm{dist}\,}}({\mathfrak {b}}(p),\partial \Omega ) \lesssim \epsilon _1 \sup _{R: \, p\in 2R}\ell (Q_R) \lesssim \epsilon _1 \ell (Q). \end{aligned}$$

If $x\in \partial \Omega$ is such that $|{\mathfrak {b}}(p) - x| = {{\,\textrm{dist}\,}}({\mathfrak {b}}(p),\partial \Omega ),$ then we have again by (3.29) that $x\in 2^9\Delta _Q$ so (3.17) gives that ${{\,\textrm{dist}\,}}(x,P_Q) \le \epsilon _1\ell (Q).$ We conclude

$$\begin{aligned} {{\,\textrm{dist}\,}}({\mathfrak {b}}(p),P_Q) \le |{\mathfrak {b}}(p) - x| + {{\,\textrm{dist}\,}}(x,P_Q) \lesssim \epsilon _1\ell (Q) \end{aligned}$$

as desired. $$\square $$

We also need an $L^1$ version of the above lemma, and with a better control in terms of the $\alpha _{\sigma }(Q)$ (which is smaller than $\epsilon _1$ when $Q\in \mathcal {S})$.

#### Lemma 28

For $Q\in \mathcal {S},$ we have

$$\begin{aligned} \int _{P\cap \Pi (2B_{Q})} {{\,\textrm{dist}\,}}({\mathfrak {b}}(p), P_{Q}) \, dp \lesssim \ell (Q)^n \alpha _{\sigma }(Q). \end{aligned}$$

#### Proof

The plane *P* is the union of *Z* and $P{\setminus } Z := \bigcup _{R\in W_P} R,$ so

$$\begin{aligned}\begin{aligned} I&:= \int _{P\cap \Pi (2B_{Q})} {{\,\textrm{dist}\,}}({\mathfrak {b}}(p), P_{Q}) \, dp \\&= \int _{Z\cap \Pi (2B_{Q})} {{\,\textrm{dist}\,}}({\mathfrak {b}}(p), P_{Q}) \, dp + \int _{Z^c \cap \Pi (2B_{Q})}{{\,\textrm{dist}\,}}({\mathfrak {b}}(p), P_{Q}) \, dp := I_1 + I_2. \end{aligned} \end{aligned}$$

The term $I_1$ is easy, because ${\mathfrak {b}}(p) \in 4\Delta _Q \subset \partial \Omega$ by (3.29), and so we have

$$\begin{aligned} I_1 \lesssim \int _{4\Delta _Q} {{\,\textrm{dist}\,}}(y,P_Q) \, d\sigma (y) \end{aligned}$$

We apply (3.12) with the test function

$$\begin{aligned} f(y):= \min \{{{\,\textrm{dist}\,}}(y,\mathbb {R}^n {\setminus } 999B_{Q}), {{\,\textrm{dist}\,}}(y,P_{Q})\} \end{aligned}$$

which lies in $Lip_Q$ and takes the value 0 on $P_{Q}$ and ${{\,\textrm{dist}\,}}(y,P_{Q})$ on $4\Delta _{Q},$ and we conclude that

$$\begin{aligned} I_1 \lesssim \int f \, d\sigma = \left| \int f \, d\sigma - \int f \, d\mu _{Q}\right| \lesssim \ell (Q)^{n} \alpha _{\sigma }(Q) \end{aligned}$$

as desired.

We turn to the bound on $I_2.$ We know that ${{\,\textrm{Angle}\,}}(P_{Q},P) \le \epsilon _0$ so the plane $P_{Q}$ is the graph of an affine function $a_{Q}: \, P \mapsto P^\bot$ with small Lipschitz constant. Therefore, we have

$$\begin{aligned} I_2 \approx \int _{P\cap \Pi (2B_Q))} |b(p)- a_{Q}(p)| \, dp. \end{aligned}$$

Let ${\mathcal {W}}_P(Q)$ be the subfamily of ${\mathcal {W}}_P$ of elements *R* such that 2*R* that intersects $\Pi (2B_{Q}).$ The fact that $2R \cap \Pi (2B_{Q}) \ne \emptyset$ implies by (3.32) that $21 \ell (R) \le \ell (Q).$ Consequently, $\ell (R) \le 2^{-5} \ell (Q)$ because both $\ell (R)$ and $\ell (Q)$ are in the form $2^k,$ and then $2R \subset \Pi (3B_{Q}).$

Assume first that $Q \varsubsetneq Q(\mathcal {S}),$ and check that this condition implies that $\ell (Q_R) \le 2^5\ell (R) \le \ell (Q) < \ell (Q(\mathcal {S})),$ hence $Q_R \ne Q(\mathcal {S})$ for every $R\in {\mathcal {W}}_P(Q).$ So we have

$$\begin{aligned} I_2= & {} \int _{Z^c \cap \Pi (2B_{Q})} \left| \sum _{R\in {\mathcal {W}}_P(Q)} \varphi _R(p) (b_{R}(p) - a_{Q}(p))\right| \, dp \\\le & {} \sum _{R\in {\mathcal {W}}_P(Q)} \int _{2R} |b_{R}(p) - a_{Q}(p)| \, dp. \end{aligned}$$

We want to estimate $\int _{2R} |b_{R}(p) - a_{Q}(p)| \, dp,$ but now both $b_R$ and $a_{Q}$ are affine, so knowing $|b_{R}(p) - a_{Q}(p)|$ for *n* different points $p\in 2R$ that are far from each other is enough. By (3.17), we know that $\Pi (\partial \Omega ) \cap 2R$ contains many points all over 2*R*,  and by using those points to estimate the distance between $b_R$ and $a_{Q},$ we deduce that

$$\begin{aligned} \int _{2R} |b_{R}(p) - a_{Q}(p)| \, dp\lesssim & {} \int _{999\Delta _{Q(\mathcal {S})}} |b_R(\Pi (y)) - a_{Q}(\Pi (y)) | {\mathbb {1}}_{\Pi (y) \in 2R} \, d\sigma (y) \\\le & {} \int _{999\Delta _{Q(\mathcal {S})}} |\Pi ^{\bot }(y) - b_R(\Pi (y))| {\mathbb {1}}_{\Pi (y) \in 2R} \, d\sigma (y) \\{} & {} + \int _{999\Delta _{Q(\mathcal {S})}} |\Pi ^\bot (y) - a_{Q}(\Pi (y)) | {\mathbb {1}}_{\Pi (y) \in 2R} \, d\sigma (y) \\\lesssim & {} \int _{999\Delta _{Q(\mathcal {S})}} |\Pi ^\bot (y) - a_{Q}(\Pi (y)) | {\mathbb {1}}_{\Pi (y) \in 2R} \, d\sigma (y) \end{aligned}$$

by (3.36), because $Q_R \ne Q(\mathcal {S}).$ Since the 2*R* are finitely overlapping, see (3.33), the bound on $I_2$ becomes

3.48 $$\begin{aligned} I_2 \lesssim \int _{4\Delta _{Q}} |\Pi ^\bot (y) - a_{Q}(\Pi (y)) | \, d\sigma (y) \lesssim \int _{4\Delta _{Q}} {{\,\textrm{dist}\,}}(y,P_{Q}) \, d\sigma (y). \end{aligned}$$

We had the same bound on $I_1,$ and with the same strategy, we can conclude that

$$\begin{aligned} I_2 \lesssim \int f \, d\sigma = \left| \int f \, d\sigma - \int f \, d\mu _{Q}\right| \lesssim \ell (Q)^{n} \alpha _{\sigma }(Q) \end{aligned}$$

as desired.

If $Q = Q(\mathcal {S}),$ the same computations apply. It is possible to have some *R* in ${\mathcal {W}}_P(Q)$ for which $Q_R = Q(\mathcal {S})$ and thus $b_R \equiv 0,$ but at the same time, we now have $a_Q \equiv 0,$ so those *R* verify $b_R - a_Q \equiv 0$ and do no have any contribution in the above bounds on $I_2.$ Therefore, we also conclude that

$$\begin{aligned} I_2 \lesssim \ell (Q(\mathcal {S}))^{n} \alpha _{\sigma }(Q(\mathcal {S})) = \ell (Q)^{n} \alpha _{\sigma }(Q). \end{aligned}$$

The lemma follows. $\square$

## Whitney regions for coherent regimes

We associate the dyadic cubes of $\partial \Omega$ to Whitney regions in $\Omega$ and therefore associate the coherent family of dyadic cubes obtained in the corona decomposition to a subset of $\Omega .$ The idea is similar to the construction found in [36], but we need different properties than those in [36], so we rewrite the construction.

This section will prove the following extension of Lemma 3.1.

### Lemma 29

Let $$\partial \Omega $$ be a uniformly rectifiable set. We keep the notation from Lemma 3.1, and we further have the existence of $$K^{**}>0$$ and a collection $$\{\Psi _{\mathcal {S}}\}_{\mathcal {S}\in {\mathfrak {S}}}$$ of functions such that

- $\Psi _{\mathcal {S}}$ are cut-off functions, that is $0 \le \Psi _{\mathcal {S}}\le 1,$ and $|\nabla \Psi _{\mathcal {S}}| \le 2\delta ^{-1}.$
- For any $\mathcal {S}\in {\mathfrak {S}},$ if $X \in {{\,\textrm{supp}\,}}(1-\Psi _{\mathcal {S}}),$ then there exists $Q\in \mathcal {S}$ such that
  $$\begin{aligned} \ell (Q)/2 < \delta (X) = {{\,\textrm{dist}\,}}(X,Q) \le \ell (Q). \end{aligned}$$
- If $X \in {{\,\textrm{supp}\,}}\Psi _{\mathcal {S}},$ then there exists $Q\in {\mathcal {S}}$ such that
  $$\begin{aligned} \ell (Q)/2^6 < \delta (X) = {{\,\textrm{dist}\,}}(X,2^6\Delta _Q) \le 2^6 \ell (Q). \end{aligned}$$
- For any $\mathcal {S}\in {\mathfrak {S}}$ and any $X\in {{\,\textrm{supp}\,}}\Psi _{\mathcal {S}},$ we have
  4.2 $$\begin{aligned} (1-2\epsilon _0) |X - {\mathfrak {b}}(\Pi (X))| \le \delta (X) \le (1+2\epsilon _0) |X - {\mathfrak {b}}(\Pi (X))| \end{aligned}$$
  and,  if $\Gamma _{\mathcal {S}}$ is the graph of $\mathcal {S},$
  4.3 $$\begin{aligned} (1-2\epsilon _0) {{\,\textrm{dist}\,}}(X,\Gamma _{\mathcal {S}}) \le \delta (X) \le (1+3\epsilon _0) {{\,\textrm{dist}\,}}(X,\Gamma _{\mathcal {S}}). \end{aligned}$$
- There exists a collection of dyadic cubes $\{Q_i\}_{i\in I_{\mathcal {S}}}$ in ${\mathbb {D}}_{\partial \Omega }$ such that $\{2Q_i\}_{i\in I_{\mathcal {S}}}$ has an overlap of at most 2,  and
  $$\begin{aligned} \Omega \cap \, {{\,\textrm{supp}\,}}\Psi _{\mathcal {S}}(1-\Psi _{\mathcal {S}}) \subset \bigcup _{i\in I_{\mathcal {S}}} \Bigg \{\frac{\ell (Q_i)}{K^{**}} < \delta (X) = {{\,\textrm{dist}\,}}(X,K\Delta _{Q_i}) \le K^{**}\ell (Q_i)\Bigg \}. \end{aligned}$$
  In particular,  $|\delta \nabla \Psi _{\mathcal {S}}| \in CM_{\Omega }(C)$ with a constant $C>0$ that depends only on *n*.

### Whitney decomposition

We divide $\Omega$ into Whitney regions. Usually, one constructs them with dyadic cubes of $\mathbb {R}^n,$ but we prefer to construct them directly. We recall that $\delta (X):= {{\,\textrm{dist}\,}}(X,\partial \Omega ),$ and for $Q\in {\mathbb {D}}_{\partial \Omega },$ we define

4.4 $$\begin{aligned} W_{\Omega }(Q) := \{X\in \Omega : \, \exists \, x\in Q \text { such that } \ell (Q)/2 < \delta (X) = |X-x| \le \ell (Q)\}. \nonumber \\ \end{aligned}$$

It is easy to see that the sets $\{W_{\Omega }(Q)\}_{Q\in {\mathbb {D}}_{\partial \Omega }}$ covers $\Omega .$ The sets $W_{\Omega }(Q)$ are not necessarily disjoint, but we do not care, we are perfectly happy if $\{W_{\Omega }(Q)\}_{Q\in {\mathbb {D}}_{\partial \Omega }}$ is finitely overlapping, and we choose $W_{\Omega }(Q)$ small only because it will make our estimates easier. The sets $W_{\Omega }(Q)$ can be disconnected and have a bad boundary, but that is not an issue, since—contrary to [36]—we won’t try to prove that the $W_{\Omega }(Q)$ are Chord-Arc Domains.

We also need fattened versions of $$W_{\Omega }(Q),$$ that we call $$W_{\Omega }^*(Q)$$ and $$W_{\Omega }^{**}(Q),$$ which are defined as

4.5 $$\begin{aligned} W_{\Omega }^*(Q) := \{X\in \Omega : \, \exists \, x\in 2^6\Delta _Q \text { s.t.} 2^{-6}\ell (Q) < \delta (X) = |X-x| \le 2^6\ell (Q)\} \nonumber \\ \end{aligned}$$

and

4.6 $$\begin{aligned} W_{\Omega }^{**}(Q) := \{X\in \Omega : \, \exists \, x\in K^{**}\Delta _Q \text { s.t. } \frac{\ell (Q)}{K^{**}} < \delta (X) = |X-x| \le K^{**}\ell (Q)\}.\nonumber \\ \end{aligned}$$

The exact value of the constant $$K^{**}$$ does not matter. In Lemma 4.28, we will choose it large enough to fit our purpose. The first properties of $$W_{\Omega }(Q)$$ and $$W_{\Omega }^*(Q)$$ are the ones that we expect and are easy to prove. We have

4.7 $$\begin{aligned} \Omega= & {} \bigcup _{Q\in {\mathbb {D}}_{\partial \Omega }} W_{\Omega }(Q), \end{aligned}$$

4.8 $$\begin{aligned} {{\,\textrm{diam}\,}}(W^{*}_{\Omega }(Q))\le & {} 2^7 \ell (Q), \end{aligned}$$

and

4.9 $$\begin{aligned} W^{*}_{\Omega }(Q) \subset 2^8 B_Q. \end{aligned}$$

We want $W_{\Omega }(Q)$ and $W_{\Omega }^*(Q)$ to be so that we can squeeze a cut-off function between the two sets, which is possible because

4.10 $$\begin{aligned} {{\,\textrm{dist}\,}}(W_{\Omega }(Q), \mathbb {R}^n {\setminus }W^*_{\Omega }(Q)) \ge \frac{1}{4} \ell (Q). \end{aligned}$$

Indeed, if $X\in W_{\Omega }(Q)$ and $|X-Y| \le \ell (Q)/4,$ then $\ell (Q)/4 \le {{\,\textrm{dist}\,}}(Y,\partial \Omega ) \le 5\ell (Q)/4$ and for any $y \in \partial \Omega$ such that $|Y-y| = \delta (Y),$ we have

$$\begin{aligned} |y-x| \le |y-Y| + |Y-X| + |X-x| \le \frac{5}{4}\ell (Q) + \frac{1}{4} \ell (Q) + \ell (Q) \le 3\ell (Q), \end{aligned}$$

so in particular, $y\in 2^5Q,$ and thus $Y\in W^*_{\Omega }(Q).$ The claim (4.10) follows.

### Coherent regions associated to coherent regimes

As before, we pick $0 < \epsilon _1 \ll \epsilon _0 \ll 1,$ and then construct the collection of coherent regimes ${\mathfrak {S}}$ given by Lemma 3.16. Let then $\mathcal {S}$ be either in ${\mathfrak {S}},$ or a coherent regime included in an element of ${\mathfrak {S}}.$ For such $\mathcal {S},$ we define the regions

4.11 $$\begin{aligned} W_{\Omega }(\mathcal {S}) := \bigcup _{Q\in \mathcal {S}} W_{\Omega }(Q) \quad \text { and } \quad W_{\Omega }^*(\mathcal {S}) := \bigcup _{Q\in \mathcal {S}} W^*_{\Omega }(Q). \end{aligned}$$

Associated to the coherent regime $\mathcal {S},$ we have affine planes *P* and $P^\bot ,$ the projections $\Pi$ and $\Pi ^\bot ,$ a Lipschitz function $b: \, P \rightarrow P^\bot ,$ and ${\mathfrak {b}}(p)=(p,b(p))$ as in Subsection 3.4. We also have the “distance function” *d*(*p*) defined in (3.28). We now define the Lipschitz graph

4.12 $$\begin{aligned} \Gamma _{\mathcal {S}} := \{{\mathfrak {b}}(p), \, p\in P\} \subset \mathbb {R}^n. \end{aligned}$$

#### Lemma 30

If $X\in W^*_{\Omega }(\mathcal {S})$ and $x\in \partial \Omega$ is such that $|X-x| = \delta (X),$ then

4.14 $$\begin{aligned}{} & {} (1-2\epsilon _0) \delta (X) \le |X - {\mathfrak {b}}(\Pi (X))| \le (1+2\epsilon _0) \delta (X), \end{aligned}$$

4.15 $$\begin{aligned}{} & {} (1-2\epsilon _0) {{\,\textrm{dist}\,}}(X,\Gamma _{\mathcal {S}}) \le \delta (X) \le (1+3\epsilon _0) {{\,\textrm{dist}\,}}(X,\Gamma _{\mathcal {S}}), \end{aligned}$$

and

4.16 $$\begin{aligned} |{\mathfrak {b}}(\Pi (X)) - x | \le 2\epsilon _0 \delta (X). \end{aligned}$$

#### Proof

Since $X\in W^*_{\Omega }(\mathcal {S}),$ there exists $Q\in \mathcal {S}$ such that $X\in W^*_{\Omega }(Q).$ Such *Q* verifies $x\in 2^6\Delta _Q$ and

$$\begin{aligned} 2^{-6} |X- x| \le \ell (Q) \le 2^6 |X-x|, \end{aligned}$$

so $X \in 2^7 B_Q$ and $\Pi (X) \in \Pi (2^7 B_Q).$ Lemma 3.46 and (3.17) entail that

$$\begin{aligned} {{\,\textrm{dist}\,}}(x,P_Q) + {{\,\textrm{dist}\,}}({\mathfrak {b}}(\Pi (X)),P_Q) \le C \epsilon _1 \ell (Q) \le \frac{1}{8} \epsilon _0 |X-x| \end{aligned}$$

if $\epsilon _1/\epsilon _0$ is small enough. Because the plane $P_Q$ makes a small angle with *P*,  we deduce that

4.17 $$\begin{aligned} |b(\Pi (X)) - \Pi ^\bot (x)| \le \frac{1}{4} \epsilon _0 |X-x| \end{aligned}$$

if $\epsilon _0$ is small enough. Define $\Pi _Q$ and $\Pi ^\bot _Q$ as the projection onto $P_Q$ and $P_Q^\bot .$ We have $|\Pi _Q(x) - x| \lesssim \epsilon _1 |X-x|$ thanks to (3.17). In addition, the projection $\Pi _Q(X)$ lies in $P_Q \cap 2^8 B_Q,$ so using (3.17) again gives the existence of $y\in \partial \Omega$ such that $|\Pi _Q(X) - y| \le \epsilon _1\ell (Q) \lesssim \epsilon _1 |X-x|.$ By definition of *x*,  the point *y* has to be further away from *X* than *x* so

$$\begin{aligned} \begin{aligned} |X-x|&\le |X-y| \\&\le |X - \Pi _Q(X) - x + \Pi _Q(x)| + |\Pi _Q(x) - x| + |\Pi _Q(X) - y| \\&\le |\Pi ^\bot _Q(X)- \Pi ^\bot _Q(x)| + C\epsilon _1|X-x|. \end{aligned} \end{aligned}$$

So one has $|\Pi ^\bot _Q(X)-\Pi ^\bot _Q(x)| \ge (1-C\epsilon _1) |X-x|$ and hence we have the bound $|\Pi _Q(X)- \Pi _Q(x)| \le C\sqrt{\epsilon _1}|X-x|.$ Since $P_Q$ makes an angle at most $\epsilon _0$ with *P*,  we conclude that

4.18 $$\begin{aligned} |\Pi (X) - \Pi (x)| \le \frac{3}{2}\epsilon _0|X-x| \end{aligned}$$

if $\epsilon _0$ and $\epsilon _1/\epsilon _0$ are small enough. The two bounds (4.17) and (4.18) easily prove (4.16), and also prove (4.14) by writing

$$\begin{aligned}{} & {} \Big | |X - {\mathfrak {b}}(\Pi (X))| - |X-x| \Big | \\{} & {} \quad \le \Big | |\Pi ^\bot (X) - b(\Pi (X))| - |\Pi ^\bot (X)- \Pi ^\bot (x)| \Big | + |\Pi (X) - \Pi (x)| \\{} & {} \quad \le |\Pi ^\bot (x) - b(\Pi (X))| + |\Pi (X) - \Pi (x)| \le 2\epsilon _0|X-x|. \end{aligned}$$

The bounds (4.15) is just a consequence of (4.14) and the fact that $\Gamma _{\mathcal {S}}$ is the graph of *b* which is a $2\epsilon _0$-Lipschitz function with $\epsilon _0 \ll 1.$ The lemma follows. $\square$

Let $\psi \in C^\infty _0(\mathbb {R})$ be such that $0 \le \psi \le 1,$ $\psi \equiv 1$ on [0, 1],  $\psi \equiv 0$ on $[2,\infty )$ and $|\nabla \psi | \le 2.$ We set

4.19 $$\begin{aligned} \Psi _{\mathcal {S}}(X) = {\mathbb {1}}_{\Omega }(X) \psi \Bigg (\frac{d(\Pi (X))}{3|X - {\mathfrak {b}}(\Pi (X))|}\Bigg ) \psi \Bigg (\frac{|X - {\mathfrak {b}}(\Pi (X))|}{2\ell (Q(\mathcal {S}))}\Bigg ). \end{aligned}$$

We want to prove the points (b), (c), and (d) of Lemma 4.1, that is

#### Lemma 31

The function $\Psi _{\mathcal {S}}$ is constant equal to 1 on $W_{\Omega }(\mathcal {S})$ and $\Omega \cap {{\,\textrm{supp}\,}}\Psi _{\mathcal {S}} \subset W^*_{\Omega }(\mathcal {S}).$ Consequently,  for any $X\in {{\,\textrm{supp}\,}}\Psi _{\mathcal {S}} ,$ we have (4.2) and (4.3) by Lemma 4.13.

#### Remark 4.21

We know from its definition that $\Psi _{\mathcal {S}} \equiv 0$ on $\mathbb {R}^n {\setminus } \Omega ,$ but the support of $\Psi _{\mathcal {S}}$ can reach the boundary $\partial \Omega .$ So if $\Omega \cap {{\,\textrm{supp}\,}}\Psi _{\mathcal {S}} \subset W_{\Omega }^*(\mathcal {S}),$ then we actually have

$$\begin{aligned} {{\,\textrm{supp}\,}}\Psi _{\mathcal {S}} \subset W_{\Omega }^*(\mathcal {S}) \cup \Big ( \partial \Omega \cap \overline{W_{\Omega }^*(\mathcal {S})} \Big ). \end{aligned}$$

#### Proof

Take $Q\in \mathcal {S}$ and $X\in W_{\Omega }(Q),$ and pick $x\in Q$ such that $|X-x| = \delta (X).$ We want to show that $\Psi _{\mathcal {S}} (X) = 1,$ i.e. that

4.22 $$\begin{aligned} d(\Pi (X)) \le 3|X - {\mathfrak {b}}(\Pi (X))| \end{aligned}$$

and

4.23 $$\begin{aligned} |X - {\mathfrak {b}}(\Pi (X))| \le 2\ell (Q(\mathcal {S})). \end{aligned}$$

For (4.23), it suffices to notice that $|X - {\mathfrak {b}}(\Pi (X))| \le 2 |X-x| \le 2\ell (Q) \le 2\ell (Q(\mathcal {S}))$ by (4.14) and by the definition of *x* and *Q*. As for (4.22), observe that $\left| X-x\right| \le 2\epsilon _0\delta (X)+\left| X-{\mathfrak {b}}(\Pi (X))\right|$ by the triangle inequality and (4.16), and thus

$$\begin{aligned} d(\Pi (X)) \le \ell (Q) \le 2|X-x| \le 3 |X - {\mathfrak {b}}(\Pi (X))| \end{aligned}$$

by (4.14).

It remains to verify that ${{\,\textrm{supp}\,}}\Psi _{\mathcal {S}}$ is supported in $W^*_{\Omega }(\mathcal {S}),$ because (4.2) and (4.3) are then just (4.14) and (4.15). So we pick $X\in {{\,\textrm{supp}\,}}\Psi _{\mathcal {S}}$ which means in particular that

4.24 $$\begin{aligned} d(\Pi (X)) \le 6|X - {\mathfrak {b}}(\Pi (X))| \end{aligned}$$

and

4.25 $$\begin{aligned} |X - {\mathfrak {b}}(\Pi (X))| \le 4\ell (Q(\mathcal {S})), \end{aligned}$$

and we want to show that $X\in W^*_{\Omega }(\mathcal {S}).$ By the definition of $d(\Pi (X)),$ there exists $Q \in \mathcal {S}$ such that

$$\begin{aligned} {{\,\textrm{dist}\,}}(\Pi (X),\Pi (2B_Q)) + \ell (Q) = d(\Pi (X)) \le 6 |X - {\mathfrak {b}}(\Pi (X))| \le 24 \ell (Q(\mathcal {S})) \end{aligned}$$

by (4.24) and (4.25). Since $\mathcal {S}$ is coherent, by taking a suitable ancestor of *Q*,  we can find $Q_X \in \mathcal {S}$ such that

4.26 $$\begin{aligned} \frac{1}{4} |X - {\mathfrak {b}}(\Pi (X))| \le \ell (Q_X) \le 6 |X - {\mathfrak {b}}(\Pi (X))| \end{aligned}$$

and

4.27 $$\begin{aligned} \Pi (X) \in 26 \Pi (B_{Q_X}). \end{aligned}$$

We want to prove that $X\in W_{\Omega }^*(Q_X).$ The combination of (4.27), Lemma 3.46, and (3.29) forces ${\mathfrak {b}}(\Pi (X)) \in 27B_{Q(\mathcal {S})}$ when $\epsilon _0$ is small, and hence $X \in 31B_{Q_X}$ by (4.26). Let $x\in \partial \Omega$ such that $|X-x| = \delta (X).$ Since $X\in 31B_{Q_X},$ we have $x\in 2^6\Delta _{Q_X},$ and of course $|X-x| \le 2^6\ell (Q_{X}).$ So it remains to verify if $|X-x| \ge 2^{-6} \ell (Q_X).$ In one hand, thanks to (3.17), we know that *x* lies close to $P_Q,$ in the sense that ${{\,\textrm{dist}\,}}(x,P_{Q_X}) \le \epsilon _1 \ell (Q_X).$ In the other hand, if $P_{Q_X}$ is the graph of the function $a_{Q_X}: \, P \mapsto P^\bot ,$ we have

$$\begin{aligned} \begin{aligned} {{\,\textrm{dist}\,}}(X,P_{Q_X})&\ge (1-\epsilon _0) |\Pi ^\bot (X) - a_{Q_X}(\Pi (X))| \\&\ge (1-\epsilon _0) \Big [ |X-{\mathfrak {b}}(\Pi (X))| - {{\,\textrm{dist}\,}}({\mathfrak {b}}(\Pi (X)),P_{Q_X}) \Big ] \\&\ge (1-\epsilon _0-C\epsilon _1) |X-{\mathfrak {b}}(\Pi (X))| \\&\ge \frac{1}{6}(1-\epsilon _0-C\epsilon _1) \ell (Q_X) \end{aligned} \end{aligned}$$

by Lemma 3.46 and (4.26); that is *X* is far from $P_{Q_X}.$ Altogether, we deduce that

$$\begin{aligned} |X-x| \ge (1-C\epsilon _1) {{\,\textrm{dist}\,}}(X,P_{Q_X}) \ge \frac{1}{8} \ell (Q_X). \end{aligned}$$

if $\epsilon _0$ and $\epsilon _1$ are small. The lemma follows. $\square$

We are left with the proof of point (e) in Lemma 4.1, which is:

#### Lemma 33

There exists a collection of dyadic cubes $\{Q_i\}_{i\in I_{\mathcal {S}}}$ in ${\mathbb {D}}_{\partial \Omega }$ such that $\{2Q_i\}_{i\in I_{\mathcal {S}}}$ has an overlap of at most 2,  and

$$\begin{aligned} \Omega \cap ({{\,\textrm{supp}\,}}\Psi _{\mathcal {S}}) \cap {{\,\textrm{supp}\,}}(1-\Psi _{\mathcal {S}}) \subset \bigcup _i W^{**}_{\Omega }(Q_i). \end{aligned}$$

#### Proof

Observe that $({{\,\textrm{supp}\,}}\Psi _{\mathcal {S}}) \cap {{\,\textrm{supp}\,}}(1-\Psi _{\mathcal {S}}) \subset E_1 \cup E_2$ where

$$\begin{aligned} E_1 := \{X\in W_{\Omega }^*(\mathcal {S}), \, 2\ell (Q(\mathcal {S})) \le |X- {\mathfrak {b}}(\Pi (X))| \le 4\ell (Q(\mathcal {S}))\} \end{aligned}$$

and

$$\begin{aligned} E_2 := \{X\in W_{\Omega }^*(\mathcal {S}), \, d(\Pi (X))/6 \le |X- {\mathfrak {b}}(\Pi (X))| \le d(\Pi (X))/3\}. \end{aligned}$$

Thanks to (4.2), the set $E_1$ is included in $W^{*}_{\Omega }(Q(\mathcal {S})).$

For each $X\in E_2,$ we construct the ball $B_X:= B({\mathfrak {b}}(\Pi (X)),d(\Pi (X))/100)$ in $\mathbb {R}^n.$ The radius of $B_X$ is bounded uniformly by $\ell (Q(\mathcal {S}))/4.$ So by the Vitali lemma, we can find a non overlapping subfamily $\{B_{X_i}\}_{i\in I_2}$ such that $E_2 \subset \bigcup _{i\in I_2} 5B_{X_i}.$ We use (4.16) and (4.14) to find a point $x_i \in \frac{1}{2}B_{X_i} \cap \partial \Omega .$ We take $Q_i \in {\mathbb {D}}_{\partial \Omega }$ to be the unique dyadic cube such that $x_i \in Q_i$ and $\ell (Q_i) < d(\Pi (X_i))/400 \le 2\ell (Q_i).$ By construction, we have $2Q_i \subset B_{X_i},$ so the $\{2Q_i\}_{i\in I_2}$ are non-overlapping, and $5B_{X_i} \subset 100B_{Q_i}.$

It remains to check that $$E_2 \subset \bigcup _i W^{**}_{\Omega }(Q_i).$$ Take $$X\in E_2.$$ From what we proved, there exists an $$i\in I_2$$ such that

4.29 $$\begin{aligned} |\Pi (X) - \Pi (X_i)| \le |{\mathfrak {b}}(\Pi (X))- {\mathfrak {b}}(\Pi (X_i))| \le d(\Pi (X_i))/20. \end{aligned}$$

Observe from the definition that *d* is 1-Lipschitz. Therefore,

$$\begin{aligned} |d(\Pi (X)) - d(\Pi (X_i))| \le |\Pi (X) - \Pi (X_i)| \le d(\Pi (X_i))/20 \end{aligned}$$

and

4.30 $$\begin{aligned} \frac{19}{20} d(\Pi (X_i)) \le d(\Pi (X)) \le \frac{21}{20} d(\Pi (X_i)). \end{aligned}$$

From (4.29) and (4.30), we obtain

$$\begin{aligned} |X-x_i|\le & {} |X-{\mathfrak {b}}(\Pi (X))| + |{\mathfrak {b}}(\Pi (X)) - {\mathfrak {b}}(\Pi (X_i))| + |{\mathfrak {b}}(\Pi (X_i)) - x_i|\\\le & {} d(\Pi (X)) \le 800\ell (Q_i) \end{aligned}$$

and, from (4.2) and (4.30), we get

$$\begin{aligned} \delta (X) \ge (1-2\epsilon _0)|X-{\mathfrak {b}}(\Pi (X))| \ge \frac{1}{7} d(\Pi (X)) \ge \frac{1}{8} d(\Pi (X_i)) \ge 50\ell (Q_i). \end{aligned}$$

The last two computations show that $$X\in W^{**}_{\Omega }(Q_i)$$ if $$K^{**} \ge 1601.$$ The lemma follows. $$\square $$

## Replacement lemma and application to the smooth distance *D*

As usual, let $0 < \epsilon _1 \ll \epsilon _0 \ll 1,$ and then construct the collection of coherent regimes ${\mathfrak {S}}$ given by Lemma 3.16. We take then $\mathcal {S}$ to be either in ${\mathfrak {S}},$ or a coherent regime included in an element of ${\mathfrak {S}}.$

In Lemma 3.47, we started to show that the graph of *b* behaves well with respect to the approximating planes $P_Q,$ and we want to use the graph of *b* as a substitute for $\partial \Omega .$ Roughly speaking, the graph of the Lipschitz function *b* is “a good approximation of $\partial \Omega$ for the regime $\mathcal {S}$”. Let us explain what we mean by this. The Lipschitz graph $\Gamma _{\mathcal {S}}$ defined in (4.12) is uniformly rectifiable, that is, $\Gamma _{\mathcal {S}}$ is well approximated by planes. And even better, we can easily construct explicit planes that approximate $\Gamma _{\mathcal {S}}.$

First, we equip *P* with an Euclidean structure, which means that *P* can be identified to $\mathbb {R}^{n-1}.$ Similarly, we identify $P^\bot$ to $\mathbb {R},$ and of course, we choose *P* and $P^\bot$ such that $\Pi ^{\bot }(P) = \{0\}$ and $\Pi (P^{\bot }) = \{0\},$ and so $\mathbb {R}^n$ can be identified to $P \times P^\bot .$

We take a non-negative radial smooth function $\eta \in C^\infty _0(P,\mathbb {R}_+)$ which is supported in the unit ball and that satisfies $\int _{P} \eta dx = 1.$ Even if *P* depends on the regime $\mathcal {S},$ *P* is identified to $\mathbb {R}^{n-1},$ so morally the smooth function $\eta$ is defined on $\mathbb {R}^{n-1}$ and does not depend on anything but the dimension *n*. For $t\ne 0,$ we construct the approximation of identity by

5.1 $$\begin{aligned} \eta _t(p) := \left| t\right| ^{1-n} \eta \Big (\frac{p}{\left| t\right| }\Big ), \end{aligned}$$

then the functions

5.2 $$\begin{aligned} b^t := \eta _t * b, \quad {\mathfrak {b}}^t := \eta _t * {\mathfrak {b}}, \end{aligned}$$

and the planes

5.3 $$\begin{aligned} \Lambda (p,t):= \{(q,(q-p) \nabla b^t(p) + b^t(p)), \, q\in P\}. \end{aligned}$$

Notice that the plane $\Lambda (p,t)$ is tangent to the graph $\left\{ {\mathfrak {b}}^t(p), \, p\in P\right\}$ at ${\mathfrak {b}}^t(p).$ What we actually want is flat measures, so we fix a radial function $\theta \in C^\infty (\mathbb {R}^n)$ such that $0\le \theta \le 1,$ ${{\,\textrm{supp}\,}}\theta \subset B(0,1),$ and $\theta \equiv 1$ on $B(0,\frac{1}{2}).$ We set then

5.4 $$\begin{aligned} \theta _{p,t}(y) := \theta \left( \frac{{\mathfrak {b}}^t(p) - y}{t} \right) \end{aligned}$$

and

5.5 $$\begin{aligned} \lambda (p,t) := \dfrac{\displaystyle \int _{\partial \Omega } \theta _{p,t} \, d\sigma }{\displaystyle \int _{\Lambda (p,t)} \theta _{p,t} \, d\mu _{\Lambda (p,t)}} = c_\theta ^{-1} \left| t\right| ^{1-n} \int _{\partial \Omega } \theta _{p,t} \, d\sigma \end{aligned}$$

where the second equality uses the fact that we centered $\theta _{p,t}$ at ${\mathfrak {b}}^t(p) \in \Lambda (p,t),$ and $c_\theta := \int _{\mathbb {R}^{n-1}} \theta (y) dy.$ Note that the Ahlfors regularity of $\sigma$ implies that

5.6 $$\begin{aligned} \lambda (p,t) \approx 1, \end{aligned}$$

whenever ${\mathfrak {b}}^t(p)$ is close to $\partial \Omega$—which is the case when $d(p) \lesssim \left| t\right| \lesssim \ell (Q(\mathcal {S}))$—and with constants that depend only on $C_{\sigma }$ and *n*. Finally, we introduce the flat measures

5.7 $$\begin{aligned} \mu _{p,t} := \lambda (p,t) \mu _{\Lambda (p,t)}. \end{aligned}$$

The flat measures $\mu _{p,t}$ are approximations of the Hausdorff measure on $\Gamma _{\mathcal {S}},$ and we shall show that the same explicit measures almost minimize the distance from $\sigma$ to flat measures, for the local Wasserstein distances ${{\,\textrm{dist}\,}}_Q$ with $Q\in \mathcal {S}.$

### Lemma 34

For $Q\in \mathcal {S},$ $p\in \Pi (\frac{3}{2}B_Q),$ and $\ell (Q)/4 \le \left| t\right| \le \ell (Q)/2,$ we have

5.9 $$\begin{aligned} {{\,\textrm{dist}\,}}_Q(\sigma ,\mu _{p,t}) \le C \alpha _{\sigma }(Q), \end{aligned}$$

where $C>0$ depends only on *n* and $C_{\sigma }.$

The lemma is not very surprising. The plane $\Lambda (p,t)$ is obtained by locally smoothing $\Gamma _{\mathcal {S}},$ which is composed of pieces of planes that approximate $\partial \Omega .$

### Proof

Thanks to the good approximation properties of the Lipschitz graph ${\mathfrak {b}}(p)$ that we obtain in Sect. 3.4, this lemma can be proved similarly as Lemma 5.22 in [23]. Let $Q\in \mathcal {S},$ $p\in \Pi (\frac{3}{2}B_Q),$ and *t* with $\ell (Q)/4 \le \left| t\right| \le \ell (Q)/2$ be fixed. Denote $r=\left| t\right| .$ By Lemma 3.16, $\alpha _{\sigma }(Q)\le \epsilon _1.$ Since we have chosen $\epsilon _1$ sufficiently small, Lemma 3.13 gives that

5.10 $$\begin{aligned} \sup _{y\in 999\Delta _Q}{{\,\textrm{dist}\,}}(y,P_Q)\le C\epsilon _1^{1/n}\ell (Q)\le 10\ell (Q). \end{aligned}$$

Define a Lipschitz function $\Psi$ by

$$\begin{aligned} \Psi (z):={\left\{ \begin{array}{ll} \frac{1}{4} &{} z\in B(x_Q,100\ell (Q)),\\ \frac{1}{3600\ell (Q)}\left( 10^3\ell (Q)-\left| z-x_Q\right| \right) _+ &{}\text {otherwise}, \end{array}\right. } \end{aligned}$$

where $(f(z))_+:=\max \left\{ 0,f(z)\right\} .$ Then set $f(z)=\Psi (z){{\,\textrm{dist}\,}}(z, P_Q).$ Observe that ${{\,\textrm{supp}\,}}f\subset B(x_Q,10^3\ell (Q)),$ and that $\left| \nabla f(z)\right| \le \Psi (z)+{{\,\textrm{dist}\,}}(z, P_Q)\left| \nabla \Psi \right| \le 1,$ because ${{\,\textrm{dist}\,}}(z,P_Q)\le 10\ell (Q)+10^3\ell (Q)$ by (5.10). Hence $f\in Lip(Q).$ By using successively the facts that $f\ge 0,$ $\int f \, d\mu _Q = 0$ and (3.12), we have that

5.11 $$\begin{aligned} \int _{\Delta (x_Q,100\ell (Q))}{{\,\textrm{dist}\,}}(z, P_Q)d\sigma (z)= & {} 4\int _{\Delta (x_Q,100\ell (Q))}\Psi (z){{\,\textrm{dist}\,}}(z,P_Q)d\sigma (z)\nonumber \\\le & {} 4 \int f\, d\sigma = 4\int f(z)(d\sigma -d\mu _Q)\nonumber \\\le & {} C\ell (Q)^n\alpha _{\sigma }(Q). \end{aligned}$$

Now we estimate the distance from $\Lambda (p,t)$ to $P_Q.$ Write

$$\begin{aligned} {{\,\textrm{dist}\,}}({\mathfrak {b}}^t(p),P_Q)\le \int _{q\in B(p,r)\cap P}\eta _t(p-q){{\,\textrm{dist}\,}}({\mathfrak {b}}(q),P_Q)dq. \end{aligned}$$

Notice that our choice of *p* and *t* ensures that

5.12 $$\begin{aligned} B(p,r)\cap P\subset \Pi (2B_Q). \end{aligned}$$

So we have that

5.13 $$\begin{aligned} {{\,\textrm{dist}\,}}({\mathfrak {b}}^t(p),P_Q)\le & {} r^{1-n}\left\| \eta \right\| _{\infty } \int _{q\in \Pi (2B_Q)}{{\,\textrm{dist}\,}}({\mathfrak {b}}(q),P_Q)dq \nonumber \\\le & {} Cr^{1-n}\left\| \eta \right\| _{\infty }\ell (Q)^n\alpha _{\sigma }(Q) \le C\ell (Q)\alpha _{\sigma }(Q), \end{aligned}$$

where we have used Lemma 3.47. We claim that

5.14 $$\begin{aligned} {{\,\textrm{dist}\,}}(y,P_Q)\le C\alpha _{\sigma }(Q)\left( \left| y-{\mathfrak {b}}^t(p)\right| +\ell (Q)\right) \quad \text {for all }y\in \Lambda (p,t). \end{aligned}$$

Let $y=(q,(q-p) \nabla b^t(p) + b^t(p))\in \Lambda (p,t)$ be fixed. Denote by $\Pi _Q^\bot$ the orthogonal projection on the orthogonal complement of $P_Q.$ Then

5.15 $$\begin{aligned} {{\,\textrm{dist}\,}}(y,P_Q)\le \left| \Pi _Q^\bot \left( y-{\mathfrak {b}}^t(p)\right) \right| +{{\,\textrm{dist}\,}}({\mathfrak {b}}^t(p), P_Q). \end{aligned}$$

Also, $\Pi _Q^\bot (P_Q)$ is a single point $\xi _Q\in \mathbb {R}.$ Denote $v:=y-{\mathfrak {b}}^t(p)=(q-p, (q-p)\nabla b^t(p)).$ Let ${\hat{v}}^i={\hat{v}}^i(p,t)=\partial _{p_i}{\mathfrak {b}}^t(p),$ $i=1,2,\dots ,n-1.$ Then $v=\sum _{i=1}^{n-1}(q_i-p_i){\hat{v}}^i.$ We estimate $\left| \Pi _Q^\bot ({\hat{v}}^i)\right| .$ By definition, we write

$$\begin{aligned} \left| \Pi _Q^\bot ({\hat{v}}^i)\right|= & {} \left| \Pi _Q^\bot (\partial _{p_i}{\mathfrak {b}}^t(p))\right| =\frac{1}{r}\left| \Pi _Q^\bot ((\partial _i\eta )_t*{\mathfrak {b}}(p))\right| \\= & {} \frac{1}{r}\left| \int _{q\in B(p,r)\cap P}(\partial _i\eta )_t(p-q)\Pi _Q^\bot ({\mathfrak {b}}(q))dq\right| \\= & {} \frac{1}{r}\left| \int _{q\in B(p,r)\cap P}(\partial _i\eta )_t(p-q)\left( \Pi _Q^\bot ({\mathfrak {b}}(q))-\xi _Q\right) dq\right| , \end{aligned}$$

where in the last equality we have used that $\int (\partial _i\eta )_t(x) dx=0.$ Notice that $\left| \Pi _Q^\bot (z)-\xi _Q\right| =\left| \Pi _Q^\bot (z)-\Pi _Q^\bot (P_Q)\right| ={{\,\textrm{dist}\,}}(z,P_Q),$ so we have that

$$\begin{aligned} \left| \Pi _Q^\bot ({\hat{v}}^i)\right|\le & {} \frac{1}{r^n}\left\| \partial _i\eta \right\| _{\infty }\int _{q\in B(p,r)\cap P}{{\,\textrm{dist}\,}}({\mathfrak {b}}(q),P_Q)dq\le \frac{C}{r^n}\ell (Q)^n\alpha _{\sigma }(Q)\\\le & {} C\alpha _{\sigma }(Q) \end{aligned}$$

by (5.12) and Lemma 3.47. This gives that

5.16 $$\begin{aligned} \left| \Pi _Q^\bot (v)\right| \le C\alpha _{\sigma }(Q)\left| v\right| . \end{aligned}$$

Then the claim (5.14) follows from (5.15) and (5.13).

Next we compare $c_Q$ (defined in (3.11)) and $\lambda (p,t),$ and claim that

5.17 $$\begin{aligned} \left| \lambda (p,t)-c_Q\right| \le C\alpha _{\sigma }(Q). \end{aligned}$$

We intend to apply (3.12) to the 1-Lipschitz function $|t|\,\theta _{p,t}/\left\| \theta \right\| _{Lip}.$ So we need to check that ${{\,\textrm{supp}\,}}\theta _{p,t}\subset B(x_Q,10^3\ell (Q)).$ By the construction of $\theta _{p,t},$ we have that ${{\,\textrm{supp}\,}}\theta _{p,t}\subset B({\mathfrak {b}}^t(p),r).$ By Lemma 3.40 and the fact that $\epsilon _0$ has been chosen to be small,

5.18 $$\begin{aligned} \left| {\mathfrak {b}}^t(p)-{\mathfrak {b}}(p)\right|= & {} \left| b^t(p)-b(p)\right| =\left| \int \eta _t(q)\left( b(q)-b(p)\right) dq\right| \nonumber \\\le & {} \left\| \nabla b\right\| _{\infty } r\le 2\,\epsilon _0\,r<r. \end{aligned}$$

So $B({\mathfrak {b}}^t(p),r)\subset B({\mathfrak {b}}(p),2r).$ We show that

5.19 $$\begin{aligned} \left| {\mathfrak {b}}(p)-x_Q\right| \le 10\ell (Q). \end{aligned}$$

Then the assumption $r\in [\ell (Q)/4,\ell (Q)/2]$ gives that

$$\begin{aligned} {{\,\textrm{supp}\,}}\theta _{p,t}\subset B({\mathfrak {b}}(p),2r)\subset B(x_Q,10^3\ell (Q)), \end{aligned}$$

as desired. To see (5.19), we recall that $p\in \Pi (\frac{3}{2} B_Q),$ and so $\left| p-\Pi (x_Q)\right| \le 3\ell (Q)/2.$ Let $x\in \partial \Omega$ be a point such that $\left| {\mathfrak {b}}(p)-x\right| ={{\,\textrm{dist}\,}}({\mathfrak {b}}(p),\partial \Omega )\lesssim \epsilon _1\ell (Q),$ where the last inequality is due to Lemma 3.46. Notice that by the definition (3.28), $d(\Pi (x_Q))\le \ell (Q).$ So if $\left| x-x_Q\right| \le 10^{-3}d(\Pi (x_Q)),$ then $\left| x-x_Q\right| \le 10^{-3}\ell (Q),$ and thus

$$\begin{aligned} \left| {\mathfrak {b}}(p)-x_Q\right| \le \left| {\mathfrak {b}}(p)-x\right| +\left| x-x_Q\right| \le C\epsilon _1\ell (Q)+10^{-3}\ell (Q)\le 10\ell (Q), \end{aligned}$$

as desired. If $\left| x-x_Q\right| > 10^{-3}d(\Pi (x_Q)),$ then we can apply (3.29) to get that $\left| \Pi ^\bot (x)-\Pi ^\bot (x_Q)\right| \le 2\epsilon _0\left| \Pi (x)-\Pi (x_Q)\right| .$ By the triangle inequality, $\left| \Pi (x)-\Pi (x_Q)\right| \le \left| \Pi (x)-p\right| +\left| p-\Pi (x_Q)\right| \le \left( C\epsilon _1+\frac{3}{2}\right) \ell (Q)$, and so $\left| \Pi ^\bot (x)-\Pi ^\bot (x_Q)\right| \le 2\epsilon _0\left( \frac{3}{2}+C\epsilon _1\right) \ell (Q).$ Hence, we still have that

$$\begin{aligned} \left| {\mathfrak {b}}(p)-x_Q\right| \le \left| {\mathfrak {b}}(p)-x\right| +\left| x-x_Q\right| \le C\epsilon _1\ell (Q)+2\left( C\epsilon _1+3/2\right) \ell (Q)\le 10\ell (Q), \end{aligned}$$

which completes the proof of (5.19). We have justified that $t\, \theta _{p,t}/\left\| \theta \right\| _{Lip}\in Lip(Q),$ so we can apply (3.12) to this function and obtain that

5.20 $$\begin{aligned} \left| c_\theta r^{n-1}\lambda (p,t)-c_Q\int \theta _{p,t}d\mu _{P_Q}\right| =\left| \int \theta _{p,t}(d\sigma -d\mu _Q)\right| \le C\ell (Q)^{n-1}\alpha _{\sigma }(Q). \nonumber \\ \end{aligned}$$

We now estimate $A_\mu :=c_Q\int \theta _{p,t}(z)d\mu _{P_Q}(z).$ Denote by $\Pi _Q$ the orthogonal projection from $\Lambda (p,t)$ to $P_Q;$ by (5.16) this is an affine bijection, with a constant Jacobian $J_Q$ that satisfies

5.21 $$\begin{aligned} \left| \det (J_Q)-1\right| \le \left| \sqrt{1- C\alpha _{\sigma }(Q)^2}-1\right| \le C\alpha _{\sigma }(Q). \end{aligned}$$

By a change of variables $z=\Pi _Q(y),$ we write

5.22 $$\begin{aligned} A_\mu =c_Q\det (J_Q)\int _{y\in \Lambda (p,t)}\theta _{p,t}\left( \Pi _Q(y)\right) d\mu _{\Lambda (p,t)}(y). \end{aligned}$$

We compare $\int _{y\in \Lambda (p,t)}\theta _{p,t}\left( \Pi _Q(y)\right) d\mu _{\Lambda (p,t)}(y)$ and $\int _{y\in \Lambda (p,t)}\theta _{p,t}(y)d\mu _{\Lambda (p,t)}(y).$ For $y\in \Lambda (p,t),$ $\left| \Pi _Q(y)-y\right| ={{\,\textrm{dist}\,}}(y,P_Q).$ So by (5.14), for $y\in \Lambda (p,t)$

5.23 $$\begin{aligned} \left| \theta _{p,t}\left( \Pi _Q(y)\right) -\theta _{p,t}(y)\right|\le & {} \left\| \theta \right\| _{Lip}r^{-1}\left| \Pi _Q(y)-y\right| \nonumber \\\le & {} C\,r^{-1}\alpha _{\sigma }(Q)\left( \left| y-{\mathfrak {b}}^t(p)\right| +\ell (Q)\right) . \end{aligned}$$

Moreover, the support property of $\theta _{p,t}$ implies that $\left| \theta _{p,t}\left( \Pi _Q(y)\right) -\theta _{p,t}(y)\right|$ is not zero when either $y\in B({\mathfrak {b}}^t(p),r)$ or $\Pi _Q(y)\in B({\mathfrak {b}}^t(p),r).$ By the triangle inequality and (5.14),

$$\begin{aligned} \left| y-{\mathfrak {b}}^t(p)\right| \le \left| \Pi _Q(y)-{\mathfrak {b}}^t(p)\right| +C\alpha _{\sigma }(Q)\left( \left| y-{\mathfrak {b}}^t(p)\right| +\ell (Q)\right) . \end{aligned}$$

Since $\alpha _{\sigma }(Q)\le \epsilon _1$ is sufficiently small, we get that when $\Pi _Q(y)\in B({\mathfrak {b}}^t(p),r),$ $\left| y-{\mathfrak {b}}^t(p)\right| \le 2r+\mathcal {C}\epsilon _1\ell (Q)\le 3\ell (Q)$. So by (5.23) and the fact that ${{\,\textrm{supp}\,}}\theta _{p,t}\subset B({\mathfrak {b}}^t(p),r),$

5.24 $$\begin{aligned} \left| \int _{y\in \Lambda (p,t)}\left( \theta _{p,t}\left( \Pi _Q(y)\right) -\theta _{p,t}(y)\right) d\mu _{\Lambda (p,t)}(y)\right| \le C\,r^{n-1}\alpha _{\sigma }(Q). \end{aligned}$$

Hence we have $\left| A_\mu -c_Q\det (J_Q)c_\theta \, r^{n-1}\right| \le C\,c_Q\,r^{n-1}\alpha _{\sigma }(Q)$ by definition of $c_\theta .$ By the triangle inequality, (5.21), and the fact that $r \approx \ell (Q),$

$$\begin{aligned} \left| A_\mu -c_Q\,c_\theta \,r^{n-1}\right| \le C\,c_Q\alpha _{\sigma }(Q)\ell (Q)^{n-1}. \end{aligned}$$

By this, the triangle inequality and (5.20),

5.25 $$\begin{aligned} c_\theta \,r^{n-1}\left| \lambda (p,t)-c_Q\right| =\left| c_\theta r^{n-1}\lambda (p,t)-c_Q\,c_\theta \,r^{n-1}\right| \le C(1+c_Q)\alpha _{\sigma }(Q)\ell (Q)^{n-1},\nonumber \\ \end{aligned}$$

which implies that $\left| \lambda (p,t)-c_Q\right| \le \frac{1}{2}(1+c_Q)$ because $\alpha _{\sigma }(Q)$ is sufficiently small. But we know $\lambda (p,t)\approx 1$ by (5.6), so $c_Q\approx 1$ and then (5.25) yields the desired estimate (5.17).

Finally, we are ready to show that

5.26 $$\begin{aligned} {{\,\textrm{dist}\,}}_Q(\mu _Q,\mu _{p,t})\le C\alpha _{\sigma }(Q). \end{aligned}$$

Let $f\in Lip(Q).$ We have that

$$\begin{aligned} \int f(z)d\mu _Q(z)= & {} c_Q\int _{P_Q}f(z)d\mu _{P_Q}(z)\\= & {} c_Q\det (J_Q)\int _{\Lambda (p,t)}f\left( \Pi _Q(y)\right) d\mu _{\Lambda (p,t)}(y). \end{aligned}$$

An argument similar to the one for (5.24) gives that

$$\begin{aligned} \left| \int _{\Lambda (p,t)}\left( f\left( \Pi _Q(y)\right) -f(y)\right) d\mu _{\Lambda (p,t)}(y)\right| \le C\alpha _{\sigma }(Q)\ell (Q)^n. \end{aligned}$$

So

$$\begin{aligned}{} & {} \left| \int f\,d\mu _Q-\lambda (p,t)\int f\,d\mu _{\Lambda (p,t)}\right| \\{} & {} \quad \le \left| \int f\,d\mu _Q-c_Q\int f\,d\mu _{\Lambda (p,t)}\right| +\left| \lambda (p,t)-c_Q\right| \left| \int f\,d\mu _{\Lambda (p,t)}\right| \\{} & {} \quad \le Cc_Q\det (J_Q)\alpha _{\sigma }(Q)\ell (Q)^n +c_Q\left| \det (J_Q)-1\right| \left| \int f\,d\mu _{\Lambda (p,t)}\right| \\{} & {} \qquad +\left| \lambda (p,t)-c_Q\right| \left| \int f\,d\mu _{\Lambda (p,t)}\right| . \end{aligned}$$

By (5.21), (5.17), and $$c_Q\approx 1,$$

5.27 $$\begin{aligned} \left| \int f\,d\mu _Q-\lambda (p,t)\int f\,d\mu _{\Lambda (p,t)}\right| \le C\alpha _{\sigma }(Q)\ell (Q)^n, \end{aligned}$$

which proves (5.26). Now (5.9) follows from (5.26) and (3.12). $\square$

We want to use the flat measures $\mu _{p,t}$ to estimate the smooth distance $D_{\beta }$ introduced in (1.3). But before that, we shall need to introduce

5.28 $$\begin{aligned} \alpha _{\sigma }(Q,k) := \alpha _{\sigma }(Q^{(k)}), \end{aligned}$$

where $Q^{(k)}$ is the unique ancestor of *Q* such that $\ell (Q^{(k)}) = 2^k\ell (Q),$ and then for $\beta >0,$

5.29 $$\begin{aligned} \alpha _{\sigma ,\beta }(Q) := \sum _{k\in \mathbb {N}} 2^{-k\beta } \alpha _{\sigma }(Q,k). \end{aligned}$$

The collection $\{\alpha _{\sigma ,\beta }(Q)\}_Q$ is nice, because we have

5.30 $$\begin{aligned} \alpha _{\sigma ,\beta }(Q^*) \approx \alpha _{\sigma ,\beta }(Q) \end{aligned}$$

whenever $Q\in {\mathbb {D}}_{\partial \Omega }$ and $Q^*$ is the parent of *Q*,  a property which is not satisfied by the $\{\alpha _{\sigma }(Q)\}_Q.$ And of course the $\alpha _{\sigma ,\beta }(Q)$’s still satisfies the Carleson packing condition.

### Lemma 35

Let $\partial \Omega$ be uniformly rectifiable and $\sigma$ be an Ahlfors regular measure satisfying (1.1). There exists a constant $C_{\sigma ,\beta }$ that depends only on the constant in (3.9),  the Ahlfors regular constant $C_{\sigma },$ and $\beta$ such that,  for any $Q_0 \in {\mathbb {D}}_{\partial \Omega },$

5.32 $$\begin{aligned} \sum _{Q\in {\mathbb {D}}_{\partial \Omega }(Q_0)} |\alpha _{\sigma ,\beta }(Q)|^2 \sigma (Q) \le C_{\sigma ,\beta } \sigma (Q_0). \end{aligned}$$

### Proof

Same as Lemma 5.89 in [23]. $\square$

The quantities $\alpha _{\sigma ,\beta }$ are convenient, because we can now obtain an analogue of Lemma 5.8 where we don’t need to pay too much attention to the choices of *p* and *t*. $\square$

### Lemma 36

Let $\beta >0$ and $K\ge 1.$ For $Q\in \mathcal {S},$ $p\in \Pi ( K B_Q),$ and $\ell (Q)/K \le \left| t\right| \le K\ell (Q),$ we have

5.34 $$\begin{aligned} {{\,\textrm{dist}\,}}_Q(\sigma ,\mu _{p,t}) \le C_{\beta ,K} \alpha _{\sigma ,\beta }(Q), \end{aligned}$$

where $C_{\beta ,K}>0$ depends only on *n*,  $C_{\sigma },$ $\beta ,$ and *K*.

### Proof

First, we prove that when $p\in \Pi (\frac{3}{2} B_Q)$ and $\ell (Q)/K \le |t| \le \ell (Q)/2,$ we have

5.35 $$\begin{aligned} {{\,\textrm{dist}\,}}_Q(\sigma ,\mu _{p,t}) \le C_K \alpha _{\sigma }(Q). \end{aligned}$$

We set $t_j = 2^{-2-j}\ell (Q).$ We also take a dyadic cube $Q' \subset Q$ such that $\ell (Q')/4 \le |t| \le \ell (Q')/2,$ and then we pick $p_0\in \Pi (\frac{3}{2} B_{Q'}).$ By Lemma 5.8, we have that

$$\begin{aligned} {{\,\textrm{dist}\,}}_{Q}(\sigma ,\mu _{p,t_0}) + {{\,\textrm{dist}\,}}_{Q}(\sigma ,\mu _{p_0,t_0}) \lesssim \alpha _{\sigma }(Q), \end{aligned}$$

so ${{\,\textrm{dist}\,}}_{Q}(\mu _{p,t_0},\mu _{p_0,t_0}) \lesssim \alpha _{\sigma }(Q)$ too. Consequently, the claim (5.35) is reduced to

5.36 $$\begin{aligned} {{\,\textrm{dist}\,}}_{Q}(\mu _{p_0,t},\mu _{p_0,t_0}) \le C_K \alpha _{\sigma }(Q). \end{aligned}$$

For this latter bound, we decompose

5.37 $$\begin{aligned} {{\,\textrm{dist}\,}}_{Q}(\mu _{p_0,t},\mu _{p_0,t_0}) \le {{\,\textrm{dist}\,}}_{Q}(\mu _{p_0,t},\mu _{p_0,t_k}) + \sum _{j=0}^{k-1} {{\,\textrm{dist}\,}}_{Q}(\mu _{p_0,t_j},\mu _{p_0,t_{j+1}}), \nonumber \\ \end{aligned}$$

where *k* is chosen so that $t_k = \ell (Q')/2,$ and $k\le 1+\log _2(K)$ is bounded by *K*. We look at ${{\,\textrm{dist}\,}}_{Q}(\mu _{p_0,t},\mu _{p_0,t_k}),$ but since we are dealing with two flat measures that intersect $B_{Q'},$ Lemma A.5 in [31] shows that

5.38 $$\begin{aligned} {{\,\textrm{dist}\,}}_{Q}(\mu _{p_0,t},\mu _{p_0,t_k}) \lesssim {{\,\textrm{dist}\,}}_{Q'} (\mu _{p_0,t},\mu _{p_0,t_k}) \end{aligned}$$

and then Lemma 5.8 and the fact that $\ell (Q') \approx _K \ell (Q)$ entail that

5.39 $$\begin{aligned} {{\,\textrm{dist}\,}}_{Q}(\mu _{p_0,t},\mu _{p_0,t_k}) \lesssim {{\,\textrm{dist}\,}}_{Q'} (\mu _{p_0,t},\sigma ) + {{\,\textrm{dist}\,}}_{Q'} (\sigma ,\mu _{p_0,t_k}) \lesssim \alpha _{\sigma }(Q') \le C_K \alpha _{\sigma }(Q). \nonumber \\ \end{aligned}$$

A similar reasoning gives that

5.40 $$\begin{aligned} {{\,\textrm{dist}\,}}_{Q}(\mu _{p_0,t_j},\mu _{p_0,t_{j+1}}) \lesssim C_K \alpha _{\sigma }(Q) \end{aligned}$$

whenever $0\le j \le k-1.$ The combination of (5.37), (5.39), and (5.40) shows the claim (5.36) and thus (5.35).

In the general case, we pick the smallest ancestor $Q^*$ of *Q* such that $p\in \Pi (\frac{3}{2}B_{Q^*})$ and $|t| \le \ell (Q^*)/2,$ and we apply (5.35) to get

$$\begin{aligned} {{\,\textrm{dist}\,}}_Q(\sigma ,\mu _{p,t}) \le C_K \alpha _{\sigma }(Q^*). \end{aligned}$$

The lemma follows then by simply observing that $\alpha _{\sigma }(Q^*) \lesssim \alpha _{\sigma ,\beta }(Q).$ $\square$

We need the constant

5.41 $$\begin{aligned} c_{\beta } := \int _{\mathbb {R}^{n-1}} (1+|p|^2)^{-\frac{d+\beta }{2}} dy \end{aligned}$$

and the unit vector $N_{p,t}$ defined as the vector

5.42 $$\begin{aligned} N_{p,t}(X) := [\nabla {{\,\textrm{dist}\,}}(.,\Lambda (p,t))](X) \end{aligned}$$

which is of course constant on the two connected components of $\mathbb {R}^n {\setminus }\Lambda (p,t).$ We are now ready to compare $D_{\beta }$ with the distance to $\Lambda (p,t).$

### Lemma 37

Let $Q\in \mathcal {S},$ $X\in W_{\Omega }(Q),$ $p\in \Pi (2^5Q)$ and $2^{-5}\ell (Q) \le |t| \le 2^5 \ell (Q).$ We have

5.44 $$\begin{aligned} |D^{-\beta }_{\beta }(X) - c_{\beta } \lambda (p,t) {{\,\textrm{dist}\,}}(X,\Lambda (p,t))^{-\beta }| \le C \ell (Q)^{-\beta } \alpha _{\sigma ,\beta }(Q), \end{aligned}$$

and

5.45 $$\begin{aligned}{} & {} |\nabla [D^{-\beta }_{\beta }](X) + \beta c_{\beta } \lambda (p,t) {{\,\textrm{dist}\,}}(X,\Lambda (p,t))^{-\beta -1} N_{p,t}(X)| \nonumber \\{} & {} \quad \le C \ell (Q)^{-\beta -1} \alpha _{\sigma ,\beta +1}(Q), \end{aligned}$$

where the constant $C>0$ depends only $C_{\sigma }$ and $\beta .$

### Proof

Denote $r=\left| t\right| ,$ and $d=n-1.$ By the definition of $W_\Omega (Q),$ ${{\,\textrm{dist}\,}}(X,\partial \Omega )>\ell (Q)/2$ and $X\in 2B_Q.$ We show that in addition,

5.46 $$\begin{aligned} X\in B({\mathfrak {b}}^t(p),2^6\ell (Q)), \end{aligned}$$

and

5.47 $$\begin{aligned} {{\,\textrm{dist}\,}}(X,\Lambda (p,t)\cup \partial \Omega )>\frac{\ell (Q)}{20}. \end{aligned}$$

Since $X\in 2B_Q,$ $\left| \Pi (X)-p\right| \le (2^5+2) \ell (Q).$ Then $\left| {\mathfrak {b}}(\Pi (X))-{\mathfrak {b}}(p)\right| \le (1+2\epsilon _0)(2^5+2)\ell (Q)$ because ${\mathfrak {b}}$ is the graph of a $2\epsilon _0$-Lipschitz function. Write

$$\begin{aligned} \left| X-{\mathfrak {b}}^t(p)\right| \le \left| X-{\mathfrak {b}}(\Pi (X))\right| +\left| {\mathfrak {b}}(\Pi (X))-{\mathfrak {b}}(p)\right| +\left| {\mathfrak {b}}(p)-{\mathfrak {b}}^t(p)\right| , \end{aligned}$$

then use (4.14) and (5.18) to get

$$\begin{aligned} \left| X-{\mathfrak {b}}^t(p)\right| \le (1+2\epsilon _0)\left( \delta (X)+(2^5+2)\ell (Q)\right) +2\epsilon _0r\le 2^6\ell (Q), \end{aligned}$$

and thus (5.46) follows. In order to see (5.47), we only need to show that ${{\,\textrm{dist}\,}}(X,\Lambda (p,t))>\frac{\ell (Q)}{20}.$ Notice that $(\nabla b^t(p),-1)$ is a normal vector of the plane $\Lambda (p,t),$ and that ${\mathfrak {b}}^t(p)\in \Lambda (p,t).$ So

5.48 $$\begin{aligned} {{\,\textrm{dist}\,}}(X,\Lambda (p,t))= & {} \frac{\left| \left( X-{\mathfrak {b}}^t(p)\right) \cdot \left( \nabla b^t(p),-1\right) \right| }{\left| (\nabla b^t(p),1)\right| }\nonumber \\= & {} \frac{\left| (\Pi (X)-p)\cdot \nabla b^t(p)+\left( b^t(p)-\Pi ^\bot (X)\right) \right| }{\sqrt{\left| \nabla b^t(p)\right| ^2+1}}\nonumber \\\ge & {} \frac{1}{2}\left( \left| \Pi ^\bot (X)-b^t(p)\right| -\left| (\Pi (X)-p)\cdot \nabla b^t(p)\right| \right) \nonumber \\\ge & {} \frac{1}{2}\left| \Pi ^\bot (X)-b^t(p)\right| -C\,2^5\ell (Q)\epsilon _0, \end{aligned}$$

by $\left\| \nabla b^t\right\| _{\infty }\le C\epsilon _0$ (see (6.2)). We have $\left| b(\Pi (X))-b(p)\right| \le 2\epsilon _0\left| \Pi (X)-p\right| \le 2^6\epsilon _0\ell (Q),$ and $\left| \Pi ^\bot (X)-b(\Pi (X))\right| \ge {{\,\textrm{dist}\,}}(X,\Gamma _S)\ge \frac{\delta (X)}{1+3\epsilon _0}\ge \frac{\ell (Q)}{2(1+3\epsilon _0)}$ by (4.15). So

$$\begin{aligned} \left| \Pi ^\bot (X)-b^t(p)\right|\ge & {} \left| \Pi ^\bot (X)-b(\Pi (X))\right| -\left| b(\Pi (X))-b(p)\right| -\left| b^t(p)-b(p)\right| \\\ge & {} \frac{\ell (Q)}{5} \end{aligned}$$

by (5.18). Then ${{\,\textrm{dist}\,}}(X,\Lambda (p,t))>\frac{\ell (Q)}{20}$ follows from this and (5.48).

Now we prove (5.44). We intend to cut $D_{\beta }^{-\beta }=\int _{\partial \Omega }\left| X-y\right| ^{-d-\beta }d\sigma (y)$ into pieces. So we introduce a cut-off function $\theta _0\in C_c^\infty (B(0,r/2)),$ which is radial, ${\mathbb {1}}_{B(0,r/4)}\le \theta _0\le {\mathbb {1}}_{B(0,r/2)},$ and $\left| \nabla \theta _0\right| \le 2r.$ Then we set $\theta _k(y):=\theta _0(2^{-k}y)-\theta _0(-2^{-k+1}y)$ for $k\ge 1$ and $y\in \mathbb R^n,$ and define $\widetilde{\theta }_k(y)=\theta _k(y-{\mathfrak {b}}^t(p))$ for $k\in \mathbb {N}.$ Denote $B_k=B({\mathfrak {b}}^t(p),2^{k-1}r).$ We have that ${{\,\textrm{supp}\,}}\widetilde{\theta }_0\subset B_0,$ ${{\,\textrm{supp}\,}}\widetilde{\theta }_k\subset B_k{\setminus } B_{k-2}$ for $k\ge 1,$ and that

$$\begin{aligned} \sum _{k\in \mathbb {N}}\widetilde{\theta }_k=1. \end{aligned}$$

Now we can write

$$\begin{aligned} D_{\beta }(X)^{-\beta }=\sum _{k\in \mathbb {N}}\int _{\partial \Omega }\left| X-y\right| ^{-d-\beta }\widetilde{\theta }_k(y)d\sigma (y)=:\sum _{k\in \mathbb {N}}\int _{\partial \Omega } f_k(y)d\sigma (y), \end{aligned}$$

with $f_k(y)=\left| X-y\right| ^{-d-\beta }\widetilde{\theta }_k(y).$ We intend to compare $\int f_k(y)d\sigma (y)$ and $\int f_k(y)d\mu _{p,t}(y).$ Both integrals are well-defined because of (5.47). Observe that

$$\begin{aligned} \sum _{k\in \mathbb {N}}\int f_k(y)d\mu _{p,t}(y)= & {} \lambda (p,t)\int _{\Lambda (p,t)}\left| X-y\right| ^{-d-\beta }d\mu _{\Lambda (p,t)}(y)\\= & {} \lambda (p,t)\int _{\mathbb {R}^d}\left( {{\,\textrm{dist}\,}}(X,\Lambda (p,t))^2+\left| y\right| ^2\right) ^{-(d+\beta )/2}dy\\= & {} \lambda (p,t)c_{\beta }{{\,\textrm{dist}\,}}(X,\Lambda (p,t))^{-\beta } \end{aligned}$$

by a change of variables. So

5.49 $$\begin{aligned} D^{-\beta }_{\beta }(X) - c_{\beta } \lambda (p,t){{\,\textrm{dist}\,}}(X,\Lambda (p,t))^{-\beta }=\sum _{k\in \mathbb {N}}\int f_k\,(d\sigma -d\mu _{p,t}). \end{aligned}$$

We are interested in the Lipschitz properties of $f_k$ because we intend to use Wasserstein distances. We claim that

5.50 $$\begin{aligned} \left| X-y\right| \ge c2^kr \quad \text {when }y\in \partial \Omega \cup \Lambda (p,t) \text { is such that } \widetilde{\theta }_k(y)\ne 0, \end{aligned}$$

where $c=10^{-1}2^{-21}.$ In fact, by (5.46) and the support properties of $\widetilde{\theta }_k,$ if $k\ge 15,$ then

$$\begin{aligned} \left| X-y\right| \ge 2^{k-3}r-2^6\ell (Q)\ge (2^{k-3}-2^{11})r\ge 2^{k-4}r \quad \text {for }y\in {{\,\textrm{supp}\,}}\widetilde{\theta }_k. \end{aligned}$$

If $0\le k<15,$ then by (5.47), for $y\in \partial \Omega \cup \Lambda (p,t),$

$$\begin{aligned} \left| X-y\right| \ge {{\,\textrm{dist}\,}}(X,\partial \Omega \cup \Lambda (p,t))\ge \frac{\ell (Q)}{20}\ge \frac{2^{-6}r}{10}\ge \frac{2^{-21}}{10}2^kr. \end{aligned}$$

So (5.50) is justified. But $f_k$ is not a Lipschitz function in $\mathbb R^n$ because *y* can get arbitrarily close to *X* when *k* is small. Set

$$\begin{aligned} \widetilde{f}_k(y):=\max \left\{ \left| X-y\right| ,c\,2^kr\right\} ^{-d-\beta }\widetilde{\theta }_k(y). \end{aligned}$$

Then by (5.50), $\widetilde{f}_k(y)=f_k(y)$ for $y\in \partial \Omega \cup \Lambda (p,t),$ and therefore,

5.51 $$\begin{aligned} \int f_k\,(d\sigma -d\mu _{p,t})=\int \widetilde{f}_k\,(d\sigma -d\mu _{p,t}). \end{aligned}$$

The good thing about $\widetilde{f}_k$ is that it is Lipschitz. A direct computation shows that $\left\| \widetilde{f}_k\right\| _{\infty }\le C\left( 2^kr\right) ^{-d-\beta },$ and $\left\| \nabla \widetilde{f}_k\right\| _{\infty }\le C(2^kr)^{-d-\beta -1}.$ Moreover, $\widetilde{f}_k$ is supported on $B({\mathfrak {b}}^t(p),2^{k-1}r),$ which is contained in $B(x_{Q^{(k)}},10^3\ell (Q^{(k)})).$ To see this, one can use (5.18), (5.19), and $\left| x_Q-x_{Q^{(k)}}\right| \le 2^{k-1}\ell (Q)$ to get that

$$\begin{aligned} \left| {\mathfrak {b}}^t(p)-x_{Q^{(k)}}\right|\le & {} \left( 2\epsilon _0+2^{k-1}\right) r+10\,2^5\ell (Q)+2^{k-1}\ell (Q) \\\le & {} 2^5(2^k+11)\ell (Q)\le 10^32^k\ell (Q). \end{aligned}$$

Write

$$\begin{aligned} \int \widetilde{f}_k\,(d\sigma -d\mu _{p,t})= & {} \int \widetilde{f}_k\,(d\sigma -d\mu _{Q^{(k)}})+\int \widetilde{f}_k\,(d\mu _Q-d\mu _{p,t}) \\{} & {} +\sum _{j=1}^k\int \widetilde{f}_k\,(d\mu _{Q^{(j)}}-d\mu _{Q^{(j-1)}}) =:I+II+\sum _{j=1}^kIII_j. \end{aligned}$$

By the definition (3.11) of $\mu _{Q^{(k)}}$ and properties of $\widetilde{f}_k,$ $\left| I\right| \le C\left( 2^kr\right) ^{-\beta }\alpha _{\sigma }(Q,k)$. We then have $|II| \le \left( 2^kr\right) ^{-\beta } {{\,\textrm{dist}\,}}_{Q^{(k)}} (\mu _Q , \mu _{p,t}),$ but because we are looking at the Wasserstein distance between two flat measures whose supports intersect $10B_Q,$ Lemma A.5 in [31] shows that

$$\begin{aligned}{{\,\textrm{dist}\,}}_{Q^{(k)}} (\mu _Q , \mu _{p,t}) \lesssim {{\,\textrm{dist}\,}}_{Q} (\mu _Q , \mu _{p,t})\end{aligned}$$

and thus

$$\begin{aligned} |II|\lesssim & {} \left( 2^kr\right) ^{-\beta } {{\,\textrm{dist}\,}}_{Q} (\mu _Q , \mu _{p,t}) \le \left( 2^kr\right) ^{-\beta } \Big ( {{\,\textrm{dist}\,}}_{Q} (\mu _Q, \sigma ) + {{\,\textrm{dist}\,}}_Q( \sigma , \mu _{p,t}) \Big ) \\\lesssim & {} \left( 2^kr\right) ^{-\beta } \alpha _{\sigma ,\beta }(Q) \end{aligned}$$

by Lemma 5.33. The terms $III_j$ can be bounded by a Wasserstein distance between planes, and similarly to *II*,  we get

$$\begin{aligned} |III_j|\lesssim & {} \left( 2^kr\right) ^{-\beta } {{\,\textrm{dist}\,}}_{Q^{(k)}} (\mu _{Q^{(j)}} , \mu _{Q^{(j-1)}}) \\\lesssim & {} \left( 2^kr\right) ^{-\beta } {{\,\textrm{dist}\,}}_{Q^{(j)}} (\mu _{Q^{(j)}} , \mu _{Q^{(j-1)}}) \lesssim \left( 2^kr\right) ^{-\beta } \alpha _{\sigma }(Q,j). \end{aligned}$$

Altogether, we obtain that

$$\begin{aligned} \left| \int \widetilde{f}_k\,(d\sigma -d\mu _{p,t})\right| \le C\left( 2^kr\right) ^{-\beta } \left( \alpha _{\sigma ,\beta }(Q) + \sum _{j=0}^k\alpha _{\sigma }(Q,j) \right) . \end{aligned}$$

Then by (5.51) and (5.49),

$$\begin{aligned}{} & {} \left| D^{-\beta }_{\beta }(X) - c_{\beta } \lambda (p,t){{\,\textrm{dist}\,}}(X,\Lambda (p,t))^{-\beta }\right| \\{} & {} \quad \le C\sum _{k\in \mathbb {N}}\left( 2^kr\right) ^{-\beta }\left( \alpha _{\sigma ,\beta }(Q) + \sum _{j=0}^k\alpha _{\sigma }(Q,j) \right) \le C\ell (Q)^{-\beta }\alpha _{\sigma ,\beta }(Q), \end{aligned}$$

which is (5.44).

We claim that (5.45) can be established similarly to (5.44) as long as one expresses the left-hand side of (5.45) appropriately. A direct computation shows that

$$\begin{aligned} \nabla (D_{\beta }^{-\beta })(X)=-(d+\beta )\int \left| X-y\right| ^{-d-\beta -2}(X-y)d\sigma (y). \end{aligned}$$

On the other hand,

$$\begin{aligned}{} & {} \int \left| X-y\right| ^{-d-\beta -2}(X-y)d\mu _{\Lambda (p,t)}(y) \\{} & {} \quad = N_{p,t}(X)\int \left| X-y\right| ^{-d-\beta -2}(X-y)\cdot N_{p,t}(X)d\mu _{\Lambda (p,t)}(y)\\{} & {} \quad =N_{p,t}(X)\int \left| X-y\right| ^{-d-\beta -2}{{\,\textrm{dist}\,}}(X,\Lambda (p,t))d\mu _{\Lambda (p,t)}(y) \\{} & {} \quad =c_{\beta +2}{{\,\textrm{dist}\,}}(X,\Lambda (p,t))^{-\beta -1}N_{p,t}(X). \end{aligned}$$

By [31] (3.30), $(\beta +d)c_{\beta +2}=\beta c_{\beta }$ for all $\beta >0.$ Hence

$$\begin{aligned}{} & {} \nabla [D^{-\beta }_{\beta }](X) + \beta c_{\beta } \lambda (p,t) {{\,\textrm{dist}\,}}(X,\Lambda (p,t))^{-\beta -1} N_{p,t}(X)\\{} & {} \quad =-(d+\beta )\int \left| X-y\right| ^{-d-\beta -2}(X-y)\left( d\sigma (y)-d\mu _{p,t}(y)\right) . \end{aligned}$$

Now we set $f_k'(y)=\left| X-y\right| ^{-d-\beta -2}(X-y).$ Using (5.46) and (5.47), we can see that $f_k'$ is Lipschitz on $\partial \Omega \cup \Lambda (p,t).$ Then we can play with measures as before to obtain (5.45). $\square$

### Corollary 38

Let $Q\in \mathcal {S},$ $X\in W_{\Omega }(Q),$ $p\in \Pi (2^5Q)$ and $2^{-5}\ell (Q) \le |t| \le 2^5 \ell (Q).$ We have

5.53 $$\begin{aligned} \left| \dfrac{\nabla D_{\beta }(X)}{D_{\beta }(X)} - \dfrac{N_{p,t}(X)}{{{\,\textrm{dist}\,}}(X,\Lambda (p,t))}\right| \le C \ell (Q)^{-1} \alpha _{\sigma ,\beta }(Q), \end{aligned}$$

where the constant $C>0$ still depends only $C_{\sigma }$ and $\beta .$

### Proof

To lighten the notation, we denote by ${\mathcal {O}}_{CM}$ any quantity such that

$$\begin{aligned} |{\mathcal {O}}_{CM}| \le C \alpha _{\sigma ,\beta }(Q) \end{aligned}$$

for some constant *C*. Then by (5.45),

$$\begin{aligned} \frac{\nabla D_{\beta }(X)}{D_{\beta }(X)}= & {} - \frac{1}{\beta } \frac{\nabla [D_{\beta }^{-\beta }](X)}{D_{\beta }^{-\beta }(X)} \\= & {} - \frac{1}{\beta }\left( \frac{-\beta c_{\beta } \lambda (p,t) {{\,\textrm{dist}\,}}(X,\Lambda (p,t))^{-\beta -1}N_{p,t}(X)}{D_{\beta }^{-\beta }(X)} + \frac{\ell (Q)^{-\beta -1}{\mathcal {O}}_{CM}}{D_{\beta }^{-\beta }(X)}\right) . \end{aligned}$$

Using $D_{\beta }(X)\approx \delta (X)\approx \ell (Q)$ and (5.44), we can further write the above as

$$\begin{aligned} \frac{\nabla D_{\beta }(X)}{D_{\beta }(X)} = \dfrac{N_{p,t}(X)}{{{\,\textrm{dist}\,}}(X,\Lambda (p,t))} + \ell (Q)^{-1} {\mathcal {O}}_{CM}, \end{aligned}$$

which implies the corollary. $\square$

## The bi-Lipschitz change of variable $\rho _{\mathcal {S}}$

The results in this section are similar, identical, or often even easier than the ones found in Sections 2, 3, and 4 of [23]. Many proofs will only be sketched and we will refer to the corresponding result in [23] for details.

As in the previous sections, we take $0<\epsilon _0 \ll \epsilon _1 \ll 1$ and we use Lemma 3.16 with such $\epsilon _0,\epsilon _1$ to obtain a collection ${\mathfrak {S}}$ of coherent regimes. We take then $\mathcal {S}$ that either belongs to ${\mathfrak {S}},$ or is a coherent regime included in an element of ${\mathfrak {S}}.$ We keep the notations introduced in Sects. 3, 4, and 5.

### Construction of $\rho _{\mathcal {S}}$

In this section, the gradients are column vectors. The other notation is fairly transparent. A hyperplane *P* is equipped with an orthonormal basis and $\nabla _p$ correspond to the vector of the derivatives in each coordinate of *p* in this basis; $\partial _t$ or $\partial _s$ are the derivatives with respect to *t* or *s*,  that are always explicitly written; $\nabla _{p,t}$ or $\nabla _{p,s}$ are the gradients in $\mathbb {R}^n$ seen as $P \times P^\bot .$

#### Lemma 39

The quantities $\nabla _{p,t} b^t$ and $t \nabla _{p,t} \nabla _p b^t$ are bounded,  that is,  for any $t \ne 0$ and any $p_0\in P,$

6.2 $$\begin{aligned} |\nabla _{p,t} b^t| + |t \nabla _{p,t} \nabla _p b^t| \le C\epsilon _0. \end{aligned}$$

In addition,  $|\partial _{t} b^t| + |t \nabla _{p,t} \nabla _p b^t| \in CM_{P\times (P^\bot {\setminus }\{0\})},$ that is,  for any $r>0$ and any $p_0\in P,$

6.3 $$\begin{aligned} \iint _{B(p_0,r)} \Big ( |\partial _t b^t(p)|^2 + |t \nabla _{p,t} \nabla _p b^t(p)|^2 \Big ) \frac{dt}{t} \, dp \le C \epsilon _0^2 r^{n-1}. \end{aligned}$$

In both cases,  the constant $C>0$ depends only on *n* (and $\eta )$.

#### Proof

The result is well-known and fairly easy. The boundedness is proven in Lemma 3.17 of [23], while the Carleson bound is established in Lemma 4.11 in [23] (which is itself a simple application of the Littlewood–Paley theory found in [49, Section I.6.3] to the bounded function $\nabla b)$. $\square$

Observe that the convention that we established shows that $(\nabla _p b^t(p))^T$ is an $(n-1)$-dimensional horizontal vector. We define the map $\rho : P\times P^\bot \rightarrow P\times P^\bot$ as

6.4 $$\begin{aligned} \rho _{\mathcal {S}}(p,t) := (p - t(\nabla b^t(p))^T, t + b^t(p)) \end{aligned}$$

if $t\ne 0$ and $\rho _{\mathcal {S}}(p,0) = {\mathfrak {b}}(p).$ Because the codimension of our boundary is 1 in our paper, contrary to [23] which stands in the context of domains with higher codimensional boundaries, our map is way easier than the one found in [23]. However, the present mapping is still different from the one found in [44], and has the same weak and strong features as the change of variable in [23]. Note that the $i^{th}$ coordinate of $\rho _{\mathcal {S}},$ $1\le i \le n-1,$ is

6.5 $$\begin{aligned} \rho _{\mathcal {S}}^i(p,t) := p_i - t\partial _{p_i} b^t(p). \end{aligned}$$

Note that $\rho _{\mathcal {S}}$ is continuous on $P\times P^\bot = \mathbb {R}^n,$ because both $t\nabla b^t$ and $b^t-b$ converges (uniformly in $p \in P)$ to 0 as $t\rightarrow 0.$ The map $\rho _{\mathcal {S}}$ is $C^\infty$ on $\mathbb {R}^n {\setminus } P,$ and we compute the Jacobian ${{\,\textrm{Jac}\,}}$ of $\rho _{\mathcal {S}}$ which is

6.6 $$\begin{aligned} {{\,\textrm{Jac}\,}}(p,t) = \begin{pmatrix} I - t\partial _{p_i} \partial _{p_j} b^t(p) &{} \partial _{p_i} b^t(p) \\ - t \partial _t \partial _{p_j} b^t(p) - \partial _{p_j} b^t(p) &{} 1 + \partial _t b^t(p) \end{pmatrix}, \end{aligned}$$

where *i* and *j* refer to respectively the line and the column of the matrix. We define the approximation of the Jacobian ${{\,\textrm{Jac}\,}}$ as

6.7 $$\begin{aligned} J = \begin{pmatrix} I &{} \partial _{p_i} b^t(p) \\ - \partial _{p_j} b^t(p) &{} 1 \end{pmatrix} = \begin{pmatrix} I &{} \nabla _p b^t(p) \\ - (\nabla _p b^t(p))^T &{} 1 \end{pmatrix}. \end{aligned}$$

$$\square $$

#### Lemma 40

We have the following pointwise bounds : 

(i) $$\displaystyle \Vert J-I\Vert \lesssim |\nabla _p b^t| \lesssim \epsilon _0,$$
(ii) $$\displaystyle \Vert {{\,\textrm{Jac}\,}}- J\Vert \lesssim |\partial _t b^t| + |t\nabla _{p,t} \nabla _p b^t| \lesssim \epsilon _0,$$
(iii) $$\displaystyle |\det (J)-1| \lesssim |\nabla _p b^t| \lesssim \epsilon _0,$$
(iv) $$\displaystyle |\det ({{\,\textrm{Jac}\,}}) - \det (J)| \lesssim |\partial _t b^t| + |t\nabla _{p,t} \nabla _p b^t|,$$
(v) $$\displaystyle \Vert ({{\,\textrm{Jac}\,}})^{-1} - J^{-1}\Vert \lesssim |\partial _t b| + |t\nabla _{p,t} \nabla _p b^t|,$$
(vi) $$\displaystyle |\nabla _{p,t} \det (J)| + \Vert |\nabla _{p,t} J^{-1}|\Vert \lesssim |\nabla _{p,t} \nabla _p b^t|.$$

In each estimate,  the constants depend only on *n* and $\eta .$

#### Proof

Only a rapid proof is provided, and details are carried out in the proof of Lemmas 3.26, 4.12, 4.13, and 4.15 in [23].

The items (i) and (ii) are direct consequences of (6.2) and the definitions of *J* and ${{\,\textrm{Jac}\,}}.$

For items (iii) and (iv), we use the fact that the determinant is the sum of products of coefficients of the matrix. More precisely, the Leibniz formula states that

6.9 $$\begin{aligned} \det (M) := \sum _{\sigma \in S_n} {{\,\textrm{sgn}\,}}(\sigma ) \prod _{i=1}^n M_{i,\sigma (i)}, \end{aligned}$$

where $S_n$ is the sets of permutations of $\{1,\dots ,n\}$ and ${{\,\textrm{sgn}\,}}$ is the signature. So the difference between the determinant of two matrices $M_1$ and $M_2$ is the sum of products of coefficients of $M_1$ and $M_2-M_1,$ and each product contains at least one coefficient of $M_2-M_1.$ With this observation, (iii) and (iv) follow from (i) and (ii).

The items (iii) and (iv) shows that both $\det (J)$ and $\det ({{\,\textrm{Jac}\,}})$ are close to 1—say in (1/2, 2)—as long as $\epsilon _0$ is small enough. This implies that

6.10 $$\begin{aligned} \left| \frac{1}{\det ({{\,\textrm{Jac}\,}})} - \frac{1}{\det (J)}\right| = \left| \frac{\det (J) - \det ({{\,\textrm{Jac}\,}})}{\det ({{\,\textrm{Jac}\,}})\det (J)} \right| \lesssim |\partial _t b| + |t\nabla _{p,t} \nabla _p b^t| \end{aligned}$$

by (iv). Cramer’s rule states that the coefficients of $M^{-1}$ is the quotient of a linear combination of product of coefficients of *M* over $\det (M).$ By using Cramer’s rule to ${{\,\textrm{Jac}\,}}$ and *J*,  (6.10), and (ii), we obtain (v).

Finally, the bound on $\nabla \det (J)$ and $\nabla J^{-1}$ are obtained by taking the gradient respectively in (6.9) and in Cramer’s rule. $\square$

#### Lemma 41

For any $p\in P$ and $t\in P^\bot {\setminus }\{0\},$ we have

6.12 $$\begin{aligned} (1-C\epsilon _0) |t| \le {{\,\textrm{dist}\,}}(\rho _{\mathcal {S}}(p,t),\Gamma _{\mathcal {S}}) \le |\rho _{\mathcal {S}}(p,t) - {\mathfrak {b}}(p)| \le (1+C\epsilon _0) |t| \end{aligned}$$

and

6.13 $$\begin{aligned} |\rho _{\mathcal {S}}(p,t) - {\mathfrak {b}}(p) - (0,t)| \le C\epsilon _0 |t|, \end{aligned}$$

where $C>0$ depends only on *n* (and $\eta )$.

#### Proof

The lemma is an analogue of Lemma 3.40 in [23]. But since the lemma is key to understand why $\rho _{\mathcal {S}}$ is a bi-Lipschitz change of variable, and since it is much easier in our case, we prove it carefully.

By definition of $\rho _{\mathcal {S}},$

$$\begin{aligned} \rho _{\mathcal {S}}(p,t) - {\mathfrak {b}}(p) - (0,t) = (-t(\nabla b^t(p))^T, b^t(p) - b(p)). \end{aligned}$$

So the mean value theorem applied to the continuous function $t \mapsto b^t(p)$ [recall that *b* is Lipschitz and $b^t$ is the convolution of *b* with a mollifier, so we even have a uniform convergence of $b^t$ to *b*] entails that

6.14 $$\begin{aligned} \begin{aligned} |\rho _{\mathcal {S}}(p,t) - {\mathfrak {b}}(p) - (0,t)|&\le |t\nabla b^t(p)| + |b^t(p) - b(p)| \\&\le |t\nabla b^t(p)| + |t| \sup _{s\in (0,|t|)} |\partial _s b^s(p)| \lesssim \epsilon _0|t| \end{aligned} \end{aligned}$$

by (6.2). Therefore, (6.13) is proven and we have

6.15 $$\begin{aligned} |\rho _{\mathcal {S}}(p,t) - {\mathfrak {b}}(p)| \le (1+C\epsilon _0) |t|, \end{aligned}$$

is the upper bound in (6.12). The middle bound of (6.12) is immediate, since ${\mathfrak {b}}(p) \in \Gamma _{\mathcal {S}}.$ It remains thus to prove the lower bound in (6.12). Let $q\in P$ be such that $|\rho _{\mathcal {S}}(p,t) - {\mathfrak {b}}(q)| = {{\,\textrm{dist}\,}}(\rho _{\mathcal {S}}(p,t), \Gamma _{\mathcal {S}}).$ We know that

$$\begin{aligned} |{\mathfrak {b}}(q) - {\mathfrak {b}}(p)| \le |{\mathfrak {b}}(q) - \rho _{\mathcal {S}}(p,t)| + |\rho _{\mathcal {S}}(p,t) - {\mathfrak {b}}(p)| \le 2|\rho _{\mathcal {S}}(p,t) - {\mathfrak {b}}(p)| \le 3|t|, \end{aligned}$$

if $\epsilon _0\ll 1$ is small enough, hence $|q-p| \le 3|t|$ too. So

$$\begin{aligned}\begin{aligned} {{\,\textrm{dist}\,}}(\rho _{\mathcal {S}}(p,t), \Gamma _{\mathcal {S}})&= |\rho _{\mathcal {S}}(p,t) - {\mathfrak {b}}(q)| \\&\ge |{\mathfrak {b}}(p) - {\mathfrak {b}}(q) + (0,t)| - |\rho _{\mathcal {S}}(p,t) - {\mathfrak {b}}(p) - (0,t)| \\&\ge |b(p) - b(q) + t| - C\epsilon _0|t| \end{aligned}\end{aligned}$$

by (6.13). But by Lemma 3.40, the function *b* is $2\epsilon _0$-Lipschitz, so we can continue with

$$\begin{aligned} {{\,\textrm{dist}\,}}(\rho _{\mathcal {S}}(p,t), \Gamma _{\mathcal {S}}) \ge (1-C{\epsilon _0})|t| - |b(p)-b(q)| \\ \ge (1-C{\epsilon _0}) |t| - 2\epsilon _0 |p-q| \ge (1 - C'{\epsilon _0}) |t|. \end{aligned}$$

The lemma follows. $\square$

#### Lemma 42

The map $\rho _{\mathcal {S}}$ is a bi-Lipschitz change of variable that maps *P* to $\Gamma _{\mathcal {S}}.$

#### Proof

See Theorem 3.53 in [23] for more details. We shall show that $\rho _{\mathcal {S}}$ is a bi-Lipschitz change of variable from $P \times (0,\infty )$ to

$$\begin{aligned}\Omega ^+_{\mathcal {S}} := \{(p,t) \in P \times P^\bot , \, t>b(p)\}\end{aligned}$$

and a similar argument also give that $\rho _{\mathcal {S}}$ is a bi-Lipschitz change of variable from $P \times (-\infty ,0)$ to

$$\begin{aligned}\Omega ^-_{\mathcal {S}} := \{(p,t) \in P \times P^\bot , \, t<b(p)\}.\end{aligned}$$

The lemma follows because we know that $\rho _{\mathcal {S}}$ is continuous on $P \times P^\bot .$

First, we know by the lower bound in (6.12) that the range of $\rho _{\mathcal {S}}(P\times (0,\infty ))$ never intersects $\Gamma _{\mathcal {S}},$ so since $\rho _{\mathcal {S}}$ is connected, it means that $\rho _{\mathcal {S}}(P\times (0,\infty ))$ is included in either $\Omega ^+_{\mathcal {S}}$ or $\Omega ^-_{\mathcal {S}}.$ A quick analysis of $\rho _{\mathcal {S}},$ for instance (6.13), shows that $\rho _{\mathcal {S}}(P\times (0,\infty )) \subset \Omega ^+_{\mathcal {S}}.$

At any point $(p,t) \in P\times (0,\infty )$ the Jacobian of $\rho _{\mathcal {S}}$ is close to the identity, as shown by (i) and (ii) of Lemma 6.8. So $\rho _{\mathcal {S}}$ is a local diffeomorphism, and the inversion function theorem shows that there exists a neighborhood $V_{p,t} \subset P\times (0,\infty )$ of (*p*, *t*) such that $\rho _{\mathcal {S}}$ is a bijection between $V_{p,t}$ and its range $\rho (V_{p,t}),$ which is a neighborhood of $\rho _{\mathcal {S}}(p,t).$ Since the Jacobian is uniformly close to the identity, all the $\rho _{\mathcal {S}}:\, V_{p,t} \mapsto \rho (V_{p,t})$ are bi-Lipschitz maps with uniform Lipschitz constant.

If $z\in \Omega ^+_{\mathcal {S}},$ we define the degree of the map $\rho _{\mathcal {S}}$ as

$$\begin{aligned} N(z) := ``\text {number of points }(p,t) \in P\times (0,\infty )\text { s.t. } \rho (p,t) = z'' \in \mathbb {N}\cup \{+\infty \}. \end{aligned}$$

We want to prove that *N*(*z*) is constantly equal to 1. If this is true, then the lemma is proven and we can construct the inverse $\rho ^{-1}$ locally by inversing the appropriate bijection $\rho _{\mathcal {S}}:\, V_{p,t} \mapsto \rho (V_{p,t}).$

We already know that the number of points that satisfy $\rho (p,t) = z$ is countable, because we can cover $P\times (0,\infty )$ by a countable union of the neighborhoods $V_{p,t}$ introduced before. Moreover, if $N(z) \ge v >0,$ then we can find *v* points $(p_i,t_i)\in P\times (0,\infty )$ such that $\rho _{\mathcal {S}}(p_i,t_i) = z$ and so *v* disjoint neighborhoods $V_{p_i,t_i}$ of $(p_i,t_i).$ Consequently, each point $z'$ in the neighborhood $\bigcap _i \rho _{\mathcal {S}}(V_{p_i,t_i})$ of *z* satisfies $N(z') \ge v.$ This proves that *N* is constant on any connected component, that is

$$\begin{aligned} N\text { is constant on }\Omega ^+_{\mathcal {S}}. \end{aligned}$$

It remains to prove that $N(z_0) = 1$ for one point $z_0$ in $\Omega ^+_{\mathcal {S}}.$ Take $p_0$ far from the support of *b*,  for instance ${{\,\textrm{dist}\,}}(p_0,\Pi (Q(\mathcal {S})) \ge 99 \ell (Q(\mathcal {S}))$ and $t_0 = \ell (Q(\mathcal {S})).$ In this case, we have $\rho _{\mathcal {S}}(p_0,t_0) = (p_0,t_0)$ and ${{\,\textrm{dist}\,}}(\rho _{\mathcal {S}}(p_0,t_0),\Gamma _{\mathcal {S}}) = t_0.$ Let $(p_1,t_1) \in P \times (0,\infty )$ be such that $\rho _{\mathcal {S}}(p_1,t_1) = (p_0,t_0),$ the bound (6.12) entails that $|t_1-t_0| \le C\epsilon _1 |t_0| \le \ell (Q(\mathcal {S}))$ and (6.12) implies that $|p_1 - p_0| \le C\epsilon _0 |t_1| \le \ell (Q(\mathcal {S})).$ Those conditions force $p_1$ to stay far away from the support of *b*,  which implies that $\rho _{\mathcal {S}}(p_1,t_1) = (p_1,t_1) = (p_0,t_0).$ We just proved that $N(p_0,t_0) = 1,$ as desired. $\square$

### Properties of the operator $L_{\mathcal {S}}$

#### Lemma 43

Let $L = - \mathop {{\text {div}}}\mathcal {A}\nabla$ be a uniformly elliptic operator satisfying (1.6) and (1.7) on $\Omega .$ Construct on $\rho _{\mathcal {S}}^{-1}(\Omega )$ the operator $L_{\mathcal {S}} =-\mathop {{\text {div}}}\mathcal {A}_{\mathcal {S}} \nabla$ with

6.18 $$\begin{aligned} \mathcal {A}_{\mathcal {S}}(p,t) := \det ({{\,\textrm{Jac}\,}}(p,t)) {{\,\textrm{Jac}\,}}^{-T}(p,t) \mathcal {A}(\rho _{\mathcal {S}}(p,t)) {{\,\textrm{Jac}\,}}^{-1}(p,t) \end{aligned}$$

for $(p,t) \in \rho _{\mathcal {S}}^{-1}(\Omega ).$ Then $L_{\mathcal {S}}$ is the conjugate operator of *L* by $\rho _{\mathcal {S}},$ that is,  $u\circ \rho _{\mathcal {S}}$ is a weak solution to $L_{\mathcal {S}}(u\circ \rho _{\mathcal {S}}) = 0$ in $\rho _{\mathcal {S}}^{-1}(\Omega )$ if and only if *u* is a weak solution to $Lu = 0$ in $\Omega .$

#### Proof

The maps $\rho _{\mathcal {S}}$ is a bi-Lipschitz change of variable on $\mathbb {R}^n = P \times P^\bot ,$ so the construction (6.18) properly define a matrix of coefficients in $L^\infty (\rho _{\mathcal {S}}^{-1}(\Omega )).$

Let *u* be a weak solution to $Lu=0$ in $\Omega .$ Then, for any $\varphi \in C^\infty _0(\rho _{\mathcal {S}}^{-1}(\Omega )),$ we have

$$\begin{aligned}{} & {} \iint _{\mathbb {R}^n} \mathcal {A}_{\mathcal {S}} \nabla (u\circ \rho _{\mathcal {S}}) \cdot \nabla \varphi \, dp\, dt \\{} & {} \quad = \iint _{\mathbb {R}^n} \det ({{\,\textrm{Jac}\,}}) {{\,\textrm{Jac}\,}}^{-T} (\mathcal {A}\circ \rho _{\mathcal {S}}) {{\,\textrm{Jac}\,}}^{-1} \nabla (u\circ \rho _{\mathcal {S}}) \cdot \nabla \varphi \, dp\, dt \\{} & {} \quad = \iint _{\mathbb {R}^n} \det ({{\,\textrm{Jac}\,}}) (\mathcal {A}\circ \rho _{\mathcal {S}}) {{\,\textrm{Jac}\,}}^{-1} \nabla (u\circ \rho _{\mathcal {S}}) \cdot {{\,\textrm{Jac}\,}}^{-1} \nabla \varphi \, dp\, dt \\{} & {} \quad = \iint _{\mathbb {R}^n} \det ({{\,\textrm{Jac}\,}}) (\mathcal {A}\circ \rho _{\mathcal {S}}) (\nabla u \circ \rho _{\mathcal {S}}) \cdot (\nabla [\varphi \circ \rho _{\mathcal {S}}^{-1}] \circ \rho _{\mathcal {S}})\, dp\, dt \end{aligned}$$

because $\nabla (f\circ \rho _{\mathcal {S}})$ is equal to the matrix multiplication ${{\,\textrm{Jac}\,}}(\nabla f \circ \rho _{\mathcal {S}})$ by definition of the Jacobian. Recall that $\det ({{\,\textrm{Jac}\,}}) >0,$ so doing the change of variable $X=\rho _{\mathcal {S}}(p,t)$ gives

6.19 $$\begin{aligned} \iint _{\mathbb {R}^n} \mathcal {A}_{\mathcal {S}} \nabla (u\circ \rho _{\mathcal {S}}) \cdot \nabla \varphi \, dp\, dt = \iint _{\mathbb {R}^n} \mathcal {A}\nabla u \cdot \nabla [\varphi \circ \rho _{\mathcal {S}}^{-1}] \, dX. \end{aligned}$$

The function $\varphi \circ \rho _{\mathcal {S}}^{-1}$ may not be smooth anymore, but is still compactly supported in $\Omega$ and in $W^{1,\infty }(\Omega ) \subset W^{1,2}_{loc}(\Omega ),$ so $\varphi \circ \rho _{\mathcal {S}}^{-1}$ is a valid test function for the weak solution *u*,  and so the right-hand side of (6.19) is 0. We conclude that

$$\begin{aligned} \iint _{\mathbb {R}^n} \mathcal {A}_{\mathcal {S}} \nabla (u\circ \rho _{\mathcal {S}}) \cdot \nabla \varphi \, dp\, dt = 0 \end{aligned}$$

for any $\varphi \in C^\infty _0(\rho _{\mathcal {S}}^{-1}(\Omega )),$ hence $u\circ \rho _{\mathcal {S}}$ is a weak solution to $L_{\mathcal {S}}(u\circ \rho _{\mathcal {S}}) = 0$ in $\rho _{\mathcal {S}}^{-1}(\Omega ).$

The same reasoning shows that *u* is a weak solution to $Lu=0$ in $\Omega$ whenever $u\circ \rho _{\mathcal {S}}$ is a weak solution to $L_{\mathcal {S}}(u\circ \rho _{\mathcal {S}}) = 0$ in $\rho _{\mathcal {S}}^{-1}(\Omega ).$ The lemma follows. $\square$

We want to say that $A_{\mathcal {S}}$ satisfies the same Carleson-type condition as $A \circ \rho _{\mathcal {S}}.$ For instance, we want to say that $\delta \nabla A \in CM_{\Omega }$—which implies $(\delta \circ \rho _{\mathcal {S}}) \nabla (A\circ \rho _{\mathcal {S}}) \in CM_{\rho _{\mathcal {S}}^{-1}(\Omega )}$—will give that $(\delta \circ \rho _{\mathcal {S}}) \nabla A_{\mathcal {S}} \in CM_{\rho _{\mathcal {S}}^{-1}(\Omega )}.$ However, it is not true, for the simple reason that the Carleson estimates on ${{\,\textrm{Jac}\,}}$ are related to the set $\mathbb {R}^n {\setminus } P$ while the ones on $A \circ \rho _{\mathcal {S}}$ are linked to the domain $\rho _{\mathcal {S}}^{-1}(\Omega ).$ Since $A_{\mathcal {S}}$ is the product of these two objects, we only have Carleson estimates for $A_{\mathcal {S}}$ in the areas of $\mathbb {R}^n$ where $\rho _{\mathcal {S}}(\partial \Omega )$ looks like *P*.

#### Lemma 44

Assume that the matrix function $\mathcal {A}$ defined on $\Omega$ satisfies (1.6) and (1.7),  and can be decomposed as $\mathcal {A}=\mathcal {B}+\mathcal {C}$ where

6.21 $$\begin{aligned} |\delta \nabla \mathcal {B}| + |\mathcal {C}| \in CM_{\Omega }(M). \end{aligned}$$

Then the matrix $\mathcal {A}_{\mathcal {S}}$ constructed in (6.18) can also be decomposed as $\mathcal {A}_{\mathcal {S}} = \mathcal {B}_{\mathcal {S}} + \mathcal {C}_{\mathcal {S}}$ where $\mathcal {B}_{\mathcal {S}}$ satisfies (1.6) and (1.7) with the constant $2C_{\mathcal {A}},$ $|t\nabla \mathcal {B}_{\mathcal {S}}|$ is uniformly bounded by $CC_{\mathcal {A}},$ and

6.22 $$\begin{aligned} (|t \nabla \mathcal {B}_{\mathcal {S}}| + |\mathcal {C}_{\mathcal {S}}|){\mathbb {1}}_{\rho ^{-1}_{\mathcal {S}}(W^*_{\Omega }(\mathcal {S}))} \in CM_{\mathbb {R}^n {\setminus }P}(C(\epsilon _0^2+M)) \end{aligned}$$

for a constant *C* that depends only on *n* and the ellipticity constant $C_\mathcal {A}.$

#### Proof

Let $\mathcal {A}= \mathcal {B}+ \mathcal {C}$ as in the lemma. Without loss of generality, we can choose $\mathcal {B}$ to be a smooth average of $\mathcal {A}$ (see Lemma 2.1) and so $\mathcal {B}$ satisfies (1.6) and (1.7) with the constant $C_{\mathcal {A}}$ and $|\delta \nabla \mathcal {B}| \le CC_\mathcal {A}.$ Define

$$\begin{aligned}\mathcal {B}_{\mathcal {S}} := \det (J) J^{-T} (\mathcal {B}\circ \rho _{\mathcal {S}}) J^{-1} \end{aligned}$$

and of course $\mathcal {C}_{\mathcal {S}} := \mathcal {A}_{\mathcal {S}} - \mathcal {B}_{\mathcal {S}}.$ First, Lemma 6.8 shows that $\det (J)$ is close to 1 and $J^{-1}$ is close to the identity, so $\mathcal {B}_{\mathcal {S}}$ satisfies (1.6) and (1.7) with the constant $(1+C\epsilon _0)C_\mathcal {A}\le 2C_\mathcal {A}.$ Moreover, the same Lemma 6.8 gives that $|\det (J)| + \Vert J^{-1}\Vert \le 3,$ $\Vert {{\,\textrm{Jac}\,}}- I\Vert \le 3,$ and $\displaystyle |\nabla _{p,t} \det (J)| + \Vert |\nabla _{p,t} J^{-1}|\Vert \lesssim |\nabla _{p,t} \nabla _p b^t|,$ and hence

$$\begin{aligned}|\nabla \mathcal {B}_{\mathcal {S}}| \lesssim |(\nabla \mathcal {B}) \circ \rho _{\mathcal {S}}| + |\nabla _{p,t} \nabla _p b^t|,\end{aligned}$$

and

$$\begin{aligned}\begin{aligned} |\mathcal {C}_{\mathcal {S}}|&\lesssim |\det ({{\,\textrm{Jac}\,}}) - \det (J)| + \Vert {{\,\textrm{Jac}\,}}^{-1} - J^{-1}\Vert + |\mathcal {C}\circ \rho _{\mathcal {S}}| \\&\lesssim |\partial _t b^t| + |t\nabla _{p,t} \nabla _p b^t| + |\mathcal {C}\circ \rho _{\mathcal {S}}|. \end{aligned}\end{aligned}$$

Lemma 6.1 entails that $|t\nabla _{p,t} \nabla _p b^t| \lesssim \epsilon _0 \le 1 \le C_{\mathcal {A}},$ so $\mathcal {B}_{\mathcal {S}}$ verifies $|t\nabla \mathcal {B}_{\mathcal {S}}| \lesssim C_\mathcal {A},$ so thus (6.22) is the only statement we still have to prove. Lemma 6.1 also implies that $|\partial _t b^t| + |t\nabla _{p,t} \nabla _p b^t| \in CM_{P\times (0,\infty )}(C\epsilon _0^2).$ Therefore, it suffices to establish that

6.23 $$\begin{aligned} (|t\nabla \mathcal {B}\circ \rho _{\mathcal {S}}| + |\mathcal {C}\circ \rho _{\mathcal {S}}|) {\mathbb {1}}_{\rho ^{-1}_{\mathcal {S}}(W^*_{\Omega }(\mathcal {S}))} \in CM_{P\times (0,\infty )}(CM). \end{aligned}$$

Take $p_0 \in P$ and $r_0>0.$ We want to show that

6.24 $$\begin{aligned} \iint _{B(p_0,r_0) \cap \rho ^{-1}_{\mathcal {S}}(W^*_{\Omega }(\mathcal {S}))} (|t\nabla \mathcal {B}\circ \rho _{\mathcal {S}}|^2 + |\mathcal {C}\circ \rho _{\mathcal {S}}|^2) \, \frac{dt}{t} \, dp \le CM(r_0)^{n-1}. \end{aligned}$$

If $\rho _{\mathcal {S}}(B(p_0,r_0)) \cap W^*_{\Omega }(\mathcal {S}) = \emptyset ,$ the left-hand side above is zero and there is nothing to prove. Otherwise, pick a point $X\in \rho _{\mathcal {S}}(B(p_0,r_0)) \cap W^*_{\Omega }(\mathcal {S}).$ The fact that $X\in \rho _{\mathcal {S}}(B(p_0,r_0))$ means that

6.25 $$\begin{aligned} |X-{\mathfrak {b}}(p_0)| \le (1+C\epsilon _0)r_0, \end{aligned}$$

since $\rho _{\mathcal {S}}(p_0) = {\mathfrak {b}}(p_0)$ and $\Vert {{\,\textrm{Jac}\,}}- I\Vert \le C\epsilon _0$ by Lemma 6.8. Because *b* is $2\epsilon _0$-Lipschitz with $\epsilon _0 \ll 1,$ we deduce

6.26 $$\begin{aligned} |X-{\mathfrak {b}}(\Pi (X))| \le (1+\epsilon _0) |X-{\mathfrak {b}}(p_0)| \le (1+2C\epsilon _0)r_0. \end{aligned}$$

The fact that $X\in W^*_{\Omega }(\mathcal {S})$ implies by (4.14) that

6.27 $$\begin{aligned} \delta (X) \le (1+2\epsilon _0)|X-{\mathfrak {b}}(\Pi (X))| \le 2r_0 \end{aligned}$$

thanks to (6.26). Moreover, if $x \in \partial \Omega$ is such that $|X-x| = \delta (X)$,

6.28 $$\begin{aligned} |x-{\mathfrak {b}}(p_0)|\le & {} |x-{\mathfrak {b}}(\Pi (X))| + |{\mathfrak {b}}(\Pi (X) - {\mathfrak {b}}(p_0)| \nonumber \\\le & {} 2\epsilon _0\delta (X) + (1+\epsilon _0) |\Pi (X) - p_0| \le \frac{1}{2} r_0 + (1+\epsilon _0) |X-{\mathfrak {b}}(p_0)| \le 2r_0 \nonumber \\ \end{aligned}$$

by using in order (4.16), the fact that *b* is $2\epsilon _0$-Lipschitz, (6.27), and (6.25). Fix $X_0 \in \rho _{\mathcal {S}}(B(p_0,r_0)) \cap W^*_{\Omega }(\mathcal {S})$ and $x_0\in \partial \Omega$ such that $|X_0 - x_0| = \delta (X_0).$ The inequalities (6.25) and (6.28) show that,

$$\begin{aligned} |X-x_0| \le |X-{\mathfrak {b}}(p_0)| + |x_0 - {\mathfrak {b}}(p_0)| \le 4r_0 \quad \text {for } X\in \rho _{\mathcal {S}}(B(p_0,r_0)) \cap W^*_{\Omega }(\mathcal {S}), \end{aligned}$$

that is

6.29 $$\begin{aligned} \rho _{\mathcal {S}}(B(p_0,r_0)) \cap W^*_{\Omega }(\mathcal {S}) \subset B(x_0,4r_0). \end{aligned}$$

We are now ready to conclude. We make the change of variable $X = \rho _{\mathcal {S}}(p,s)$ in (6.24), and since $\rho _{\mathcal {S}}$ is a bi-Lipschitz change of variable that almost preserves the distances (because $\Vert {{\,\textrm{Jac}\,}}- I\Vert \le C\epsilon _0 \ll 1)$, we obtain

$$\begin{aligned}{} & {} \iint _{B(p_0,r_0) \cap \rho ^{-1}_{\mathcal {S}}(W^*_{\Omega }(\mathcal {S}))} (|t\nabla \mathcal {B}\circ \rho _{\mathcal {S}}|^2 + |\mathcal {C}\circ \rho _{\mathcal {S}}|^2) \, \frac{dt}{t} \, dp \\{} & {} \quad \le 2 \iint _{B(x_0,4r_0) \cap W^*_{\Omega }(\mathcal {S})} (|{{\,\textrm{dist}\,}}(X,\Gamma _{\mathcal {S}}) \nabla \mathcal {B}|^2 + |\mathcal {C}|^2) \, \frac{dX}{{{\,\textrm{dist}\,}}(X,\Gamma _{\mathcal {S}})} \\{} & {} \quad \le 4 \iint _{B(x_0,4r_0)} (|\delta \nabla \mathcal {B}|^2 + |\mathcal {C}|^2) \, \frac{dX}{\delta (X)} \le CM(r_0)^{n-1} \end{aligned}$$

by using (4.15) and then the fact that $|\delta \nabla \mathcal {B}| + |\mathcal {C}| \in CM_{\Omega }(M).$ The lemma follows. $\square$

### Properties of the composition of the smooth distance by $\rho _{\mathcal {S}}$

The change of variable $\rho _{\mathcal {S}}$ maps $P\times (P^\bot {\setminus }\{0\})$ to $\mathbb {R}^n {\setminus } \Gamma _{\mathcal {S}},$ so for any $X\in \mathbb {R}^n {\setminus }\Gamma _{\mathcal {S}},$ the quantities $N_{\rho ^{-1}_{\mathcal {S}}(X)}(Y)$ and $\Lambda (\rho ^{-1}_{\mathcal {S}}(X))$ make sense as $N_{p,t}(Y)$ and $\Lambda (p,t),$ respectively, where $(p,t) = \rho ^{-1}_{\mathcal {S}}(X).$ With this in mind, we have the following result.

#### Lemma 45

For any $Q\in \mathcal {S},$ we have

6.31 $$\begin{aligned} \iint _{W_{\Omega }(Q)} \left| \frac{\nabla D_{\beta }(X)}{D_{\beta }(X)} - \frac{N_{\rho ^{-1}_{\mathcal {S}}(X)}(X)}{{{\,\textrm{dist}\,}}(X,\Lambda (\rho ^{-1}_{\mathcal {S}}(X))} \right| ^2\, \delta (X) \, dX \le C |\alpha _{\sigma ,\beta }(Q)|^2 \sigma (Q), \nonumber \\ \end{aligned}$$

with a constant $C>0$ that depends only on *n*,  $C_{\sigma },$ and $\beta .$

#### Proof

The lemma is a consequence of Corollary 5.52 and the definition of $\rho _{\mathcal {S}}.$

First, Lemma 4.1(d) entails that $W_{\Omega }(Q) \subset \mathbb {R}^n {\setminus } \Gamma _{\mathcal {S}},$ which means that the quantities $N_{\rho ^{-1}_{\mathcal {S}}(X)}$ and $\Lambda (\rho ^{-1}_{\mathcal {S}}(X))$ are well defined in (6.31). Let $X\in W_{\Omega }(Q)$ and set $(p,t) = \rho ^{-1}_{\mathcal {S}}(X).$

On one hand, Lemma 6.11 gives that

$$\begin{aligned} {{\,\textrm{dist}\,}}(X,\Gamma _{\mathcal {S}}) \le |X- {\mathfrak {b}}(p)| \le (1+C\epsilon _0) |t| \le (1+C'\epsilon _0) {{\,\textrm{dist}\,}}(X,\Gamma _{\mathcal {S}}) \end{aligned}$$

and

$$\begin{aligned}|X - {\mathfrak {b}}(p) - (0,t)| \le C\epsilon _0|t|.\end{aligned}$$

By projecting the left-hand side on *P*,  the latter implies that

$$\begin{aligned}|\Pi (X) - p| \le C\epsilon _0|t|.\end{aligned}$$

On the other hand, since $X\in W_{\Omega }(Q),$ Lemma 4.13 gives that

$$\begin{aligned}{{\,\textrm{dist}\,}}(X,\Gamma _{\mathcal {S}}) \le |X- {\mathfrak {b}}(\Pi (X))| \le (1+2\epsilon _0) \delta (X) \le (1+C\epsilon _0) {{\,\textrm{dist}\,}}(X,\Gamma _{\mathcal {S}}),\end{aligned}$$

and, if $x \in Q$ is such that $|X-x| = \delta (X),$ then by (4.16),

$$\begin{aligned} |{\mathfrak {b}}(\Pi (X)) - x| \le 2 \epsilon _0 \delta (X), \end{aligned}$$

which implies that

$$\begin{aligned} |\Pi (X) - \Pi (x)| \le 2 \epsilon _0 \delta (X), \end{aligned}$$

Altogether, we have

$$\begin{aligned} \delta (X) (1-C\epsilon _0) \le |t| \le (1+C\epsilon _0) \delta (X) \end{aligned}$$

and

$$\begin{aligned} {{\,\textrm{dist}\,}}(p,\Pi (Q)) \le |p - \Pi (x)|\le \left| p-\Pi (X)\right| +\left| \Pi (X)-\Pi (x)\right| \le C\epsilon _0 \delta (X). \end{aligned}$$

If we throw in the fact that $\delta (X) \in [\ell (Q)/2,\ell (Q)]$ by definition of $W_{\Omega }(Q),$ then we easily observe that *p* and *t* satisfy the assumptions of Corollary 5.52, and so

$$\begin{aligned} \left| \frac{\nabla D_{\beta }(X)}{D_{\beta }(X)} - \frac{N_{\rho ^{-1}_{\mathcal {S}}(X)}(X)}{{{\,\textrm{dist}\,}}(X,\Lambda (\rho ^{-1}_{\mathcal {S}}(X))} \right| \le C \ell (Q)^{-1} \alpha _{\sigma ,\beta }(Q) \quad \text {for } X\in W_{\Omega }. \end{aligned}$$

We conclude that

$$\begin{aligned}{} & {} \iint _{W_{\Omega }(Q)} \left| \frac{\nabla D_{\beta }(X)}{D_{\beta }(X)} - \frac{N_{\rho ^{-1}_{\mathcal {S}}(X)}(X)}{{{\,\textrm{dist}\,}}(X,\Lambda (\rho ^{-1}_{\mathcal {S}}(X))} \right| ^2\, \delta (X) \, dX \\{} & {} \quad \le C |W_{\Omega }(Q)| |\ell (Q)^{-1} \alpha _{\sigma ,\beta }(Q)|^2 \ell (Q) \le C |\alpha _{\sigma ,\beta }(Q)|^2 \sigma (Q) \end{aligned}$$

because $|W_{\Omega }(Q)| \approx \sigma (Q) \ell (Q)$ by (4.8) and (1.1). The lemma follows. $\square$

#### Lemma 46

We have

6.33 $$\begin{aligned} \iint _{\rho _{\mathcal {S}}^{-1}(W_{\Omega }(\mathcal {S}))} \left| \frac{\nabla t}{t} - \frac{{{\,\textrm{Jac}\,}}(p,t) N_{p,t}(\rho _{\mathcal {S}}(p,t))}{{{\,\textrm{dist}\,}}(\rho _{\mathcal {S}}(p,t), \Lambda (p,t))}\right| ^2 \left| t\right| \, dt\, dp \le C(\epsilon _0)^2 \sigma (Q(\mathcal {S})) \nonumber \\ \end{aligned}$$

where $C>0$ depends only on *n* (and $\eta )$.

#### Proof

From the definition, we can see that $\Lambda (p,t)$ is the affine plane that goes through the point ${\mathfrak {b}}^t(p)$ and whose directions are given by the vectors $(q,q \nabla b^t(p)),$ that is $\Lambda (p,t)$ is the codimension 1 plane that goes through ${\mathfrak {b}}^t(p)$ and with upward unit normal vector

$$\begin{aligned} N_{p,t} = \frac{1}{|(-(\nabla b^r(p))^T,1)|} \begin{pmatrix} -\nabla b^t(p) \\ 1 \end{pmatrix} = \frac{1}{\sqrt{1+|\nabla b^t(p)|^2}} \begin{pmatrix} -\nabla b^t(p) \\ 1 \end{pmatrix}. \end{aligned}$$

The vector function $N_{p,t}(X)$ is just $+N_{p,t}$ or $-N_{p,t},$ depending whether *X* lies above or below $\Lambda (p,t).$

Observe that $\rho _{\mathcal {S}}(p,t) - {\mathfrak {b}}^t(p) = t(-(\nabla b^t(p))^T,1),$ which means that ${\mathfrak {b}}^t(p)$ is the projection of $\rho _{\mathcal {S}}(p,t)$ onto $\Lambda (p,t)$ and that

$$\begin{aligned} {{\,\textrm{dist}\,}}(\rho _{\mathcal {S}}(p,t), \Lambda (p,t)) = |t| |(-(\nabla b^t(p))^T,1)| = |t| \sqrt{1+|\nabla b^t(p)|^2}. \end{aligned}$$

Moreover, $\rho _{\mathcal {S}}(p,t)$ lies above $\Lambda (p,t)$ if $t>0$ and below otherwise, that is

$$\begin{aligned} N_{p,t}(\rho (p,t)) = {{\,\textrm{sgn}\,}}(t) N_{p,t} = \frac{{{\,\textrm{sgn}\,}}(t)}{\sqrt{1+|\nabla b^t(p)|^2}} \begin{pmatrix} -\nabla b^t(p) \\ 1 \end{pmatrix}. \end{aligned}$$

From all this, we deduce

6.34 $$\begin{aligned} \frac{J(p,t) N_{p,t}(\rho _{\mathcal {S}}(p,t))}{{{\,\textrm{dist}\,}}(\rho _{\mathcal {S}}(p,t), \Lambda (p,t))}= & {} \frac{1}{t(1+|\nabla b^t(p)|^2)} \begin{pmatrix} I &{} \nabla b^t(p) \\ -(\nabla b^t(p))^T &{} 1 \end{pmatrix} \begin{pmatrix} -\nabla b^t(p) \\ 1 \end{pmatrix}\nonumber \\= & {} \frac{1}{t} \begin{pmatrix} 0_{\mathbb {R}^{n-1}} \\ 1 \end{pmatrix} = \frac{\nabla t}{t}. \end{aligned}$$

Recall that $|{{\,\textrm{Jac}\,}}- J| \lesssim |\partial _t b^t| + |t \nabla _{p,t} \nabla _p b^t|.$ Together with (6.34), we obtain that the left-hand side of (6.33) is equal to

6.35 $$\begin{aligned}{} & {} I = \iint _{\rho ^{-1}_{\mathcal {S}}(W_{\Omega }(\mathcal {S}))} \left| \frac{{{\,\textrm{Jac}\,}}(p,t) - J(p,t)}{t(1+|\nabla b^t(p)|^2)} \begin{pmatrix} -\nabla b^t(p) \\ 1 \end{pmatrix} \right| ^2 \left| t\right| \, dt\, dp \nonumber \\{} & {} \quad \lesssim \iint _{\rho _{\mathcal {S}}^{-1}(W_{\Omega }(\mathcal {S}))} (|\partial _t b^t|^2 + |t \nabla _{p,t} \nabla _p b^t|^2) \, \frac{dt}{\left| t\right| } \, dp. \end{aligned}$$

Take $X_0 \in W_{\Omega }(\mathcal {S}),$ and notice that the set $W_{\Omega }(\mathcal {S})$ is included in the ball $B({\mathfrak {b}}(\Pi (X_0)),4\ell (Q(\mathcal {S})))$ by definition of $W_{\Omega }(\mathcal {S})$ and by (4.16). Since the Jacobian of $\rho _{\mathcal {S}}$ is close to the identity, $\rho _{\mathcal {S}}^{-1}$ almost preserves the distance, and hence $\rho ^{-1}_{\mathcal {S}}(W_{\Omega }(\mathcal {S})) \subset B(\Pi (X_0),5\ell (Q(\mathcal {S}))).$ We conclude that

$$\begin{aligned} I\lesssim & {} \iint _{B(\Pi (X_0),5\ell (Q(\mathcal {S})))} (|\partial _t b^t|^2 + |t \nabla _{p,t} \nabla _p b^t|^2) \, \frac{dt}{t} \, dp \lesssim (\epsilon _0)^2 \ell (Q(\mathcal {S}))^{n-1} \\\lesssim & {} (\epsilon _0)^2 \sigma (Q(\mathcal {S})) \end{aligned}$$

by Lemma 6.1 and then (1.1). The lemma follows. $\square$

## The flat case

In this section, we intend to prove an analogue of Theorem 1.12 when the boundary is flat, that is when the domain is $\Omega _0:= \mathbb {R}^n_+.$ This is our main argument on the PDE side (contrary to other sections which are devoted to geometric arguments) and the general case of Chord-Arc Domains is eventually brought back to this simpler case.

We shall bring a little bit of flexibility in the following manner. We will allow $\Omega$ to be different from $\mathbb {R}^n_+,$ but we shall stay away from the parts where $\partial \Omega$ differs from $\partial \mathbb R^n_+$ with some cut-off functions. More exactly, we shall use cut-off functions $\phi$ that guarantee that $\delta (X) := {{\,\textrm{dist}\,}}(X,\partial \Omega ) \approx t$ whenever $X = (x,t) \in {{\,\textrm{supp}\,}}\phi .$ We shall simply use $\mathbb {R}^{n-1}$ for $\partial \mathbb R^n=\mathbb {R}^{n-1}\times \left\{ 0\right\} .$ We start with the precise definition of the cut-off functions that we are allowing.

### Definition 47

We say that $\phi \in L^\infty (\Omega )$ is a cut-off function associated to both $\partial \Omega$ and $\mathbb {R}^{n-1}$ if $0 \le \phi \le 1,$ and there is a constant $C_\phi \ge 1$ such that $|\nabla \phi | \le C_\phi \delta ^{-1},$

7.2 $$\begin{aligned} (C_\phi )^{-1} |t| \le \delta (X) \le C_\phi |t| \quad \text {for all } X=(x,t) \in {{\,\textrm{supp}\,}}\phi , \end{aligned}$$

and there exists a collection of dyadic cubes $\{Q_i\}_{i\in I_\phi }$ in ${\mathbb {D}}_{\partial \Omega }$ such that

7.3 $$\begin{aligned} \{Q_i\}_{i\in I_\phi }\text { is finitely overlapping with an overlap of at most }C_\phi , \end{aligned}$$

and

7.4 $$\begin{aligned} \Omega \cap ({{\,\textrm{supp}\,}}\phi ) \cap {{\,\textrm{supp}\,}}(1-\phi ) \subset \bigcup _{i\in I_\phi } W_{\Omega }^{**}(Q_i). \end{aligned}$$

The condition (7.2) allows us to say that

7.5 $$\begin{aligned}{} & {} \text { if, for }x\in \partial \Omega \text { and }r>0,~B(x,r) \cap {{\,\textrm{supp}\,}}\phi \ne \emptyset , \nonumber \\{} & {} \quad \text { then there exists }y\in \mathbb {R}^{n-1}\text { such that }B(x,r) \subset B(y,Cr); \end{aligned}$$

so we can pass from Carleson measures in $\Omega$ to Carleson measure in $\mathbb {R}^n {\setminus } \mathbb {R}^{n-1}.$ For instance, we have

7.6 $$\begin{aligned} \begin{array}{c} f \in CM_{\Omega }(M) \implies f\phi , \, f{\mathbb {1}}_{{{\,\textrm{supp}\,}}\phi } \in CM_{\mathbb {R}^n {\setminus } \mathbb {R}^{n-1}}(C'_\phi M), \\ \delta \nabla g \in CM_{\Omega }(M) \implies t \phi \nabla g \in CM_{\mathbb {R}^n {\setminus } \mathbb {R}^{n-1}}(C'_\phi M). \end{array} \end{aligned}$$

and vice versa. The conditions (7.3) and (7.4) ensure that ${\mathbb {1}}_{({{\,\textrm{supp}\,}}\phi ) \cap {{\,\textrm{supp}\,}}(1-\phi )}$ (and hence $\delta \nabla \phi )$ satisfies the Carleson measure condition on $\Omega .$ So by (7.6),

7.7 $$\begin{aligned} |t \nabla \phi | + {\mathbb {1}}_{{{\,\textrm{supp}\,}}\nabla \phi } + {\mathbb {1}}_{({{\,\textrm{supp}\,}}\phi ) \cap {{\,\textrm{supp}\,}}(1-\phi )} \in CM_{ \mathbb {R}^n {\setminus } \mathbb {R}^{n-1}}(C'_\phi ). \end{aligned}$$

And if the support of $\phi$ is contained in a ball of radius *r* centered on $\partial \Omega ,$ then

7.8 $$\begin{aligned} \iint _{\Omega } \big (|\nabla \phi |t + |t\nabla \phi |^2\big ) \, \frac{dt}{t}\, dy \lesssim r^{n-1}. \end{aligned}$$

We are ready to state the main result of the section.

### Lemma 48

Let $\Omega$ be a Chord-Arc Domain and let $L=-{{\,\textrm{div}\,}}{\mathcal {A}} \nabla$ be a uniformly elliptic operator on $\Omega ,$ that is $\mathcal {A}$ verifies (1.6) and (1.7). Assume that the $L^*$-elliptic measure $\omega _{L^*}\in A_{\infty }(\sigma ),$ where $L^*$ is the adjoint operator of *L*,  and $\sigma$ is an Ahlfors^1^ regular measure on $\partial \Omega .$ Let $\phi$ be as in Definition 7.1 and be supported in a ball $B:=B(x,r)$ centered on the boundary $\partial \Omega .$ Assume that the coefficients $\mathcal {A}$ can be decomposed as ${\mathcal {A}} = {\mathcal {B}} + {\mathcal {C}}$ where

7.10 $$\begin{aligned} (|t\nabla {\mathcal {B}}| + |{\mathcal {C}}|){\mathbb {1}}_{{{\,\textrm{supp}\,}}\phi } \in CM_{\mathbb {R}^n {\setminus }\mathbb {R}^{n-1}}(M). \end{aligned}$$

Then for any non-negative nontrivial weak solution *u* to $Lu = 0$ in $2B \cap \Omega$ with zero trace on $\partial \Omega \cap 2B,$ one has

7.11 $$\begin{aligned} \iint _{\Omega } |t|\left| \frac{\nabla u}{u} - \frac{\nabla t}{t} \right| ^2 \phi ^2 \, dt\, dy = \iint _{\Omega } |t|\left| \nabla \ln \Big ( \frac{u}{|t|} \Big ) \right| ^2 \phi ^2 \, dt\, dy \le C(1+M) r^{n-1}, \nonumber \\ \end{aligned}$$

where *C* depends only on the dimension *n*,  the elliptic constant $C_\mathcal {A},$ the 1-sided CAD constants of $\Omega ,$ the constant $C_\phi$ in Definition 7.1, and the intrinsic constants in $\omega _{L^*} \in A_{\infty }(\sigma ).$

The above lemma is the analogue of Theorem 2.21 from [25] in our context, and part of our proof will follow the one from [25] but a new argument is needed to treat the non-diagonal structure of ${\mathcal {A}}.$

We need $\omega _{L^*} \in A_{\infty }(\sigma )$ for the proof of the following intermediate lemma. Essentially, we need that the logarithm of the Poisson kernel lies in *BMO*. Let us state and prove it directly in the form that we need.

### Lemma 49

Let $\Omega ,$ *L*,  $\phi ,$ $B:=B(x,r),$ and *u* be as in Lemma 7.9. Assume that $\omega _{L^*}\in A_{\infty }(\sigma )$ as in Lemma 7.9. Then there exists $K:= K(u,B)$ such that

$$\begin{aligned} \iint _{\Omega } |\nabla \phi | \left| \ln \Big ( \frac{K u}{|t|} \Big ) \right| dt\, dy \le C r^{n-1}, \end{aligned}$$

where *C* depends only on *n*,  $C_\mathcal {A},$ the 1-sided CAD constants of $\Omega ,$ the constant $C_\phi$ in Definition 7.1, and the intrinsic constants in $\omega _{L^*} \in A_{\infty }(\sigma ).$

### Proof of Lemma 7.12

The first step is to replace *Ku*/*t* by the elliptic measure. Take $$X_0 \in B(x,r) \cap \Omega $$ and $$X_1\in \Omega {\setminus } B(x,4r)$$ to be two corkscrew points for *x* at the scale *r*. If *G*(*Y*, *X*) is the Green function associated to *L* in $$\Omega $$ and $$\{\omega ^X_*\}_{X\in \Omega }$$ is the elliptic measure associated to the adjoint $$L^*,$$ the CFMS estimates (Lemma 2.18) entails, for $$Y \in W_{\Omega }^{**}(Q) \cap B,$$ that

$$\begin{aligned} \frac{u(Y)}{u(X_0)} \approx \frac{G(Y,X_1)}{G(X_0,X_1)} \approx \frac{\ell (Q)}{r} \frac{\sigma (\Delta )}{\sigma (Q)} \frac{\omega _*^{X_1}(Q)}{\omega _*^{X_1}(\Delta )}, \end{aligned}$$

where $$\Delta = B\cap \partial \Omega .$$ Moreover, if $$Y=(y,t)\in {{\,\textrm{supp}\,}}\phi \cap W^{**}_{\Omega }(Q),$$ then $$\ell (Q) \approx |t|$$ by (7.2). Altogether, we have

7.13 $$\begin{aligned} \frac{u(Y)}{|t|} \approx \frac{u(X_0)}{r} \frac{\sigma (\Delta )}{\sigma (Q)} \frac{\omega _*^{X_1}(Q)}{\omega _*^{X_1}(\Delta )} \quad \text {for } Y=(y,t) \in {{\,\textrm{supp}\,}}\phi \cap W_{\Omega }^{**}(Q). \end{aligned}$$

Set $$K := r/u(X_0),$$ and $$I_\phi ':=\left\{ i\in I_\phi : W_\Omega ^{**}(Q_i) \text { intersects }{{\,\textrm{supp}\,}}\nabla \phi \right\} ,$$

7.14 $$\begin{aligned} \iint _{\Omega } |\nabla \phi | \left| \ln \Big ( \frac{K u}{|t|} \Big ) \right| dt\, dy\lesssim & {} \sum _{i\in I_\phi '} \ell (Q_i)^{-1} \int _{W_{\Omega }^{**}(Q_i)} \left| \ln \Big ( \frac{K u}{|t|} \Big ) \right| \, dt\, dy\nonumber \\\lesssim & {} \sum _{i\in I_\phi '} \sigma (Q_i) \left[ 1 + \left| \ln \Big (\frac{\sigma (\Delta )}{\sigma (Q_i)} \frac{\omega _*^{X_1}(Q_i)}{\omega _*^{X_1}(\Delta )} \Big ) \right| \right] \nonumber \\ \end{aligned}$$

by (7.4), (7.13), and the fact that $$|W^{**}_{\Omega }(Q_i)| \approx \ell (Q_i) \sigma (Q_i).$$

The second step is to use the fact that $\omega ^{X_1}_*$ is $A_{\infty }$-absolutely continuous with respect to $\sigma .$ To that objective, we define for $k\in {{\mathbb {Z}}}$

$$\begin{aligned} {\mathcal {I}}_k := \Big \{i \in I_\phi ', \, 2^{k} \le \frac{\sigma (\Delta )}{\sigma (Q_i)} \frac{\omega _*^{X_1}(Q_i)}{\omega _*^{X_1}(\Delta )} \le 2^{k+1}\Big \} \end{aligned}$$

and then $E_k:= \bigcup _{i\in {{\mathcal {I}}}_k} Q_i.$ Since the collection $\{Q_i\}_{i\in I_\phi }$ is finitely overlapping, due to (7.3), the bound (7.14) becomes

7.15 $$\begin{aligned} \iint _{\Omega } |\nabla \phi | \left| \ln \Big ( \frac{K u}{|t|} \Big ) \right| dt\, dy&\lesssim \sum \limits _{k\in {{\mathbb {Z}}}} (1+|k|) \sigma (E_k). \end{aligned}$$

We want thus to estimate $$\sigma (E_k).$$ Observe first that for any $$i\in I_\phi ',$$ $$W_{\Omega }^{**}(Q_i)$$ intersects $${{\,\textrm{supp}\,}}\phi \subset B.$$ Therefore $$Q_i$$ and $$E_k$$ have to be inside $$\Delta ^*:=C\Delta $$ for a large *C* depending only on the constant $$K^{**}$$ in (4.6). The finite overlapping (7.3) also implies that

$$\begin{aligned} \frac{\sigma (\Delta ^*)}{\sigma (E_k)} \frac{\omega _*^{X_1}(E_k)}{\omega _*^{X_1}(\Delta ^*)} \approx 2^k \end{aligned}$$

For $k\ge 0,$ we have

7.16 $$\begin{aligned} \frac{\sigma (E_k)}{\sigma (\Delta ^*)} \approx 2^{-k} \frac{\omega _*^{X_1}(E_k)}{\omega _*^{X_1}(\Delta ^*)} \lesssim 2^{-k}. \end{aligned}$$

The elliptic measure $\omega _*^{X_1}$ is $A_{\infty }$-absolutely continuous with respect to $\sigma$ by assumption, so for $k\le 0,$ we use the characterization (iv) from Theorem 1.4.13 in [43] to deduce

7.17 $$\begin{aligned} \frac{\sigma (E_k)}{\sigma (\Delta ^*)} \lesssim \left( \frac{\omega _*^{X_1}(E_k)}{\omega _*^{X_1}(\Delta ^*)}\right) ^{\theta } \approx 2^{k\theta } \left( \frac{\sigma (E_k)}{\sigma (\Delta ^*)}\right) ^{\theta } \lesssim 2^{k\theta } \end{aligned}$$

for some $\theta \in (0,1)$ independent of *x*,  *r*,  and *k*. We reinject (7.16) and (7.17) in (7.15) to conclude that

$$\begin{aligned} \iint _{\Omega } |\nabla \phi | \left| \ln \Big ( \frac{K u}{|t|} \Big ) \right| dt\, dy&\lesssim \sigma (\Delta ^*) \sum \limits _{k\in {{\mathbb {Z}}}} (1+|k|) 2^{-|k|\theta } \lesssim \sigma (\Delta ^*) \lesssim r^{n-1} \end{aligned}$$

because $\sigma$ is Ahlfors regular. The lemma follows. $\square$

### Proof of Lemma 7.9

The proof is divided in two parts: the first one treats the case where $\mathcal {B}_{i,n}=0$ for $i<n,$ and the second one shows that we can come back to the first case by a change of variable, by adapting the method presented in [32].

Observe that $\phi$ can be decomposed as $\phi =\phi _+ + \phi _-$ where $\phi _+ = {\mathbb {1}}_{t>0} \phi$ and $\phi _- = {\mathbb {1}}_{t<0} \phi .$ Both $\phi _+$ and $\phi _-$ are as in Definition 7.1 with constant $C_\phi .$ So it is enough to prove the lemma while assuming

7.18 $$\begin{aligned} {{\,\textrm{supp}\,}}\phi \subset \{t\ge 0\} = \overline{\mathbb {R}^n_+}. \end{aligned}$$

The proof of the case ${{\,\textrm{supp}\,}}\phi \subset \mathbb {R}^n_-$ is of course identical up to obvious changes.

**Step 1:** Case where ${\mathcal {B}}_{i,n} = 0$ for $i<n$ on ${{\,\textrm{supp}\,}}\phi$ and $\mathcal {B}$ satisfies (1.6) and (1.7) with the same constant $C_\mathcal {A}$ as $\mathcal {A}.$ If $b:= \mathcal {B}_{n,n},$ this assumption on ${\mathcal {B}}$ implies that

7.19 $$\begin{aligned} \mathcal {B}\nabla t \cdot \nabla v \, \phi ^2= b \, \partial _t v \, \phi ^2. \end{aligned}$$

whenever $$v\in W^{1,1}_{loc}(\Omega )$$ and

7.20 $$\begin{aligned} (C_\mathcal {A})^{-1} \le b \le C_\mathcal {A}. \end{aligned}$$

We want to prove (7.11) with the assumption (7.18), and for this, we intend to establish that

7.21 $$\begin{aligned} T:= \iint _{\mathbb {R}^n_+} t\left| \nabla \ln \Big ( \frac{u}{t} \Big ) \right| ^2 \phi ^2 \, dt\, dy \lesssim T^{\frac{1}{2}} r^{\frac{n-1}{2}} + r^{n-1}, \end{aligned}$$

which implies the desired inequality (7.11) provided that *T* is *a priori* finite. However that is not necessary the case, because some problems can occur when *t* is close to 0. So we take $\psi \in C^\infty (\mathbb {R})$ such that $\psi (t) = 0$ when $t<1,$ $\psi (t) = 1$ when $t\ge 2,$ and $0 \le \psi \le 1.$ We construct then $\psi _k(Y) = \psi (2^k\delta (Y))$ and $\phi _k = \phi \psi _k.$ It is not very hard to see that

$$\begin{aligned} {{\,\textrm{supp}\,}}\nabla \psi _k := \{X\in \Omega , \, 2^{-k} \le \delta (X) \le 2^{1-k}\} \subset \bigcup _{Q\in {\mathbb {D}}_k} W_{\Omega }^{**}(Q) \end{aligned}$$

and therefore that $\phi _k$ is as in Definition 7.1 (with $C_{\phi _k} = C_\phi +1)$. The quantity

$$\begin{aligned} T(k) := \iint _{\mathbb {R}^n_+} t \left| \nabla \ln \Big ( \frac{u}{t} \Big ) \right| ^2 \phi _k^2 \, dt\, dy = \iint _{\mathbb {R}^n_+} t \left| \frac{\nabla u}{u} - \frac{\nabla t}{t} \right| ^2 \phi _k^2 \, dt\, dy \end{aligned}$$

is finite, because $\phi _k$ is compactly supported in both $\Omega$ and $\mathbb {R}^{n}_+$ (the fact that $\nabla u/u$ is in $L^2_{loc}(\Omega )$ for a non-negative nontrivial solution to $Lu=0$ is a consequence of the Caccioppoli inequality and the Harnack inequality). So, we prove (7.21) for *T*(*k*) instead of *T*,  which implies $T(k) \lesssim r^{n-1}$ as we said, and take $k\rightarrow \infty$ to deduce (7.11).

We are now ready for the core part of the proof, which can be seen as an elaborate integration by parts. Our previous discussion established that we (only) have to prove (7.21), and that we can assume that $\phi$ is compactly supported in $\Omega \cap \mathbb {R}^{n}_+.$ We use the ellipticity of $\mathcal {A}$ and the boundedness of *b* to write

$$\begin{aligned} T= & {} \iint _{\mathbb {R}^n_+} t \left| \frac{\nabla u}{u} - \frac{\nabla t}{t}\right| ^2 \phi ^2 \, dt\, dy\\\le & {} C_{\mathcal {A}}^2\iint _{\mathbb {R}^n_+}\frac{\mathcal {A}}{b}\left( \frac{\nabla u}{u}-\frac{\nabla t}{t}\right) \cdot \left( \frac{\nabla u}{u}-\frac{\nabla t}{t}\right) \phi ^2dtdy\\= & {} C_{\mathcal {A}}^2\left( \iint _{\mathbb {R}^n_+} \frac{\mathcal {A}\nabla u}{bu} \cdot \left( \frac{\nabla u}{u} - \frac{\nabla t}{t} \right) \, t\phi ^2 \, dt\, dy - \iint _{\mathbb {R}^n_+} \frac{\mathcal {A}\nabla t}{bt} \cdot \nabla \ln \Big ( \frac{u}{t}\Big ) \, t\phi ^2 \, dt\, dy\right) \\:= & {} C_{\mathcal {A}}^2( T_1 + T_2). \end{aligned}$$

We deal first with $T_2.$ We use the fact that $\mathcal {A}= \mathcal {B}+ {\mathcal {C}}$ and (7.19) to obtain

$$\begin{aligned} T_2 = - \iint _{\mathbb {R}^n_+} \partial _t \ln \Big ( \frac{u}{t}\Big ) \, \phi ^2 \, dt\, dy - \iint _{\mathbb {R}^n_+} \frac{{\mathcal {C}}}{b} \nabla t \cdot \nabla \ln \Big ( \frac{u}{t}\Big ) \, \phi ^2 \, dt\, dy := T_{21} + T_{22}. \end{aligned}$$

The term $T_{22}$ can be then bounded with the help of the Cauchy–Schwarz inequality as follows

$$\begin{aligned} T_{22} \le \Vert b^{-1}\Vert _{\infty } \left( \iint _{\mathbb {R}^{n}_+} |{\mathcal {C}}|^2\phi ^2 \, \frac{dt}{t} \, dy \right) ^\frac{1}{2} \left( \iint _{\mathbb {R}^{n}_+} t \left| \nabla \ln \Big ( \frac{u}{t}\Big )\right| ^2 \, \phi ^2 \, dt \, dy \right) ^\frac{1}{2} \lesssim r^{\frac{n-1}{2}}T^{\frac{1}{2}} \end{aligned}$$

by (7.10). As for $T_{21},$ observe that multiplying by any constant *K* inside the logarithm will not change the term (because we differentiate the logarithm). As a consequence, we have

$$\begin{aligned} T_{21}= & {} - \iint _{\mathbb {R}^{n}_+} \partial _t \ln \Big ( \frac{Ku}{t}\Big ) \, \phi ^2 \, dt\, dy = \iint _{\mathbb {R}^{n}_+} \ln \Big ( \frac{Ku}{t}\Big ) \, \partial _n [\phi ^2] \, dt\, dy \\\le & {} \iint _{\mathbb {R}^{n}_+} |\nabla \phi | \left| \ln \Big ( \frac{Ku}{t}\Big )\right| \, dt\, dy \lesssim r^{n-1} \end{aligned}$$

by successively using integration by parts and Lemma 7.12.

We turn to $T_1,$ and we want now to use the fact that *u* is a weak solution to $Lu = 0.$ So we notice that

$$\begin{aligned} T_1= & {} - \iint _{\mathbb {R}^{n}_+} \frac{\mathcal {A}}{b} \nabla u \cdot \nabla \Big ( \frac{t}{u} \Big ) \, \phi ^2 \, dt\, dy \\= & {} - \iint _{\mathbb {R}^{n}_+} \mathcal {A}\nabla u \cdot \nabla \Big ( \frac{t\phi ^2}{bu} \Big ) \, \, dt\, dy + 2 \iint _{\mathbb {R}^{n}_+} \mathcal {A}\nabla u \cdot \nabla \phi \, \Big (\frac{t\phi }{bu} \Big ) \, dt\, dy \\{} & {} - \iint _{\mathbb {R}^n_+} \mathcal {A}\nabla u \cdot \nabla b \, \Big (\frac{t\phi ^2}{b^2u} \Big ) \, dt\, dy := - T_{11} + 2 T_{12} - T_{13}. \end{aligned}$$

Since $\phi$ is compactly supported, we have that $u > \epsilon _\phi$ on ${{\,\textrm{supp}\,}}\phi$ (by the Harnack inequality, see Lemma 2.15) and $\nabla u\in L^2_{loc}(\Omega )$ (by the Caccioppoli inequality, see Lemma 2.14). Therefore $t\phi ^2/(bu)$ is a valid test function for the solution $u\in W^{1,2}_{loc}(\Omega )$ to $Lu=0,$ and then $T_{11} = 0.$ As for $T_{12},$ we have

$$\begin{aligned} T_{12}= & {} \iint _{\mathbb {R}^{n}_+} \frac{\mathcal {A}}{b} \left( \frac{\nabla u}{u} -\frac{\nabla t}{t} \right) \cdot \nabla \phi \, (t\phi ) \, dt\, dy + \iint _{\mathbb {R}^{n}_+} \frac{\mathcal {A}}{b} \nabla t \cdot \nabla \phi \, \phi \, dt\, dy \\:= & {} T_{121} + T_{122}. \end{aligned}$$

The term $T_{121}$ is similar to $T_{22}.$ The boundedness of $\mathcal {A}/b$ and the Cauchy–Schwarz inequality infer that

$$\begin{aligned} T_{121} \le \left( \iint _{\mathbb {R}^{n}_+} t|\nabla \phi |^2 \, \frac{dt}{t} \, dy \right) ^\frac{1}{2} \left( \iint _{\mathbb {R}^{n}_+} t \left| \nabla \ln \Big ( \frac{u}{t}\Big )\right| ^2 \, \phi ^2 \, dt \, dy \right) ^\frac{1}{2} \lesssim r^{\frac{n-1}{2}}T^{\frac{1}{2}} \end{aligned}$$

by (7.8). The quantity $T_{122}$ is even easier since

$$\begin{aligned} T_{122} \lesssim \iint _{\mathbb {R}^{n}_+} |\nabla \phi | \, dt\, dy \lesssim r^{n-1}, \end{aligned}$$

again by (7.8). It remains to bound $T_{13}.$ We start as for $T_{12}$ by writing

$$\begin{aligned} T_{13}= & {} \iint _{\mathbb {R}^{n}_+} {\mathcal {A}} \left( \frac{\nabla u}{u} - \frac{\nabla t}{t} \right) \cdot \nabla b \, \frac{t\phi ^2}{b^2} \, dt\, dy + \iint _{\mathbb {R}^{n}_+} {\mathcal {A}} \nabla t \cdot \nabla b \, \frac{\phi ^2}{b^2} \, dt\, dy \\:= & {} T_{131} + T_{132}. \end{aligned}$$

The term $T_{131}$ is like $T_{121},$ and by using $t\nabla b \in CM_{\mathbb {R}^n_+}$ instead of $t\nabla \phi \in CM_{\mathbb {R}^n_+}$ , we obtain that $T_{131} \lesssim r^{(n-1)/2}T^{1/2}.$ The term $T_{132}$ does not contain the solution *u*,  but it is a bit harder than $T_{122}$ to deal with, because $\nabla b$ is not as nice as $\nabla \phi .$ We use ${\mathcal {A}} = {\mathcal {B}} + {\mathcal {C}}$ and (7.19) to get

$$\begin{aligned} T_{132} = \iint _{\mathbb {R}^{n}_+} (\partial _t b) \, \frac{\phi ^2}{b} \, dt\, dy + \iint _{\mathbb {R}^{n}_+} {\mathcal {C}} \nabla t \cdot \nabla b \, \frac{\phi ^2}{b^2} \, dt\, dy := T_{1321} + T_{1322}. \end{aligned}$$

We easily deal with $T_{1322}$ by using the Cauchy–Schwarz inequality as follows:

$$\begin{aligned} T_{1322} \le \Vert b^{-1}\Vert _{\infty }^2 \left( \iint _{\mathbb {R}^{n}_+} |{\mathcal {C}}|^2 \phi ^2 \, \frac{dt}{t} \, dy \right) ^\frac{1}{2} \left( \iint _{\mathbb {R}^{n}_+} |t\nabla b|^2 \phi ^2 \, \frac{dt}{t} \, dy\right) ^\frac{1}{2} \lesssim r^{n-1} \end{aligned}$$

by (7.10). As last, observe that

$$\begin{aligned} T_{1321} = \iint _{\mathbb {R}^{n}_+} \partial _t [\ln (b)\phi ^2] \, \, dt\, dy - \iint _{\mathbb {R}^{n}_+} \partial _t \phi \, \phi \ln (b) \, dt\, dy, \end{aligned}$$

but the first integral in the right-hand side above is zero, so

$$\begin{aligned} |T_{1321}| \lesssim \Vert \ln (b)\Vert _{\infty } \iint _{\mathbb {R}^{n}_+} |\partial _t \phi | \, dt\, dy \lesssim r^{n-1}, \end{aligned}$$

by (7.8) and the fact that $b\approx 1.$ The inequality (7.11) under the three assumptions (7.19), (7.20), and (7.10) follows.

**Step 2: We can assume that** $\left\| t \left| \nabla _y\mathcal {B}\right| \right\| _{\infty }$ **is as small as we want.**

We construct

7.22 $$\begin{aligned} \widetilde{{\mathcal {A}}} := {\mathcal {A}} \phi + (1-\phi ) I, \end{aligned}$$

where *I* is the identity matrix. Note that $\widetilde{{\mathcal {A}}}$ is elliptic with the same elliptic constant $C_\mathcal {A}$ as ${\mathcal {A}}.$ We choose then a bump function $\theta \in C^\infty _0(\mathbb {R}^n)$ supported in *B*(0, 1/10),  that is $0 \le \theta \le 1$ and $\iint _{\mathbb {R}^n} \theta \, dX = 1.$ We construct $\theta _{y,t}(z,s) = t^{-n}\theta \big (\frac{z-y}{t},\frac{s-t}{t}\big ),$ which satisfies $\iint _{\mathbb {R}^n} \theta _{y,t} = 1,$ and then

7.23 $$\begin{aligned} \widetilde{{\mathcal {B}}}(y,t) := \iint _{\mathbb {R}^n} \widetilde{{\mathcal {A}}} \, \theta _{y,Nt} \, dz\, ds. \end{aligned}$$

for a large *N* to be fixed later to ensure that (7.28) below is invertible. Since $\widetilde{{\mathcal {B}}}$ is some average of $\widetilde{{\mathcal {A}}},$ then

7.24 $$\begin{aligned} \widetilde{{\mathcal {B}}}\text { is elliptic and bounded with the same constant }C_{\mathcal {A}}\text { as }\widetilde{{\mathcal {A}}}\text { and }{\mathcal {A}}. \end{aligned}$$

The construction is similar to the one done in Lemma 2.1, so we do not write the details again. Observe also that

7.25 $$\begin{aligned} |t \nabla _y \widetilde{{\mathcal {B}}}(y,t)| \lesssim \frac{1}{N} \Vert \widetilde{{\mathcal {A}}}\Vert _{\infty } \quad \text { and } \quad |t\, \partial _t \widetilde{{\mathcal {B}}}(y,t)| \lesssim \Vert \widetilde{{\mathcal {A}}}\Vert _{\infty }. \end{aligned}$$

In addition, we have that

$$\begin{aligned} |\nabla \widetilde{{\mathcal {B}}}(y,t)| \lesssim t^{-n} \iint _{B_{Nt/10}(y,Nt)} \left( |\nabla {\mathcal {B}}| \phi + |\nabla \phi | + \frac{1}{t} |{\mathcal {C}}| \phi \right) dz\, ds, \end{aligned}$$

and if $\widetilde{{\mathcal {C}}}$ denotes $({\mathcal {A}} - \widetilde{{\mathcal {B}}}) {\mathbb {1}}_{{{\,\textrm{supp}\,}}\phi },$ the Poincaré inequality entails that

$$\begin{aligned}{} & {} \int _{\Delta (x,t)} \int _{t}^{3t} |\widetilde{{\mathcal {C}}}(z,s)|^2 \frac{ds}{s}\, dz \\{} & {} \quad \lesssim \int _{\Delta (x,2Nt)} \int _{t}^{9Nt} \Big ( s^2 |\nabla {\mathcal {B}}|^2 \phi ^2 + |{\mathcal {C}}|^2 \phi ^2 + s^2 |\nabla \phi |^2 + |{\mathbb {1}}_{({{\,\textrm{supp}\,}}\phi ) \cap {{\,\textrm{supp}\,}}(1-\phi )}|^2 \Big ) \frac{ds}{s}\, dz, \end{aligned}$$

which means that $t|\nabla \widetilde{{\mathcal {B}}}| + |\widetilde{{\mathcal {C}}}| \in CM_{\mathbb {R}^n_+}$ by (7.10), and (7.7).

**Step 3: The change of variable.** We write $\widetilde{{\mathcal {B}}}$ as the block matrix

7.26 $$\begin{aligned} \widetilde{{\mathcal {B}}} = \begin{pmatrix} B_1 &{} B_2 \\ B_3 &{} b \end{pmatrix}, \end{aligned}$$

where *b* is the scalar function $\widetilde{{\mathcal {B}}}_{n,n},$ so $B_1$ is a matrix of order $n-1,$ $B_2$ and $B_3$ are respectively a vertical and a horizontal vector of length $n-1.$ We use *v* for the horizontal vector $v = - (B_2)^T/b,$ and we define

7.27 $$\begin{aligned} \rho (y,t) := (y+t v(y,t), t), \end{aligned}$$

which is a Lipschitz map from $\mathbb {R}^n_+$ to $\mathbb {R}^n_+$ (since *v* and $t |\nabla v|$ are uniformly bounded, see (7.24) and (7.25)), and we compute its Jacobian

7.28 $$\begin{aligned} Jac_\rho := \begin{pmatrix} I + t\nabla _y v &{} 0 \\ v+t\partial _t v &{} 1 \end{pmatrix}. \end{aligned}$$

We can choose *N* big enough in (7.25) such that $Jac_\rho$ is invertible and even $\det (Jac_\rho ) \ge 1/2.$ Let $J_\rho$ be the matrix

7.29 $$\begin{aligned} J_\rho := \begin{pmatrix} I &{} 0 \\ v &{} 1 \end{pmatrix}. \end{aligned}$$

We easily have that

7.30 $$\begin{aligned} |Jac_\rho - J_\rho | + |\det (Jac_{\rho })^{-1} - 1| \lesssim |t\nabla v| \lesssim |t\nabla \widetilde{{\mathcal {B}}}| . \end{aligned}$$

We aim to use $\rho$ for a change of variable. If *u* is a weak solution to $L=-{{\,\textrm{div}\,}}{\mathcal {A}} \nabla ,$ then $u\circ \rho ^{-1}$ is solution to $L_\rho = -{{\,\textrm{div}\,}}({\mathcal {A}}_\rho \circ \rho ^{-1}) \nabla$ where

7.31 $$\begin{aligned} {\mathcal {A}}_\rho = \det (Jac_\rho )^{-1} (Jac_\rho )^T {\mathcal {A}} \, Jac_\rho . \end{aligned}$$

We want to compute ${\mathcal {A}}_\rho .$ To lighten the notation, we write ${\mathcal {O}}_{CM}$ for a scalar function, a vector, or a matrix that satisfies the Carleson measure condition with respect to $\mathbb {R}^{n}_+,$ i.e. ${\mathcal {O}}_{CM}$ can change from one line to another as long as ${\mathcal {O}}_{CM} \in CM_{\mathbb {R}^n_+}.$ So (7.30) becomes

7.32 $$\begin{aligned} Jac_\rho = J_\rho + {\mathcal {O}}_{CM} \quad \text {and} \quad \det (Jac_\rho )^{-1} = 1 + {\mathcal {O}}_{CM}. \end{aligned}$$

Remember that by construction, the matrix ${\mathcal {A}}$ equals $\widetilde{{\mathcal {B}}} + \widetilde{{\mathcal {C}}} = \widetilde{{\mathcal {B}}} + {\mathcal {O}}_{CM}$ on ${{\,\textrm{supp}\,}}\phi ,$ and that ${{\,\textrm{Jac}\,}}_\rho$ and $\mathcal {A}$ are uniformly bounded, so

7.33 $$\begin{aligned} \begin{aligned} ({\mathbb {1}}_{{{\,\textrm{supp}\,}}\phi }) {\mathcal {A}}_\rho&= {\mathbb {1}}_{{{\,\textrm{supp}\,}}\phi } \begin{pmatrix} I &{} v^T \\ 0 &{} 1 \end{pmatrix} \begin{pmatrix} B_1 &{} B_2 \\ B_3 &{} b \end{pmatrix} \begin{pmatrix} I &{} 0 \\ v &{} 1 \end{pmatrix} + {\mathcal {O}}_{CM} \\&= {\mathbb {1}}_{{{\,\textrm{supp}\,}}\phi } \begin{pmatrix} B_1 + v^TB_3 + B_2v + bvv^T &{} B_2+bv^T \\ B_3 +bv &{} b \end{pmatrix} + {\mathcal {O}}_{CM} \\&= {\mathbb {1}}_{{{\,\textrm{supp}\,}}\phi } \underbrace{\begin{pmatrix} b(B_1 + v^TB_3 + B_2v + bvv^T) &{} 0 \\ B_3-(B_2)^T &{} b \end{pmatrix}}_{:={\mathcal {B}}_\rho } + {\mathcal {O}}_{CM} \end{aligned} \nonumber \\ \end{aligned}$$

with our choices of *v*. We write ${\mathcal {C}}_\rho$ for $({\mathcal {A}}_\rho - {\mathcal {B}}_\rho ){\mathbb {1}}_{{{\,\textrm{supp}\,}}\phi } = {\mathcal {O}}_{CM}.$ The matrices ${\mathcal {B}}_\rho \circ \rho ^{-1}$ and ${\mathcal {C}}_\rho \circ \rho ^{-1}$ satisfy (7.10) (because the Carleson measure condition is stable under bi-Lipschitz transformations) and ${\mathcal {B}}_\rho \circ \rho ^{-1}$ has the structure (7.19) as in Step 1. So Step 1 gives that

7.34 $$\begin{aligned} \iint _{\mathbb {R}^{n}_+} s \left| \nabla \ln \Big ( \frac{u\circ \rho ^{-1}}{s} \Big ) \right| ^2 \phi ^2\circ \rho ^{-1} \, ds\, dz \lesssim r^{n-1}. \end{aligned}$$

If *s* (and *t*) is also used, by notation abuse, for the projection on the last coordinate, then

$$\begin{aligned} \iint _{\mathbb {R}^{n}_+} t \left| \nabla \ln \Big ( \frac{u}{t} \Big ) \right| ^2 \phi ^2 \, dt\, dy= & {} \iint _{\mathbb {R}^{n}_+} t \left| \frac{\nabla u}{u} - \frac{\nabla t}{t} \right| ^2 \phi ^2 \, dt\, dy \\= & {} \iint _{\mathbb {R}^{n}_+} t \left| \frac{Jac_\rho \nabla (u\circ \rho ^{-1}) \circ \rho }{u} - \frac{\nabla t}{t} \right| ^2 \phi ^2 \, dt\, dy \\\le & {} \iint _{\mathbb {R}^{n}_+} t \left| \frac{Jac_\rho \nabla (u\circ \rho ^{-1}) \circ \rho }{u} - \frac{Jac_\rho (\nabla s)\circ \rho }{s\circ \rho } \right| ^2 \phi ^2 \, dt\, dy \\{} & {} + \iint _{\mathbb {R}^{n}_+} t \left| \frac{Jac_\rho (\nabla s)\circ \rho }{s\circ \rho } - \frac{\nabla t}{t} \right| ^2 \phi ^2 \, dt\, dy := I_1 + I_2. \end{aligned}$$

Yet, $\rho$ is a bi-Lipschitz change of variable, so $Jac_\rho$ and $\det (Jac_\rho )^{-1}$ are uniformly bounded, and we have

7.35 $$\begin{aligned} I_1\lesssim & {} \iint _{\mathbb {R}^{n}_+} t \left| \frac{\nabla (u\circ \rho ^{-1}) \circ \rho }{u} - \frac{(\nabla s)\circ \rho }{s\circ \rho } \right| ^2 \phi ^2 \, dt\, dy \nonumber \\\lesssim & {} \iint _{\mathbb {R}^{n}_+} s \left| \frac{\nabla (u\circ \rho ^{-1})}{u \circ \rho ^{-1}} - \frac{\nabla s}{s}\right| ^2 \phi ^2\circ \rho ^{-1} \, ds\, dz \nonumber \\= & {} \iint _{\mathbb {R}^{n}_+} s \left| \nabla \ln \Big ( \frac{u\circ \rho ^{-1}}{s} \Big ) \right| ^2 \phi ^2\circ \rho ^{-1} \, ds\, dz \lesssim r^{n-1} \end{aligned}$$

by (7.34). As for $I_2,$ we simply observe that $s\circ \rho = t$ and

$$\begin{aligned} Jac_\rho (\nabla s)\circ \rho = \nabla t\end{aligned}$$

to deduce that $I_2 = 0.$ The lemma follows. $\square$

## Proof of Theorem 1.21

In this section we prove Theorem 1.21, using the same strategy as our proof of Theorem 1.12. As mentioned in the introduction, we shall explain how to change the 5-step sketch of proof given in Sect. 1.3 to prove Theorem 1.21.

Fix a bounded solution *u* of $Lu=0$ in $\Omega$ with $\left\| u\right\| _{L^\infty (\Omega )}\le 1$ and a ball $B=B(x_0,r)$ centered on $\partial \Omega$ with radius *r*. By the same argument as Step 1 in Sect. 1.3, it suffices to show that there exists some constant $C\in (0,\infty )$ depending only on *n*,  *M* and the UR constants of $\partial \Omega ,$ such that

8.1 $$\begin{aligned} I := \sum _{Q\in {\mathbb {D}}_{\partial \Omega }(Q_0)}\iint _{W_\Omega (Q)}\left| \nabla u(X)\right| ^2\delta (X)dX\le C\sigma (Q_0) \end{aligned}$$

for any cube $Q_0\in {\mathbb {D}}_{\partial \Omega }$ that satisfies $Q_0\subset \frac{8}{7}B\cap \partial \Omega$ and $\ell (Q_0)\le 2^{-8}r.$

Then observe that if $E\subset \Omega$ is a Whitney region, that is, $E\subset \frac{7}{4} B$ and ${{\,\textrm{diam}\,}}(E)\le K\delta (E),$ then

8.2 $$\begin{aligned} \iint _E\left| \nabla u\right| ^2\delta \, dX\le C_K{{\,\textrm{diam}\,}}(E)^{-1}\iint _{E^*}\left| u\right| ^2dX\le C_K\delta (E)^{n-1}, \end{aligned}$$

by the Caccioppoli inequality and $\left\| u\right\| _{L^\infty (\Omega )}\le 1,$ where $E^*$ is an enlargement of *E*. This bound (8.2) is the analogue of (1.26), and proves Step 2.

Step 3 is not modified. We pick $0 <\epsilon _1\ll \epsilon _0 \ll 1$ and we use the corona decomposition constructed in Sect. 3 to decompose *I* as follows.

$$\begin{aligned} I=\sum _{Q\in \mathcal {B}(Q_0)}\iint _{W_\Omega (Q)}\left| \nabla u\right| ^2\delta \, dX+\sum _{\mathcal {S}\in {\mathfrak {S}}(Q_0)}\iint _{W_\Omega (\mathcal {S})}\left| \nabla u\right| ^2\delta \, dX=: I_1+\sum _{\mathcal {S}\in {\mathfrak {S}}(Q_0)} I_{\mathcal {S}}. \end{aligned}$$

By (8.2) and (3.19),

$$\begin{aligned} I_1\le C\sum _{Q\in \mathcal {B}(Q_0)}\ell (Q)^{n-1}\le C\sigma (Q_0). \end{aligned}$$

Step 4 is significantly simpler for Theorem 1.21, because we do not need any estimate on the smooth distance $D_{\beta },$ but the spirit is the same. That is, by using the bi-Lipschitz map $\rho _{\mathcal {S}}$ constructed in Sect. 6, $I_{\mathcal {S}}$ can be turned into an integral on $\mathbb {R}^n {\setminus } \mathbb {R}^{n-1},$ which can be estimated by an integration by parts argument. More precisely, for any fixed $\mathcal {S}\in {\mathfrak {S}}(Q_0),$

$$\begin{aligned} I_{\mathcal {S}}= & {} \iint _{\rho _{\mathcal {S}}^{-1}(W_\Omega (\mathcal {S}))}\left| (\nabla u)\circ \rho _{\mathcal {S}}(p,t)\right| ^2\delta \circ \rho _{\mathcal {S}}(p,t)\det {{\,\textrm{Jac}\,}}(p,t)dpdt\\\le & {} 2\iint \left| \nabla ( u\circ \rho _{\mathcal {S}}(p,t))\right| ^2{{\,\textrm{dist}\,}}(\rho _{\mathcal {S}}(p,t),\Gamma _{\mathcal {S}})\left( \Psi _{\mathcal {S}}\circ \rho _{\mathcal {S}}(p,t)\right) ^2dpdt\\\le & {} 3\iint \left| \nabla v(p,t)\right| ^2\left| t\right| \phi (p,t)^2dpdt, \qquad v=u\circ \rho _{\mathcal {S}}, \quad \phi =\Psi _{\mathcal {S}}\circ \rho _{\mathcal {S}} \end{aligned}$$

by (4.15), Lemmata 4.1(d) and 6.8, as well as (6.12), for $\epsilon _0$ sufficiently small.

The fifth step consists roughly in proving the result in $\mathbb {R}^n {\setminus } \mathbb {R}^{n-1}.$ The function $\phi$ is the same as the one used to prove Theorem 1.12, in particular it is a cutoff function associated to both $\rho _{\mathcal {S}}^{-1}(\partial \Omega )$ and $\mathbb {R}^{n-1}$ as defined in Definition 7.1, and it satisfies

8.3 $$\begin{aligned} {{\,\textrm{supp}\,}}\phi \subset \rho _{\mathcal {S}}^{-1}(W_\Omega ^*(\mathcal {S})) , \end{aligned}$$

and

8.4 $$\begin{aligned} \iint \left| \nabla \phi \right| dtdp+\iint \left| \nabla \phi \right| ^2tdtdp\lesssim \sigma (Q(\mathcal {S})), \end{aligned}$$

where the implicit constant depends on *n* and the AR constant in (1.1). Notice that $v=u\circ \rho _{\mathcal {S}}$ is a bounded solution of $L_{\mathcal {S}}=-{{\,\textrm{div}\,}}\mathcal {A}_{\mathcal {S}}\nabla$ that satisfies $\left\| v\right\| _{L^\infty }\le 1,$ where $\mathcal {A}_{\mathcal {S}}$ is defined in (6.18). By Lemma 6.20, $I_{\mathcal {S}}\le C\sigma (Q(\mathcal {S}))$ will follow from the following lemma, which is essentially a result in $\mathbb {R}^n {\setminus } \mathbb {R}^{n-1}.$

### Lemma 50

Let $L=-{{\,\textrm{div}\,}}{\mathcal {A}} \nabla$ be a uniformly elliptic operator on $\Omega _{\mathcal {S}}:=\rho ^{-1}_{\mathcal {S}}(\Omega ).$ Assume that the coefficients $\mathcal {A}$ can be decomposed as ${\mathcal {A}} = {\mathcal {B}} + {\mathcal {C}}$ where

8.6 $$\begin{aligned} (|t\nabla {\mathcal {B}}| + |{\mathcal {C}}|){\mathbb {1}}_{{{\,\textrm{supp}\,}}\phi } \in CM_{\mathbb {R}^n {\setminus }\mathbb {R}^{n-1}}(M), \end{aligned}$$

where $\phi =\Psi _{\mathcal {S}}\circ \rho _{\mathcal {S}}$ is as above. Then for any solution *v* of $Lv=0$ in $\rho ^{-1}_{\mathcal {S}}(\Omega )$ that satisfies $\left\| v\right\| _{L^\infty }\le 1,$ there holds

8.7 $$\begin{aligned} \iint _{ \mathbb {R}^n {\setminus } \mathbb {R}^{n-1}} \left| \nabla v\right| ^2\phi ^2 { |t|}\,dtdy\le C(1+M) \sigma (Q(\mathcal {S})), \end{aligned}$$

where *C* depends only on the dimension *n*,  the elliptic constant $C_\mathcal {A},$ the AR constant of $\partial \Omega ,$ and the implicit constant in (8.4).

The proof of this lemma is similar to the proof of Lemma 7.9, except that there is no need to invoke the CFMS estimates and $A_{\infty }$ as in Lemma 7.12, essentially because *v* is bounded and we do not need information of *v* on the boundary. For the same reason, with the properties of the cutoff function $\phi$ in mind, we can forget about the domain $\Omega _{\mathcal {S}},$ and in particular, we do not need the corkscrew and Harnack chain conditions in the proof.

### Proof of Lemma 8.5

We can decompose $\phi = \phi \,{\mathbb {1}}_{t>0} + \phi \,{\mathbb {1}}_{t<0} := \phi _+ + \phi _-$ and prove the result for each of the functions $\phi _+$ and $\phi _- ,$ and since the proof is the same in both cases (up to a sign), we can restrain ourselves as in the proof of Lemma 7.9 to the case where $\phi = \phi {\mathbb {1}}_{t>0}.$ By an approximation argument as in Step 1 of the proof of Lemma 7.9, we can assume that $T:=\iint _{\mathbb {R}^n_+}\left| \nabla v\right| ^2t\phi ^2dydt$ is finite, and that $\phi$ is compactly supported in $\Omega \cap \mathbb {R}^n_+.$ We first assume that $\mathcal {B}$ has the special structure that

8.8 $$\begin{aligned} \mathcal {B}_{n i}=0\quad \text {for all } 1\le i\le n-1, \quad \mathcal {B}_{nn}=b. \end{aligned}$$

Then for any $f\in W_0^{1,2}(\mathbb {R}^n_+),$

8.9 $$\begin{aligned} \iint \frac{\mathcal {B}}{b}\nabla f\cdot \nabla t\,dydt=\iint \partial _t f\,dydt=0. \end{aligned}$$

Using ellipticity of $\mathcal {A}$ and boundedness of *b*,  we write

$$\begin{aligned} T\le & {} C_{\mathcal {A}}^2\iint \frac{\mathcal {A}}{b}\nabla v\cdot \nabla v\,\phi ^2t\,dydt\\= & {} C_{\mathcal {A}}^2\Big \{\iint \mathcal {A}\nabla v\cdot \nabla \left( v\phi ^2 b^{-1}t\right) dydt-\iint \mathcal {A}\nabla v\cdot \nabla \left( \phi ^2b^{-1}\right) vt\,dydt \\{} & {} -\iint \mathcal {A}\nabla v\cdot \nabla t\,v\phi ^2b^{-1}dydt\Big \}\\= & {} -C_\mathcal {A}^2\Big \{\iint \mathcal {A}\nabla v\cdot \nabla \left( \phi ^2b^{-1}\right) vt\,dydt+\iint \mathcal {A}\nabla v\cdot \nabla t\,v\phi ^2b^{-1}dydt\Big \} \\=: & {} -C_\mathcal {A}^2\left( T_1+T_2\right) \end{aligned}$$

since $Lv=0.$ We write $T_1$ as

$$\begin{aligned} T_1=2\iint \mathcal {A}\nabla v\cdot \nabla \phi \, \phi b^{-1}vt \,dydt-\iint \mathcal {A}\nabla v\cdot \nabla b \,\phi ^2 b^{-2}vt\,dydt=:T_{11}-T_{12}. \end{aligned}$$

By Cauchy–Schwarz and Young’s inequalities, as well as the boundedness of *v* and *b*, 

$$\begin{aligned} \left| T_{11}\right| \le \frac{C_\mathcal {A}^{-2}}{6} T+ C\iint \left| \nabla \phi \right| ^2t\,dydt, \quad \left| T_{12}\right| \le \frac{C_\mathcal {A}^{-2}}{8} T+ C\iint \left| \nabla b\right| ^2t\phi ^2dtdy. \end{aligned}$$

So (8.4) and (8.6), as well as (8.3) give that

$$\begin{aligned} \left| T_1\right| \le \frac{C_\mathcal {A}^2}{4} T + C\ell (Q(\mathcal {S}))^{n-1}. \end{aligned}$$

For $T_2,$ we write

$$\begin{aligned} T_2= & {} \frac{1}{2}\iint \frac{\mathcal {A}}{b}\nabla \left( v^2\phi ^2\right) \cdot \nabla t\,dydt -\iint \frac{\mathcal {A}}{b}\nabla \phi \cdot \nabla t\,v^2\phi \,dydt\\= & {} \frac{1}{2}\iint \frac{\mathcal {C}}{b}\nabla \left( v^2\phi ^2\right) \cdot \nabla t\,dydt -\iint \frac{\mathcal {A}}{b}\nabla \phi \cdot \nabla t\,v^2\phi \,dydt=:T_{21}+T_{22} \end{aligned}$$

by writing $\mathcal {A}=\mathcal {B}+\mathcal {C}$ and applying (8.9). For $T_{21},$ we use Cauchy–Schwarz and Young’s inequalities, and get

$$\begin{aligned} \left| T_{21}\right|\le & {} \left| \iint \mathcal {C}\nabla v\cdot \nabla t\,v\phi ^2b^{-1}dydt\right| +2\left| \iint \mathcal {C}\nabla \phi \cdot \nabla t\,v^2\phi b^{-1}dydt\right| \\\le & {} \frac{C_\mathcal {A}^{-2}}{4}T+ C\iint \left| \mathcal {C}\right| ^2\phi ^2t^{-1}dydt +\iint \left| \nabla \phi ^2\right| tdydt\le \frac{C_\mathcal {A}^{-2}}{4}T+ C\ell (Q(\mathcal {S}))^{n-1} \end{aligned}$$

by the boundedness of *v*,  (8.4), (8.6), and (8.3). The boundedness of the coefficients and *v* implies that

$$\begin{aligned} \left| T_{22}\right| \le C\iint \left| \nabla \phi \right| dydt\le C\ell (Q(\mathcal {S}))^{n-1} \end{aligned}$$

by (8.4). Altogether, we have obtained that $T\le \frac{1}{2} T+ C\ell (Q(\mathcal {S}))^{n-1},$ and thus the desired estimate (8.7) follows.

We claim that the lemma reduces to the case when (8.8) holds by almost the same argument as in Steps 2 and 3 in the proof of Lemma 7.9. That is, we can assume that $\left\| \left| \nabla _y\mathcal {B}\right| t\right\| _{\infty }\lesssim \frac{C_\mathcal {A}}{N}$ with *N* to be chosen to be sufficiently large, and then we do a change of variables, which produces the structure (8.8) in the conjugate operator. The only difference from the proof of Lemma 7.9 is that now we need to choose $v=- B_3/b$ in the bi-Lipschitz map $\rho$ defined in (7.27) because we want $B_3+bv=0$ in (7.33). We leave the details to the reader. $\square$

## The converse

In this section, we show that $(\text {v}) \implies (\text {i})$ in Theorem 1.12, that is, we establish that under certain conditions on the domain $\Omega$ and the operator *L*,  the Carleson condition (1.14) on the Green function implies that $\partial \Omega$ is uniformly rectifiable. More precisely, we prove the following.

### Theorem 51

Let $\Omega$ be a 1-sided Chord-Arc Domain (bounded or unbounded) and let $L=-\mathop {{\text {div}}}\mathcal {A}\nabla$ be a uniformly elliptic operator which satisfies the weak DKP condition with constant $M\in (0,\infty )$ on $\Omega .$ Let $X_0\in \Omega ,$ and when $\Omega$ is unbounded,  $X_0$ can be $\infty .$ We write $G^{X_0}$ for the Green function of *L* with pole at $X_0.$ Suppose that there exists $C\in (0,\infty )$ and $\beta >0$ such that for all balls *B* centered at the boundary and such that $X_0 \notin 2B,$ we have

9.2 $$\begin{aligned} \iint _{\Omega \cap B} \left| \frac{\nabla G^{X_0}}{G^{X_0}} - \frac{\nabla D_{\beta }}{D_{\beta }} \right| ^2 D_{\beta } \, dX \le C \sigma (B\cap \partial \Omega ). \end{aligned}$$

Then $\partial \Omega$ is uniformly rectifiable.

In [30, Theorem 7.1], uniform rectifiability is obtained from some weak condition on the Green function, namely, $G^\infty$ being prevalently close to $D_{\beta }.$ Following [30], we say that $G^\infty$ is prevalently close to $D_{\beta }$ if for each choice of $\epsilon >0$ and $M\ge 1,$ the set ${\mathcal {G}}_{GD_{\beta }}(\epsilon ,M)$ of pairs $(x,r)\in \partial \Omega \times (0,\infty )$ such that there exists a positive constant $c>0,$ with

$$\begin{aligned} \left| D_{\beta }(X)-c\,G^\infty (X)\right| \le \epsilon r \quad \text {for }X\in \Omega \cap B(x,Mr), \end{aligned}$$

is *Carleson-prevalent*.

### Definition 52

(*Carleson-prevalent*) We say that ${\mathcal {G}}\subset \partial \Omega \times (0,\infty )$ is a Carleson-prevalent set if there exists a constant $C\ge 0$ such that for every $x\in \partial \Omega$ and $r>0,$

$$\begin{aligned} \int _{y\in \partial \Omega \cap B(x,r)}\int _{0<t<r}{\mathbb {1}}_{{\mathcal {G}}^c}(y,t)\frac{d\sigma (y)dt}{t}\le C\,r^{n-1}. \end{aligned}$$

One could say that our condition (9.2) is stronger than $G^\infty$ being prevalently close to $D_{\beta },$ and so the theorem follows from [30]. But actually, it is not so easy to link the two conditions directly. Nonetheless, we can use Chebyshev’s inequality to derive a weak condition from (9.2), which can be used as a replacement of $G^\infty$ being prevalently close to $D_{\beta }$ in the proof.

We will soon see that the condition on the operator in Theorem 9.1 can be relaxed. Again following [30], given an elliptic operator $L=-\mathop {{\text {div}}}{\mathcal {A}\nabla },$ we say that *L* is *locally sufficiently close to a constant coefficient elliptic operator* if for every choice of $\tau >0$ and $K\ge 1,$ ${\mathcal {G}}_{cc}(\tau ,K)$ is a Carleson prevalent set, where ${\mathcal {G}}_{cc}(\tau ,K)$ is the set of pairs $(x,r)\in \partial \Omega \times (0,\infty )$ such that there is a constant matrix $\mathcal {A}_0=\mathcal {A}_0(x,r)$ such that

$$\begin{aligned} \iint _{X\in W_K(x,r)}\left| \mathcal {A}(X)-\mathcal {A}_0\right| dX\le \tau r^n, \end{aligned}$$

where

9.4 $$\begin{aligned} W_K(x,r)=\left\{ X\in \Omega \cap B(x, Kr): {{\,\textrm{dist}\,}}(X,\partial \Omega )\ge K^{-1}r\right\} . \end{aligned}$$

We will actually prove Theorem 9.1 for elliptic operators *L* that are sufficiently close locally to a constant coefficient elliptic operator.

The first step of deriving weak conditions from the strong conditions on the operator and $G^\infty$ is the observation that for any integrable function *F*,  if there is a constant $C\in (0,\infty )$ such that

$$\begin{aligned} \iint _{B(x,r)\cap \Omega }\left| F(Y)\right| dY\le C\,r^{n-1} \quad \text {for }x\in \partial \Omega , r>0, \end{aligned}$$

then for any $K\ge 1,$9.5for $x\in \partial \Omega ,$ $r>0.$ This follows immediately from Fubini’s theorem and the fact that $W_K(x,r)$ defined in (9.4) is a Whitney region which is away from the boundary.

### Lemma 53

1. Let $L=-\mathop {{\text {div}}}\mathcal {A}\nabla$ be a uniformly elliptic operator which satisfies the weak DKP condition with constant $M\in (0,\infty )$ on $\Omega .$ Then *L* is locally sufficiently close to a constant coefficient elliptic operator.
2. If $G^{X_0}$ satisfies (9.2) for all *B* centered at the boundary and such that $X_0 \notin 2B,$ then for every choice of $\epsilon >0$ and $K\ge 1,$ the set
  9.7 $$\begin{aligned} {\mathcal {G}}^{X_0}(\epsilon ,K):= & {} \Big \{(x,r)\in \partial \Omega \times (0,\infty ): \, X_0 \notin B(x,2Kr) \text { and } \nonumber \\{} & {} \iint _{W_K(x,r)}\left| \nabla \ln \left( \frac{G^{X_0}}{D_{\beta }}(X)\right) \right| ^2D_{\beta }(X)dX\le \epsilon \,r^{n-1} \Big \} \end{aligned}$$
  is Carleson-prevalent.

### Proof

Both results follow from the previous observation (9.5) and Chebyshev’s inequality. In fact, for (1), we have $\mathcal {A}=\mathcal {B}+\mathcal {C}$ such that for any $x\in \partial \Omega$ and $r>0,$9.8By the Poincaré inequality, the left-hand side is bounded from below bywhere we have used the fact that $(\mathcal {B})_{W_K(y,t)}$ is a constant matrix and the definition of the set ${\mathcal {G}}_{cc}(\tau ,K).$ Combining with (9.8), we have that

$$\begin{aligned} \int _{y\in B(x,r)\cap \Omega }\int _{0<t<r}{\mathbb {1}}_{{\mathcal {G}}_{cc}(\tau ,K)^c}(y,t)\frac{dt\,d\sigma (y)}{t}\le \frac{CMK^{2n}}{\tau }r^{n-1}, \end{aligned}$$

which proves (1).

Now we justify (2). Let $\epsilon >0,$ $K\ge 1$ and $X_0\in \Omega$ be fixed, and let *B* be a ball of radius *r* centered at the boundary. Our goal is to show that

$$\begin{aligned} \int _{y\in B\cap \partial \Omega }\int _{0<t<r}{\mathbb {1}}_{{\mathcal {G}}^{X_0}(\epsilon ,K)^c}(y,t)\frac{dt\,d\sigma (y)}{t}\le C_{\epsilon ,K}\sigma (B\cap \partial \Omega ). \end{aligned}$$

We discuss two cases. If $X_0 \notin 4KB,$ then since $G^{X_0}$ satisfies (9.2) for the ball 2*KB*,  we have thatNotice that the assumption $X_0 \notin 4KB$ guarantees that $X_0\notin B(y,2Kt)$ for all $y\in B\cap \partial \Omega$ and $0<t<r.$ Therefore, if $(y,t)\in {\mathcal {G}}^{X_0}(\epsilon ,K)^c,$ then

$$\begin{aligned} \iint _{W_K(y,t)}\left| \nabla \ln \left( \frac{G^{X_0}}{D_{\beta }}\right) (Y)\right| ^2D_{\beta }(Y)dY>\epsilon \,r^{n-1}. \end{aligned}$$

From this, it follows that

9.9 $$\begin{aligned} \int _{y\in B\cap \partial \Omega }\int _{0<t<r}{\mathbb {1}}_{{\mathcal {G}}^{X_0}(\epsilon ,K)^c}(y,t)\frac{dt\,d\sigma (y)}{t}\le \frac{CK^{2n-1}}{\epsilon }\sigma (B\cap \partial \Omega ). \end{aligned}$$

Now let us deal with the case where $X_0 \in 4KB.$ For $x\in B \cap \partial \Omega ,$ we define $B_x:= B(x,|x-X_0|/20K).$ Since $\{B_x\}_{x\in B}$ covers $B \cap \partial \Omega ,$ we can find a non-overlapping subcollection $\{B_{i}\}_{i\in I}$ such that $\{5B_{i}\}_{i\in I}$ covers $B \cap \partial \Omega .$ We write $r_i>0$ for the radius of $B_i$ and we define

$$\begin{aligned} S:= (B\cap \partial \Omega ) \times (0,r) {\setminus } \bigcup _{i\in I} (5B_i\cap \partial \Omega ) \times (0,5r_i) \end{aligned}$$

We have

$$\begin{aligned}{} & {} \int _{y\in B\cap \partial \Omega }\int _{0<t<r}{\mathbb {1}}_{{\mathcal {G}}^{X_0}(\epsilon ,K)^c}(y,t)\frac{dt\,d\sigma (y)}{t}\\{} & {} \quad \le \sum _{i\in I} \int _{y\in 5B_i\cap \partial \Omega }\int _{0<t<5r_i}{\mathbb {1}}_{{\mathcal {G}}^{X_0}(\epsilon ,K)^c}(y,t)\frac{dt\,d\sigma (y)}{t} \\{} & {} \qquad + \iint _{S} {\mathbb {1}}_{{\mathcal {G}}^{X_0}(\epsilon ,K)^c}(y,t)\frac{dt\,d\sigma (y)}{t} =: T_1 + T_2. \end{aligned}$$

Since $X_0 \notin 20KB_i,$ we can apply (9.9), and we have

$$\begin{aligned} T_1 \le C_{K,\epsilon } \sum _{i\in I} \sigma (5B_i \cap \partial \Omega ) \lesssim \sum _{i\in I} \sigma (B_i \cap \partial \Omega ) \le \sigma (2B \cap \partial \Omega ) \lesssim \sigma (B \cap \partial \Omega ), \end{aligned}$$

because $\{B_i\}$ is a non-overlapping and included in 2*B*. It remains to prove a similar bound on $T_2.$ Remark first that

$$\begin{aligned}S \subset \{(y,t) \in \partial \Omega \times (0,r): \, |y-X_0|/100K < t\},\end{aligned}$$

and therefore

$$\begin{aligned} T_2 \le \int _{0}^r \int _{y\in B(X_0,100Kt) \cap \partial \Omega } \frac{d\sigma (y)\, dt}{t} \le C K^{n-1} r^{n-1} \lesssim \sigma (B \cap \partial \Omega ). \end{aligned}$$

The lemma follows. $\square$

Before we continue, we need to adapt Theorem 2.19 in [30] to our situation, that is we want to construct a positive solution in a domain which is the limit of a sequence of domains.

### Lemma 54

Let $\Omega _k$ be a sequence of 1-sided Chord-Arc domains in $\mathbb {R}^n$ with uniform 1-sided CAD constants. Let $\partial \Omega _k$ be its Ahlfors regular boundary equipped with an Ahlfors regular measure $\sigma _k$ (such that the constant in (1.1) is uniform in *k*).

Assume that $0\in \partial \Omega _k$ and ${{\,\textrm{diam}\,}}\Omega _k \ge 2^k.$ Moreover,  assume that the $\partial \Omega _k$ and $\Omega _k$ converges to $E_{\infty }$ and $\Omega _{\infty }$ locally in the Hausdorff distance,  that is,  for any $j\in {\mathbb {N}},$ we have

$$\begin{aligned} \lim _{k\rightarrow \infty } d_{0,2^j}(E_{\infty },\partial \Omega _k) = 0 \quad \text {and}\quad \lim _{k\rightarrow \infty } d_{0,2^j}(\Omega _{\infty },\Omega _k) = 0. \end{aligned}$$

Here,  for a couple of sets (*E*, *F*),  we define the Hausdorff distance

$$\begin{aligned} d_{0,2^j}(E,F):= \sup _{x\in E \cap B(0,2^j)} {{\,\textrm{dist}\,}}(x,F) + \sup _{y\in F \cap B(0,2^j)} {{\,\textrm{dist}\,}}(y,E). \end{aligned}$$

Then $E_{\infty } = \partial \Omega _{\infty },$ $E_{\infty }$ is an unbounded $(n-1)$-Ahlfors regular set,  $\Omega _{\infty }$ is a 1-sided Chord-Arc Domain. Moreover,  if the Radon measure $\sigma$ is any weak-* limit of the $\sigma _k,$ then $\sigma$ is an Ahlfors regular measure on $E_{\infty } = \partial \Omega _{\infty }.$

Let $Y_0$ be a corkscrew point of $\Omega _{\infty }$ for the boundary point 0 at the scale 1. If $L_k = - {{\,\textrm{div}\,}}A_k \nabla$ and $L_{\infty } = -{{\,\textrm{div}\,}}A_{\infty } \nabla$ are operators—in $\Omega _k$ and $\Omega _{\infty }$ respectively—that satisfies

$$\begin{aligned} \lim _{k\rightarrow \infty } \Vert A_k - A_{\infty }\Vert _{L^1(B)} = 0 \quad \text {for any ball }B\text { such that }2B \subset \Omega _{\infty }, \end{aligned}$$

and if $u_k$ are positive solutions in $\Omega _k \cap B(0,2^{k+1})$ to $L_k u_k = 0$ with ${{\,\textrm{Tr}\,}}u_k=0$ on $\partial \Omega _k\cap B(0,2^{k+1}),$ then the sequence of functions $v_k:= u_k/u_k(Y_0)$ converges,  uniformly on every compact subset of $\Omega _{\infty },$ and in $W^{1,2}_{{{\,\textrm{loc}\,}}}(\Omega _{\infty }),$ to $G^\infty ,$ the unique Green function with pole at infinity which verifies $G^\infty (Y_0) = 1.$

### Proof

The geometric properties of $E_{\infty }$ and $\Omega _{\infty }$ can be derived verbatim as in the proof of Theorem 2.19 in [30]. The uniform convergence of a subsequence of $v_k$ on any compact set $K\Subset \Omega _{\infty }$ follows from the standard argument of uniform boundedness of $\left\{ v_k\right\}$ on *K*,  and Hölder continuity of solutions. The Caccioppoli inequality would give the weak convergence of another subsequence of $v_k$ to some $v_{\infty }$ in $W_ {{{\,\textrm{loc}\,}}}^{1,2}(\Omega _{\infty }).$ This is enough to show that $v_{\infty }\in W_{{{\,\textrm{loc}\,}}}^{1,2}(\Omega _{\infty })\cap C(\overline{\Omega _{\infty }})$ is a weak solution of $L_{\infty } v_{\infty }=0$ in $\Omega _{\infty },$ as we can write

$$\begin{aligned} \iint _{\Omega _{\infty }}A_{\infty }\nabla v_{\infty }\cdot \nabla \varphi dX= & {} \iint _{\Omega _{\infty }}A_{\infty }(\nabla v_{\infty }-\nabla v_k)\cdot \nabla \varphi \,dX \\{} & {} +\iint _{\Omega _{\infty }}(A_{\infty }-A_k)\nabla v_k\cdot \nabla \varphi \,dX \end{aligned}$$

for every $\varphi \in C_0^\infty (\Omega _{\infty })$ and any *k* sufficiently big so that ${{\,\textrm{supp}\,}}\varphi \subset \Omega _k\cap B(0,2^{k+1}).$ Therefore, $v_{\infty }=G^\infty$ is the Green function with pole at infinity for $L_{\infty }$ in $\Omega _{\infty }$ and normalized so that $G^\infty (Y_0)=1.$

That $v_k$ converges to $G^\infty$ (strongly) in $W_{{{\,\textrm{loc}\,}}}^{1,2}(\Omega _{\infty })$ needs more work, but we can directly copy the proof of Lemma 2.29 in [30]. Roughly speaking, for any fixed ball *B* with $4B\subset \Omega ,$ we would need to introduce an intermediate function $V_k,$ which satisfies $L_kV_k=0$ in $B_\rho$ for some $\rho \in (r,2r),$ and $V_k=v_k$ on the sphere $\partial B_\rho .$ We refer the readers to [19] for the details. $\square$

We shall need the following result on the compactness of closed sets, which has been proved in [20].

### Lemma 55

[20, Lemma 8.2] Let $\left\{ E_j\right\}$ be a sequence of non-empty closed subsets of $\mathbb R^n,$ and suppose that there exists an $r>0$ such that $E_j\cap B(0,r)\ne \emptyset$ for all *j*. Then there is a subsequence of $\left\{ E_j\right\}$ that converges to a nonempty closed subset *E* of $\mathbb R^n$ locally in the Hausdorff distance.

Now we are ready to prove the main theorem of this section.

### Proof of Theorem 9.1

We prove that $\partial \Omega$ is uniformly rectifiable by showing that $\Omega _{\text {ext}}$ satisfies the corkscrew condition (see Lemma 2.13). Following the proof of Theorem 7.1 in [30], it suffices to show that the set ${\mathcal {G}}_{CB}(c)$ is Carleson-prevalent for some $c>0,$ where ${\mathcal {G}}_{CB}(c)$ is the set of pairs $(x,r)\in \partial \Omega \times (0,\infty )$ such that we can find $Z_1,Z_2\in B(x,r),$ that lie in different connected components of $\mathbb R^n{\setminus }\partial \Omega ,$ and such that ${{\,\textrm{dist}\,}}(Z_i,\partial \Omega )\ge cr$ for $i=1,2.$ To do that, we will rely on the fact that, on 1-sided CAD domains, if the elliptic measure is comparable to the surface measure, then the complement $\Omega _{\text {ext}}$ satisfies the corkscrew condition, which is implied by the main result of [38].

Thanks to Lemma 9.6, for each choice of $\epsilon >0$ and $M\ge 1,$ the sets ${\mathcal {G}}^{X_0}(\epsilon ,M)$ and ${\mathcal {G}}_{cc}(\epsilon ,M)$ are Carleson-prevalent. So it suffices to show that

9.12 $$\begin{aligned} {\mathcal {G}}^{X_0}(\epsilon ,M)\cap {\mathcal {G}}_{cc}(\epsilon ,M)\subset {\mathcal {G}}_{CB}(c) \quad \text {for some }c>0,\epsilon >0, \text { and }M\ge 1. \nonumber \\ \end{aligned}$$

We prove by contradiction. Assume that (9.12) is false, then for $c_k=\epsilon _k=M_k^{-1}=2^{-k},$ we can find a 1-sided NTA domain $\Omega _k$ bounded by an Ahlfors regular set $\partial \Omega _k,$ a point $X_k\in \Omega _k$ (or $X_k\in \Omega _k\cup \left\{ \infty \right\}$ when $\Omega$ is unbounded), an elliptic operator $L_k=-\mathop {{\text {div}}}\mathcal {A}_k\nabla$ that is locally sufficiently close to a constant coefficient elliptic operator, and a pair $(x_k,r_k)\in \partial \Omega _k\times (0,\infty )$ for which

$$\begin{aligned} (x_k,r_k)\in {\mathcal {G}}^{X_k}(\epsilon _k,M_k)\cap {\mathcal {G}}_{cc}(\epsilon _k,M_k){\setminus }{\mathcal {G}}_{CB}(c_k). \end{aligned}$$

By translation and dilation invariance, we can assume that $x_k=0$ and $r_k=1.$ Notice that $(0,1)\in {\mathcal {G}}^{X_k}(\epsilon _k,M_k)$ implies that $X_k\notin B(0,2^k),$ and in particular, ${{\,\textrm{diam}\,}}(\Omega _k)\ge 2^k,$ and $X_k$ tends to infinity as $k\rightarrow \infty .$

By Lemma 9.11, we can extract a subsequence so that $\Omega _k$ converges to a limit $\Omega _{\infty }.$ By Lemma 9.10, $\Omega _{\infty }$ is 1-sided NTA, $\partial \Omega _k$ converges to $\partial \Omega _{\infty }$ which is Ahlfors regular. Moreover, by Lemma 9.11, we can extract a further subsequence so that the Ahlfors regular measure $\sigma _k$ given on $\partial \Omega _k$ converges weakly to an Ahlfors regular measure $\sigma .$ Since $(0,1)\in {\mathcal {G}}_{cc}(2^{-k},2^k),$ $\mathcal {A}_k$ converges to some constant matrix $\mathcal {A}_0$ in $L^1_{{{\,\textrm{loc}\,}}}(\Omega _{\infty }).$

Choose a corkscrew point $Y_0\in \Omega _{\infty }$ for some ball $B_0$ centered on $\partial \Omega _{\infty },$ and let $G_k=G_k^{X_k}$ be the Green function for $L_k$ in $\Omega _k,$ normalized so that $G_k(Y_0)=1.$ Since $L_kG_k=0$ in $\Omega _k\cap B(0,2^k),$ Lemma 9.10 asserts that $G^k$ converges to the Green function $G=G_{\infty }^\infty$ with pole at infinity for the constant-coefficient operator $L_0=-\mathop {{\text {div}}}\mathcal {A}_0\nabla ,$ uniformly on compact sets of $\Omega _{\infty },$ and in $W_{{{\,\textrm{loc}\,}}}^{1,2}(\Omega _{\infty }).$ Since $\sigma _k\rightharpoonup \sigma ,$ $D_k=D_{\beta ,\sigma _k}$ converges to $D=D_{\beta ,\sigma }$ uniformly on compact sets of $\Omega _{\infty },$ and so does $\nabla D_k$ to $\nabla D.$ Since $(0,1)\in {\mathcal {G}}^{X_k}(2^{-k},2^k),$

9.13 $$\begin{aligned} \iint _{W_{2^k}(0,1)}\left| \frac{\nabla G_k}{G_k}-\frac{\nabla D_k}{D_k}\right| ^2D_k(X)dX\le 2^{-k} \qquad \text {for all } k\in {\mathbb {Z}}_+, \end{aligned}$$

where $W_{2^k}(0,1)$ is the Whitney region defined as in (9.4) for $\Omega _k.$ Fix any compact set $K\Subset \Omega _{\infty }.$ We claim that

9.14 $$\begin{aligned} \lim _{k\rightarrow \infty } \iint _{K}\left| \frac{\nabla G_k}{G_k}-\frac{\nabla D_k}{D_k}\right| ^2D_k(X)dX=\iint _{K}\left| \frac{\nabla G}{G}-\frac{\nabla D}{D}\right| ^2D(X)dX. \end{aligned}$$

In fact, since *G* is a positive solution of $L_0 G=0$ in $\Omega _{\infty }$ with $G(Y_0)=1,$ the Harnack inequality implies that $G\ge c_0$ on *K* for some $c_0>0.$ Then the uniform convergence of $G_k$ to *G* on *K* implies that for *k* large enough, $\left\{ G_k^{-1}\right\}$ is uniformly bounded on *K*,  and so $G_k^{-1}$ converges uniformly to $G^{-1}$ on *K*. Then (9.14) follows from the fact that $\nabla G_k$ converges to $\nabla G$ in $L^2(K),$ the uniform convergence of $G_k^{-1}$ to $G^{-1}$ on *K*,  and the uniform convergences of $\nabla D_k$ and $D_k^{-1}$ to $\nabla D$ and $D^{-1}.$

Now by (9.13) and (9.14), we get that

$$\begin{aligned}\iint _K\left| \nabla \ln \left( \frac{G}{D}\right) (X)\right| ^2D(X)dX=0,\end{aligned}$$

and so $G=CD_{\beta ,\sigma }$ in $\Omega _{\infty }.$ We can copy the proof of Theorem 7.1 of [30] verbatim from now on to conclude that this leads to a contradiction. Roughly speaking, $G=CD_{\beta ,\sigma }$ would imply that the elliptic measure $\omega ^\infty$ for $L_0,$ with a pole at $\infty ,$ is comparable to $\mathcal {H}_{|\partial \Omega _{\infty }}^{n-1}.$ Then by [38] Theorem 1.6 one can conclude that $\partial \Omega _{\infty }$ is uniformly rectifiable, and hence $\mathbb R^n{\setminus }\overline{\Omega }_{\infty }$ satisfies the corkscrew condition, which contradicts the assumption that $(0,1)=(x_k,r_k)\notin {\mathcal {G}}_{CB}(c_k).$ $\square$

## Assuming that $\Omega$ is semi-uniform is not sufficient

In this subsection, we will give an example of a domain where the harmonic measure on $\partial \Omega$ is $A_{\infty }$-absolutely continuous with respect to the $(n-1)$-dimensional Hausdorff measure, but where Theorem 1.12 fails. It is known that the harmonic measure is $A_{\infty }$-absolute continuous with respect to the surface measure whenever the domain $\Omega$ is semi-uniform and its boundary is $(n-1)$-Ahlfors regular and uniformly rectifiable (see [2, Theorem III]). The notion of semi-uniform domain is given by the next definition.

### Definition 56

(*Semi-uniform domains*) We say that $\Omega$ is semi-uniform if it satisfies the corkscrew condition and (see Definition 2.8) if for every $\Lambda \ge 1,$ there exists $C_\Lambda >0$ such that for any $\rho >1$ and every pair of points $(X,x) \in \Omega \times \partial \Omega$ such that $\left| X-x\right| <\Lambda \rho ,$ there exists a Harnack chain of length bounded by $C_\Lambda$ linking *X* to one of the corkscrew points for *x* at scale $\rho .$

Semi-uniform domains were first introduced by Aikawa and Hirata in [7] using *cigar curves*. The two definitions of semi-uniform domains are known to be equivalent, see for instance, [2, Theorem 2.3].

Our counterexample is constructed in $\mathbb {R}^2$ for simplicity but can easily be extended to any dimension.

**Fig. 1:**
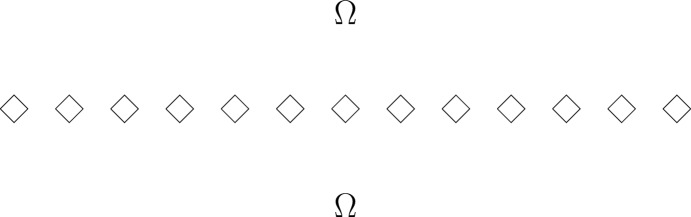
The domain $$\Omega $$

Our domain (see Fig. 1) will be

$$\begin{aligned} \Omega := \mathbb {R}^2 {\setminus } \bigcup _{k\in {{\mathbb {Z}}}} \Big \{(x,t)\in \mathbb {R}^2, |x-2k|+|t| < \frac{1}{2}\Big \}. \end{aligned}$$

Note that $\partial \Omega$ is uniformly rectifiable, but the domain contains two parts $(\Omega \cap \mathbb {R}^2_+$ and $\Omega \cap \mathbb {R}^2_-)$ which are not well connected to each other, that is, this domain does not satisfy the Harnack Chain Condition (see Definition 2.9). We let the reader check that the domain is still semi-uniform.

Due to the lack of Harnack chains, the space $\Omega$ does not have a unique—up to constant—Green function with pole at $\infty .$ If we take the pole at $t \rightarrow -\infty ,$ then we can construct a positive function *G* which will be bounded on $\Omega \cap \mathbb {R}^2_+,$ and we shall prove that this is incompatible with our estimate (1.17) that says that $\frac{\partial _t G}{G}$ is “close” to $\frac{1}{t}$ when *t* is large enough.

### Construction of *G*

The goal now will be to construct a positive function in $\Omega ,$ which is morally the Green function with pole at $t=-\infty .$ We could have used the usual approach, that is taking the limit when *n* goes to infinity of—for instance—$G(X,X_n)/G(X_0,X_n)$ in the right sense, where *G* is the Green function on $\Omega$ for the Laplacian, and $X_n := (1,n).$ However, the authors had difficulty proving the 2-periodicity in *x* of the limit and didn’t know where to find the right properties in the literature (as our domains are unbounded). So we decided to make the construction from scratch.

We want to work with the Sobolev space

$$\begin{aligned} W= & {} \Big \{u\in W^{1,2}_{loc}({\overline{\Omega }}), \, u(x,t) = u(x+2,t), \, u(-x,t) = u(x,t) \text { for }(x,t)\in \Omega , \\{} & {} \, \iint _{S_0} |\nabla u(x,t)|^2 dx \, dt < +\infty \Big \}. \end{aligned}$$

Here and in the sequel $S_k$ is the strip $\Omega \cap \Big ( [k,k+1) \times \mathbb {R}\Big ).$ Note that due to the 2-periodicity in *x* and the symmetry, the function $u\in W$ is defined on $\mathbb {R}^2$ as soon as *u* is defined on any of the sets $S_k.$ We will also need

$$\begin{aligned}W^+:= \{u_{|\Omega \cap \mathbb {R}^2_+}, \, u \in W\} \text { and } W_0:= \{u\in W, \, {{\,\textrm{Tr}\,}}(u) = 0 \text { on } \partial \Omega \}.\end{aligned}$$

We let the reader check that the quantity

$$\begin{aligned}\Vert u\Vert _W := \left( \iint _{S_0} |\nabla u(x,t)|^2 dx \, dt \right) ^\frac{1}{2}\end{aligned}$$

is a norm on the space $W_0,$ and the couple $(W_0,\Vert .\Vert _W)$ is a Hilbert space.

The bilinear form

$$\begin{aligned}a(u,v):= \iint _{S_0} \nabla u \cdot \nabla v \, dt \, dx\end{aligned}$$

is continuous and coercive on $W_0,$ so for any $k\in {\mathbb {N}},$ there exists $\widetilde{G}_k \in W_0$ such that

10.2 $$\begin{aligned} a(\widetilde{G}_k, v ) = \iint _{S_0} \nabla \widetilde{G}_k \cdot \nabla v \, dx \, dt = 2^{-k} \int _0^1 \int _{-2^{k+1}}^{-2^k} v(x,t) \, dt\, dx \quad \text {for } v\in W_0.\nonumber \\ \end{aligned}$$

The first key observation is:

#### Proposition 57

$\widetilde{G}_k \in W_0$ is a positive weak solution to $-\Delta u = 0$ in $\Omega \cap \{t>-2^k\}.$

#### Proof

The fact that $\widetilde{G}_k$ is nonnegative is a classical result that relies on the fact that $u\in W_0 \implies |u|\in W_0$ and the bilinear form *a*(*u*, *v*) is coercive. See for instance [22], (10.18)–(10.20).

In order to prove that $\widetilde{G}_k$ is a solution in $\Omega \cap \{t>-2^k\},$ take $\phi \in C^\infty _0(\Omega \cap \{t>-2^k\}).$ For $j\in {\mathbb {Z}},$ let $\phi _j$ be the only symmetric and 2-periodic function in *x* such that $\phi _j = \phi$ on $S_j.$ Observe that $\phi _j$ is necessary continuous, and so $\phi _j$ lies in $W_0.$ Thus

$$\begin{aligned} \iint _{\Omega } \nabla \widetilde{G}_k \cdot \nabla \phi \, dx\, dt= & {} \sum _{j\in {{\mathbb {Z}}}} \iint _{S_j} \nabla \widetilde{G}_k \cdot \nabla \phi \, dx\, dt = \sum _{j\in {{\mathbb {Z}}}} \iint _{S_j} \nabla \widetilde{G}_k \cdot \nabla \phi _j \, dx\, dt \\= & {} \sum _{j\in {{\mathbb {Z}}}} \iint _{S_0} \nabla \widetilde{G}_k \cdot \nabla \phi _j \, dx\, dt = 0 \end{aligned}$$

by (10.2), since $\phi _j =\phi \equiv 0$ on $\{t\le -2^k\}$ for all $j\in {{\mathbb {Z}}}.$

Since $\widetilde{G}_k$ is a solution, which is nonnegative and not identically equal to 0 (otherwise (10.2) would be false), the Harnack inequality (Lemma 2.15) entails that $\widetilde{G}_k$ is positive. The proposition follows. $\square$

Let $X_0:= (1,0) \in \Omega .$ From the above proposition, $\widetilde{G}_k(X_0) >0$ so we can define

10.4 $$\begin{aligned} G_k(X) := \frac{\widetilde{G}_k(X)}{\widetilde{G}_k(X_0)}. \end{aligned}$$

#### Proposition 58

For each $k\in {\mathbb {N}},$ the function $G_k(X) \in W_0$ is a positive weak solution to $-\Delta u = 0$ in $\Omega \cap \{t>-2^k\}.$ Moreover,  we have the following properties : 

(i) for any compact set $K\Subset {\overline{\Omega }},$ there exists $k:=k(K)$ and $C:=C(K)$ such that $G_j(X) \le C_K$ for all $j\ge k$ and $X\in K$ and $\{G_j\}_{j\ge k}$ is equicontinuous on *K*; 
(ii) there exists $C>0$ such that
  $$\begin{aligned} \iint _{\Omega \cap ([-2,2] \times [-1,1])} |\nabla G_k(x,t)|^2 dx\, dt \le C \quad \text {for all } k\in {\mathbb {N}}; \end{aligned}$$
(iii) there exists $C>0$ such that
  $$\begin{aligned} \Vert G_k\Vert _{W^+}^2 := \iint _{S_0 \cap \mathbb {R}^2_+} |\nabla G_k|^2 dx\, dt \le C \quad \text {for all } k\in {\mathbb {N}}. \end{aligned}$$

#### Proof

The fact that $G_k$ is a positive weak solution is given by Proposition 10.3. So it remains to prove (i), (ii) and (iii).

We start with (i). Since $G_k$ is a weak solution in $\Omega _0 := \Omega \cap [(-4,4) \times (-2,2)]$ when $k\ge 1,$ and since $\Omega _0$ is a Chord Arc Domain, we can invoke the classical elliptic theory and we can show that there exists $C>0$ such that

$$\begin{aligned} \sup _{\Omega \cap ([-2,2] \times [-1,1])} G_k \le C G_k(1,0) = C \quad \text {for all }k\ge 1, \end{aligned}$$

see for instance Lemma 15.14 in [24]. By the 2-periodicity of $G_k,$ it means that

$$\begin{aligned} \sup _{k\ge 1} \sup _{\Omega \cap ( \mathbb {R}\times [-1,1])} G_k \le C, \end{aligned}$$

and then since we can link any point of a compact $K \Subset {\overline{\Omega }}$ back to $\Omega \cap ( \mathbb {R}\times [-1,1])$ with a Harnack chain (the length of the chain depends on *K*), we have

$$\begin{aligned} \sup _{j\ge k} \sup _{K} G_j \le C_K, \end{aligned}$$

whenever $G_j$ is a solution in the interior of *K*,  which is bound to happen if $j\ge k(K)$ is large enough.

The functions $G_k$ are also Hölder continuous up to the boundary in the areas where they are solutions, so $\{G_j\}_{j\ge k}$ is equicontinuous on *K* as long as *k* is large enough so that $K \subset {\overline{\Omega }} \cap \left\{ t>-2^k\right\} .$

Point (ii) is a consequence of the Caccioppoli inequality at the boundary. We only need to prove the bound when $k\ge 2,$ since all the $G_k$ are already in $W_0$ by construction. We have by the Caccioppoli inequality at the boundary (see for instance Lemma 11.15 in [24]) that

$$\begin{aligned} \iint _{\Omega \cap ([-2,2] \times [-1,1])} |\nabla G_k(x,t)|^2 dx\, dt\lesssim & {} \iint _{\Omega \cap ([-4,4] \times [-2,2])} |G_k(x,t)|^2 dx\, dt \\\lesssim & {} \sup _{\Omega \cap ([-4,4] \times [-2,2])} |G_k(x,t)|^2 \lesssim 1. \end{aligned}$$

Point (iii) is one of our key arguments. We define $W_0^+$ as the subspace of $W^+$ that contained the functions with zero trace on $\partial (\Omega \cap \mathbb {R}^2_+).$

Since $$G_k \in W_0,$$ its restriction $$(G_k)_{|\Omega \cap \mathbb {R}^2_+}$$ is of course in $$W^+.$$ Moreover, $$G_k$$ is a solution to $$-\Delta u = 0$$ in $$\Omega \cap \mathbb {R}^2_+.$$ We can invoke the uniqueness in Lax–Milgram theorem (see Lemma 12.2 in [24], but adapted to our periodic function spaces $$W_0^+$$ and $$W^+)$$ to get that $$G_k$$ is the only weak solution to $$-\Delta u = 0$$ in $$\Omega \cap \mathbb {R}^2_+$$ for which the trace on $$\partial (\Omega \cap \mathbb {R}^2_+)$$ is $$(G_k)_{|\partial (\Omega \cap \mathbb {R}^2_+)}.$$ Moreover,

$$\begin{aligned} \Vert G_k\Vert _{W^+} \le C \Vert (G_k)_{|\partial (\Omega \cap \mathbb {R}^2_+)}\Vert _{H^{1/2}_{\partial \Omega _+}}, \end{aligned}$$

where $H^{1/2}_{\partial \Omega _+}$ is the space of traces on $\partial \Omega _+:= \partial (\Omega \cap \mathbb {R}^2_+)$ for the symmetric 2-periodic functions defined as

$$ \begin{aligned}{} & {} H^{1/2}_{\partial \Omega _+} := \Big \{ f: \, \partial \Omega _+ \mapsto \mathbb {R}\text { measurable s.t. }f\text { is symmetric } \&  ~2\text {-periodic in }x, \\{} & {} \quad \text { and } \Vert f\Vert _{H^{1/2}_{\partial \Omega _+}}:= \left( \int _{\partial \Omega _+ \cap S_0} \int _{\partial \Omega _+ \cap S_0} \frac{|f(x)-f(y)|^2}{|x-y|^{3/2}} d{\mathcal {H}}^1(x) \, d{\mathcal {H}}^1(y)\right) ^\frac{1}{2} < +\infty \Big \}. \end{aligned}$$

So in particular, we have by a classical argument that

$$\begin{aligned} \Vert (G_k)_{|\partial (\Omega \cap \mathbb {R}^2_+)}\Vert ^2_{H^{1/2}_{\partial \Omega _+}} \le C \iint _{\Omega \cap ([-2,2] \times [-1,1])} |\nabla G_k(x,t)|^2 dx\, dt.\end{aligned}$$

We conclude that

$$\begin{aligned} \iint _{S_0 \cap \mathbb {R}^2_+} |\nabla G_k|^2 dx\, dt \lesssim \iint _{\Omega \cap ([-2,2] \times [-1,1])} |\nabla G_k(x,t)|^2 dx\, dt \lesssim 1 \end{aligned}$$

by (ii). Point (iii) follows. $\square$

#### Proposition 59

There exists a symmetric (in *x*),  2-periodic (in *x*),  positive weak solution $G\in W_{{{\,\textrm{loc}\,}}}^{1,2}(\Omega )\cap C(\overline{\Omega })$ to $-\Delta G = 0$ in $\Omega$ such that $G=0$ on $\partial \Omega$ and $G(X_0) = 1$ and

10.7 $$\begin{aligned} \iint _{S_0 \cap \mathbb {R}^2_+} |\nabla G|^2 dx\, dt < +\infty . \end{aligned}$$

#### Proof

We invoke the Arzelà–Ascoli theorem—whose conditions are satisfied thanks to Proposition 10.5(i)—to extract a subsequence of $G_k$ that converges uniformly on any compact to a continuous function *G*. The fact *G* is non-negative, symmetric, 2-periodic, and satisfies $G(X_0) =1$ is immediate from the fact that all the $G_k$ are already like this. The functions $G_k$ converge to *G* in $W^{1,2}_{loc}({\overline{\Omega }})$ thanks to the Caccioppoli inequality, and then by using the weak convergence of $G_k$ to *G* in $W^{1,2}_{loc}(\overline{\Omega }),$ we can easily prove that *G* is a solution to $-\Delta u = 0$ in $\Omega$ (hence *G* is positive by the Harnack inequality, since it was already non-negative). The convergence of $G_k$ to *G* in $W^{1,2}_{loc}(\overline{\Omega })$ also allow the uniform bound on $\Vert G_k\Vert _{W^+}$ given by Proposition 10.5(iii) to be transmitted to *G*,  hence (10.7) holds. The proposition follows. $\square$

### *G* fails the estimate given in Theorem 1.12

#### Lemma 60

$\partial _t G$ is harmonic in $\Omega ,$ that is,  it is a solution of $-\Delta u=0$ in $\Omega ,$ and we have

$$\begin{aligned} \int _1^\infty \int _{0}^1 |\nabla \partial _t G|^2 dx\, dt < +\infty . \end{aligned}$$

#### Proof

Morally, we want to prove that if *G* is a solution (to $-\Delta u = 0)$, then $\nabla G \in W^{1,2},$ which is a fairly classical regularity result. The difficulty in our case is that the domain in consideration is unbounded.

Since *G* is a harmonic function (solution of the Laplacian), the function $g(x):= G(x,1)$ is smooth. We can prove the bound

$$\begin{aligned} \int _1^\infty \int _{0}^1 |\nabla \partial _x G|^2 dx\, dt\lesssim & {} \int _0^1 |g'(x)|^2 dx + \int _0^1 |g''(x)|^2 dx \\{} & {} + \int _1^\infty \int _{0}^1 |\nabla G|^2 dx\, dt < +\infty \end{aligned}$$

by adapting the proof of Proposition 7.3 in [26] to our simpler context (and invoking (10.7) and $g \in C^\infty (\mathbb {R})$ to have the finiteness of the considered quantities). In order to have the derivative on the *t*-derivative, it is then enough to observe

$$\begin{aligned}{} & {} \int _1^\infty \int _{0}^1 |\nabla \partial _t G|^2 dx\, dt \lesssim \int _1^\infty \int _{0}^1 |\partial _x \partial _t G|^2 dx\, dt + \int _1^\infty \int _{0}^1 |\partial _t \partial _t G|^2 dx\, dt \\{} & {} \quad = \int _1^\infty \int _{0}^1 |\partial _t \partial _x G|^2 dx\, dt + \int _1^\infty \int _{0}^1 |\partial _x \partial _x G|^2 dx\, dt \\{} & {} \quad \lesssim \int _1^\infty \int _{0}^1 |\nabla \partial _x G|^2 dx\, dt < +\infty , \end{aligned}$$

where we use the fact that *G* is a solution to $-\Delta u = 0$—i.e. $\partial _t \partial _t G = - \partial _x \partial _x G$—for the second line. The lemma follows. $\square$

We will also need a maximum principle, given by

#### Lemma 61

If *u* is a symmetric (in *x*),  2-periodic (in *x*) harmonic function in $\mathbb {R}\times (t_0,\infty )$ that satisfies

10.10 $$\begin{aligned} \int _{t_0}^\infty \int _{0}^1 |\nabla u|^2 dx\, dt < +\infty , \end{aligned}$$

then *u* has a trace—denoted by ${{\,\textrm{Tr}\,}}_{t_0} u$—on $\mathbb {R}\times \{t_0\}$ and

$$\begin{aligned} \inf _{y\in (0,1)} ({{\,\textrm{Tr}\,}}_{t_0} u)(y) \le u(x,t) \le \sup _{y\in (0,1)} ({{\,\textrm{Tr}\,}}_{t_0} u)(y) \quad \text {for all } x\in \mathbb {R}, \, t>t_0. \end{aligned}$$

#### Proof

The existence if the trace—in the space $W^{2,\frac{1}{2}}(\mathbb {R}\times \{t_0\})$—is common knowledge. The proof of Lemma 12.8 in [24] (for instance) can be easily adapted to prove our case. $\square$

#### Lemma 62

There exists $C\ge 1$ such that

$$\begin{aligned} C^{-1} \le G(x,t) \le C \quad \text {for } x\in \mathbb {R}, \, t\ge 1. \end{aligned}$$

#### Proof

Since $G(1,0) = G(X_0) = 1$ and *G* is a positive solution, the Harnack inequality implies that $C^{-1} \le G(x,1) \le C$ for $x\in [0,1].$ Since *G* is symmetric and 2-periodic in *x*,  we have $C^{-1} \le G(x,1) \le C$ for $x\in \mathbb {R}.$ We conclude with the maximum principle (Lemma 10.9), since the bound (10.10) is given by (10.7). $\square$

#### Lemma 63

For every $c>0,$ there exists $t_0\ge 1$ such that

$$\begin{aligned} \partial _t G(x,t) \le \frac{c}{t} \quad \text {for all }x\in \mathbb {R},~t\ge t_0. \end{aligned}$$

#### Proof

Let *x* be fixed. Since *G* is symmetric and 2-periodic in *x*,  we can assume without loss of generality that $x\in (0,1).$ Then recall that $\partial _t G$ is a weak solution in $\Omega ,$ so in particular, we have the Moser estimate and the Caccioppoli inequality, which give10.13by Lemma 10.11. Moreover, $\partial _t G$ is Hölder continuous, that is,10.14by (10.13).

We pick $t_0 \ge 8$ such that $2C'(t_0)^{-\alpha } \le c.$ Assume by contradiction that there exist $x\in (0,1)$ and $t\ge t_0$ such that $\partial _t G(x,t) \ge c/t,$ then

$$\begin{aligned} \inf _{y\in \mathbb {R}} \partial _t G(y,t)= & {} \inf _{y\in (0,1)} \partial _t G(y,t) \ge \partial _t G(x,t) - \sup _{y\in (0,1)} |\partial _t G(x,t) - \partial _t G(y,t)| \\\ge & {} \frac{c - C't^{-\alpha }}{t} \ge \frac{c}{2t} \end{aligned}$$

by our choice of $t_0.$ Since $\partial _t G$ is a solution that satisfies (10.10)—see Lemma 10.8—the maximum principle given by Lemma 10.9 entails that

$$\begin{aligned}\partial _t G(y,s) \ge \frac{c}{2t} \quad \text {for } y \in \mathbb {R}, \, s>t,\end{aligned}$$

which implies

$$\begin{aligned}\int _0^1 \int _t^{\infty } |\nabla G(y,s)|^2 ds \, dy = +\infty ,\end{aligned}$$

which is in contradiction with (10.7). We conclude that for every $x\in (0,1)$ and $t\ge t_0,$ we necessary have $\partial _t G \le c/t.$ The lemma follows.$\square$

#### Lemma 64

For any $\beta >0,$ there exist $t_0 \ge 1$ and $\epsilon >0$ such that

10.16 $$\begin{aligned} \left| \frac{ \partial _t G(x,t)}{G(x,t)} - \frac{\partial _t D_{\beta }(x,t)}{D_{\beta }(x,t)} \right| \ge \frac{\epsilon }{t} \quad \text {for } x\in \mathbb {R}, \, t\ge t_0. \end{aligned}$$

#### Proof

The set $\partial \Omega$ is $(n-1)$-Ahlfors regular, so (1.4) gives the equivalence $D_{\beta }(X) \approx {{\,\textrm{dist}\,}}(X,\partial \Omega )$ for $X\in \Omega ,$ and hence the existence of $C_1>0$ (depending on $\beta$ and *n*) such that

10.17 $$\begin{aligned} (C_1)^{-1} t \le D_{\beta }(x,t) \le C_1 D_{\beta +2}(x,t) \le (C_1)^2 t \quad \text {for } x\in \mathbb {R}, \, t\ge 1. \end{aligned}$$

Check then that

$$\begin{aligned} \partial _t D_{\beta }(x,t) = \frac{d+\beta }{\beta } D_{\beta }^{1+\beta }(x,t) \int _{(y,s) \in \partial \Omega } |(x,t)-(y,s)|^{-d-\beta -2} (t-s) \, d\sigma (y,s) \end{aligned}$$

In particular, since $s\le \frac{1}{2}$ whenever $(y,s) \in \partial \Omega ,$ we have, for $(x,t) \in \mathbb {R}\times [1,\infty ),$ that

$$\begin{aligned}{} & {} \partial _t D_{\beta }(x,t) \\{} & {} \quad \ge \Big ( t - \frac{1}{2} \Big ) \frac{n+\beta -1}{\beta } D^{1+\beta }_{\beta }(x,t) \int _{(y,s) \in \partial \Omega } |(x,t)-(y,s)|^{-n-\beta -1} \, d\sigma (y,s) \\{} & {} \quad \ge \frac{t}{2} \frac{n+\beta -1}{\beta } D^{1+\beta }_{\beta }(x,t) D^{-\beta -2}_{\beta +2}(x,t) \ge c_{\beta ,n} \end{aligned}$$

for some $c_{\beta ,n}>0,$ by (10.17). In conclusion, using (10.17) again, we have the existence of $c_1>0$ such that

10.18 $$\begin{aligned} \frac{\partial _t D_{\beta }(x,t)}{D_{\beta }(x,t)} \ge \frac{c_1}{t} \quad \text {for } x\in \mathbb {R}, \, t\ge 1. \end{aligned}$$

Let $C_2$ be the constant in Lemma 10.11. Thanks to Lemma 10.12, there exists $t_0\ge 1$ such that $\partial _t G(x,t) \le c_1/(2C_2t)$ for any $x\in \mathbb {R}$ and $t\ge t_0,$ which means that

10.19 $$\begin{aligned} \frac{\partial _t G(x,t)}{G(x,t)} \le \frac{c_1}{2t} \quad \text {for } x\in \mathbb {R}, \, t\ge t_0. \end{aligned}$$

The combination of (10.18) and (10.19) gives (10.16) for $\epsilon = c_1/2.$ $\square$

#### Lemma 65

The positive solution *G* does not satisfy (1.13),  proving that assuming that $\Omega$ is semi-uniform is not sufficient for Theorem 1.12.

#### Proof

Let $B_r$ be the ball of radius *r* centered at $(0,\frac{1}{2}) \in \partial \Omega ,$ and take $r\ge 2t_0,$ where $t_0\ge 1$ is the value from Lemma 10.15. We have

$$\begin{aligned} \iint _{\Omega \cap B_r} \left| \frac{\nabla G}{G} - \frac{\nabla D_{\beta }}{D_{\beta }} \right| ^2 D_{\beta } \, dx\, dt\ge & {} \iint _{B_r \cap \{t\ge t_0\}} \left| \frac{\nabla G}{G} - \frac{\nabla D_{\beta }}{D_{\beta }} \right| ^2 D_{\beta } \, dx\, dt \\\ge & {} C^{-1}\epsilon ^2\iint _{B_r \cap \{t\ge t_0\}} \frac{dx\,dt}{t} \end{aligned}$$

by (10.16) and (1.4). We conclude that

$$\begin{aligned} \frac{1}{\sigma (B_r)} \iint _{\Omega \cap B_r} \left| \frac{\nabla G}{G} - \frac{\nabla D_{\beta }}{D_{\beta }} \right| ^2 D_{\beta } \, dx\, dt > rsim \ln \Big (\frac{r}{t_0} \Big ) \rightarrow +\infty \text { as } r\rightarrow \infty , \end{aligned}$$

which means that *G* does not satisfy (1.13). The lemma follows. $\square$

## Footnotes

1 We actually only need the Carleson condition on the last column of ${\mathcal {B}}$ and ${\mathcal {C}}$ (instead of the full matrix).
